# Associations between special diet and incidence risk of osteoporosis: a Mendelian randomization study

**DOI:** 10.3389/fpubh.2024.1364735

**Published:** 2024-05-30

**Authors:** Changwen Zhou, Lixue Yang, Ce Liu, Hongzhong Ma, Feng Yang, Liheng Chen

**Affiliations:** ^1^The First Clinical Medical Research Institute, Shaanxi University of Chinese Medicine, Xianyang, Shaanxi, China; ^2^Affiliation Hospital of Shaanxi University of Chinese Medicine, Xianyang, Shaanxi, China

**Keywords:** osteoporosis, dietary habits, gluten-free diet, Mendelian randomization, celiac disease

## Abstract

**Introduction:**

Osteoporosis is a prevalent challenge in clinical orthopedics, affecting a significant percentage of individuals aged 50 and above. The goal of this study was to comprehensively understand the relationships between a specialized dietary regimen and the risk of developing osteoporosis.

**Methods:**

This study employed extensive genome-wide association study (GWAS) summary statistics derived from the UK Biobank. It encompassed 8 kinds of special diets and 7 datasets pertaining to osteoporosis and associated symptoms. The principal analytical approach employed was the inverse-variance weighted method. Additionally, sensitivity analysis was employed to elucidate the diverse multiplicity patterns observed in the final model.

**Results:**

Our results showed that there is significant evidence that a gluten-free diet is associated with osteoporosis [odds ratio (OR): 1.080, 95% confidence interval (CI): 1.048–1.112, *p* = 4.23E-07)]. Furthermore, there exists a suggestive link between the three distinct dietary approaches and osteoporosis [(OR: 0.949, 95%CI: 0.929–0.970, *p* = 3.00E-06) for comprehensive consumption; (OR: 1.053, 95%CI: 1.018–1.089, *p* = 2.23E-03) for abstaining from wheat consumption; (OR: 1.036, 95%CI: 1.005–1.068, *p* = 1.97E-02) for abstaining from sugar consumption]. No additional correlation between the special dietary regimens and osteoporosis has been observed.

**Conclusion:**

Our research has uncovered a notable correlation between a gluten-free diet and the occurrence of osteoporosis. Furthermore, it exerts a promoting influence on the onset of osteoporosis, which stands in direct contradiction to the therapeutic principles for Celiac Disease’s complications. As such, a novel association among these three elements is postulated.

## 1 Introduction

Osteoporosis, a challenge frequently encountered in the realm of clinical orthopedics, exerts a substantial influence on the decision-making process pertaining to numerous treatment alternatives. According to the most recent research, osteoporosis affects a noteworthy 10.2% of individuals aged 50 years and above, with an anticipated rise to 13.6% by the year 2030 (1). Nevertheless, despite the exorbitant annual expenditures reaching billions of dollars for the treatment of osteoporosis in the United States, with projected costs expected to persistently escalate (2), the problem persists. It is imperative to ascertain modifiable protective or risk factors in order to avert the onset and progression of osteoporosis.

Diet has gained significant attention among researchers as an easily obtainable and modifiable factor. Several systematic studies have demonstrated the presence of the influence of diet and nutrition on osteoporosis, although conclusive findings are hindered by factors such as heterogeneity and small sample sizes (3, 4). Current research indicates that dairy products, protein, vitamin D, vitamin K, fruits and vegetables, and adherence to a Mediterranean diet all contribute to the promotion of bone health (4, 5). However, it is important to note that eating habits encompass not only the act of food consumption but also the deliberate exclusion of specific food items. This study defines the latter as “special diets” and explores these novel avenues to comprehensively elucidate the influence of diet on osteoporosis.

In this instance, Mendelian randomization (MR) presents itself as a viable approach for deducing the interconnections amidst distinctive dietary patterns and osteoporosis. MR employs genetic variants as instrumental variables (IVs) for the exposure (such as specialized diets) to facilitate inferential associations (6). This method significantly mitigates the influence of confounding factors commonly encountered in observational studies. By virtue of the random allocation of alleles during conception, the relationship between genetic variations and disease outcomes remains less susceptible to environmental and confounding factors (7, 8).

In this investigation, summary statistics derived from genome-wide association studies (GWAS) were employed to perform a two-sample MR analysis, aiming to comprehensively delineate the relationships between a specialized dietary regimen and the risk of developing osteoporosis.

## 2 Materials and methods

### 2.1 Study design

**Figure 1:**
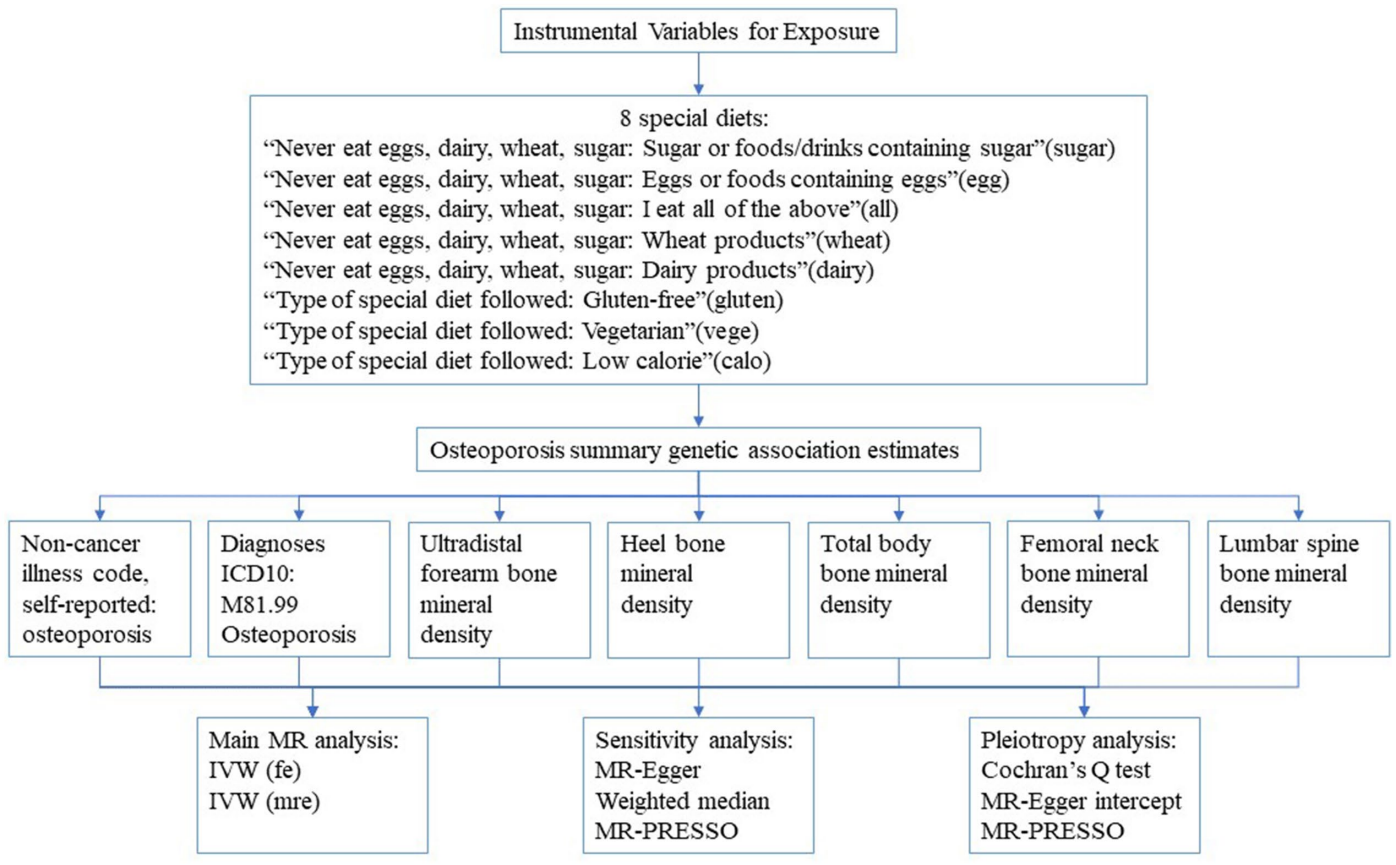
The flow chart of study design. IVW, Inverse variance weighted; fe, fixed-effects; mre, multiplicative random-effects.

Our MR study was built upon three underlying hypotheses: Firstly, genetic variations are intimately connected with the particular exposure in question. Secondly, genetic variations are not associated with any confounding variables. Lastly, genetic variations are unable to have a direct effect on the outcome, but only through the specific exposure being examined (8). The data employed in this study were sourced from previously published summary statistics of GWAS, thus obtaining ethical approval and informed consent was not required. In this study, we designated the special diet as the exposure variable and executed a series of matching analyses with osteoporosis-related indicators as individual outcomes. The specific matching process is elucidated in Figure 1.

### 2.2 Selection of instrumental variables and data source

The genetic variants pertaining to special diets were obtained from two UK Biobank cohorts consisting of approximately 461,046 and 64,949 individuals (9). The initial list comprised 8 special diets: “Never eat sugar: Sugar or foods/drinks containing sugar”(NOSUGAR), “Never eat wheat: Wheat or products containing wheat”(NOWHEAT), “Never eat dairy: Dairy or products containing dairy”(NODAIRY), “Never eat eggs: Eggs or foods containing eggs”(NOEGG), “Eggs, dairy, wheat, sugar: I eat all of the above”(ALL), “Type of special diet followed: Gluten-free”(GLUTEN), “Type of special diet followed: Vegetarian”(VEGE) and “Type of special diet followed: Low calorie”(CALO). To select valid IVs, we included single nucleotide polymorphisms (SNPs) at the genome-wide significant level (*p* < 5 × 10^−8^) (10) and applied strict cutoff values (*R*^2^ < 0.001; region size = 10,000 kb) to exclude SNPs that are in linkage disequilibrium. As NODAIRY, NOEGG, NOWHEAT, GLUTEN, VEGE and CALO have less than 5 SNPs that meet the strict threshold (*p* < 5 × 10^−8^), we used a relaxed threshold (*p* < 5 × 10^−5^; *R*^2^ < 0.001; region size = 10,000 kb) to select SNPs for these diets. Moreover, SNPs with a minimum allele frequency (MAF) of less than 0.05 were excluded as the association between these SNPs and special diets was deemed to be unstable (11). To satisfy the second and third critical hypotheses, all selected SNPs were evaluated using the PhenoScanner database (12), and none of them needed to be excluded. Additionally, we ruled out SNPs associated with multiple special diets to reduce potential pleiotropy across the SNPs (Supplementary Table S1). Lastly, *F*-statistics were employed to evaluate SNPs with weak IVs bias (13). The *F*-statistics formula is *F* = *R*^2^ × (*N*-2)/(1-*R*^2^), where *N* represents the sample size, and *R*^2^ refers to the variance of exposure explained by IVs. Only the SNP with *F*-statistics >10 were considered for inclusion in the MR analysis (11).

To evaluate the association between special diets and the incidence risk of osteoporosis in a more comprehensive manner, we aimed to include all eligible GWAS of osteoporosis by conducting an extensive search of the public Integrative Epidemiology Unit (IEU) GWAS database^1^ (14). We selected two GWAS data sets from UK Biobank. In addition to osteoporosis patients diagnosed based on hospitalization records and using diagnosis codes; we also considered the low willingness of older adult/adults patients to medical treatment, patients with suspected osteoporosis classified by the interviewer based on the description of the participants were deliberately included. On this basis, we believe that it is necessary to include bone density, a diagnostic indicator of osteoporosis, in the study to improve the reliability of the study. The specific information of the summary-level data included in this study is shown in Supplementary Table S2.

### 2.3 Statistical analysis

MR analysis utilized SNPs as proxies for predicting the impact of special diets on osteoporosis risk. The study employed the fixed-effects inverse-variance weighted (IVW) method as the primary technique to estimate the association between the genetic prediction level of special diets and osteoporosis risk (15). The IVW method combines Wald estimates for each SNP using a meta-analysis approach to derive an overall estimate of the effect of special diets on osteoporosis. The IVW method is capable of providing unbiased estimates if horizontal or vertical pleiotropy is balanced. Sensitivity analysis was conducted using the weighted median approach, which allowed for the inclusion of invalid genetic variants while still producing a consistent point estimate (16). The InSIDE hypothesis formed the basis for the MR-Egger method, which provides a valid test of the null associational hypothesis and a consistent estimate of the associational effect, even if all genetic variants are invalid IVs. However, the MR-Egger method may produce inaccurate estimates and may be significantly influenced by external genetic variants (17). Lastly, the MR-PRESSO method used a global test to assess horizontal pleiotropy and outliers, as well as to compare results before and after outliers was removed (18).

In each analysis of special diets and osteoporosis, Cochran’s Q statistics were employed to quantify the heterogeneity between IVs (19). In the event that heterogeneity is detected (*P*_Cochran’sQ_ < 0.05), the multiplicative random-effects IVW model is implemented to circumvent the bias toward weaker instrument exposure associations (20). The MR-Egger intercept test utilized the intercept term to assess pleiotropy (21). If a significant difference between the intercept term and zero exists, it is plausible that there is horizontal pleiotropy between IVs. Additionally, forest plots, scatter plots, funnel plots, and leave-one-out analysis plots were generated to depict the results with high-confidence. Specifically, the forest plot intuitively presents the impact of each SNP on the outcome, while the leave-one-out analysis determines the visual robustness of the results. The scatter plot illustrates the fitting results of various MR analyses, and the funnel plot visually evaluates the heterogeneity of IVs.

The 95% confidence interval (CI) of the odds ratio (OR) was utilized to estimate the association between special diets and osteoporosis. A suggestive correlation was established if *p* < 0.05, whereas high-confidence associations survived multiple tests with a threshold of 0.006 (= 0.05/8) by Bonferroni correction.

All data analysis in this study was executed using R software (version 4.1.3). The R packages utilized for MR analyses included TwoSampleMR (22) and MR-PRESSO (18) packages.

## 3 Results

### 3.1 Special diet and osteoporosis

**Figure 2:**
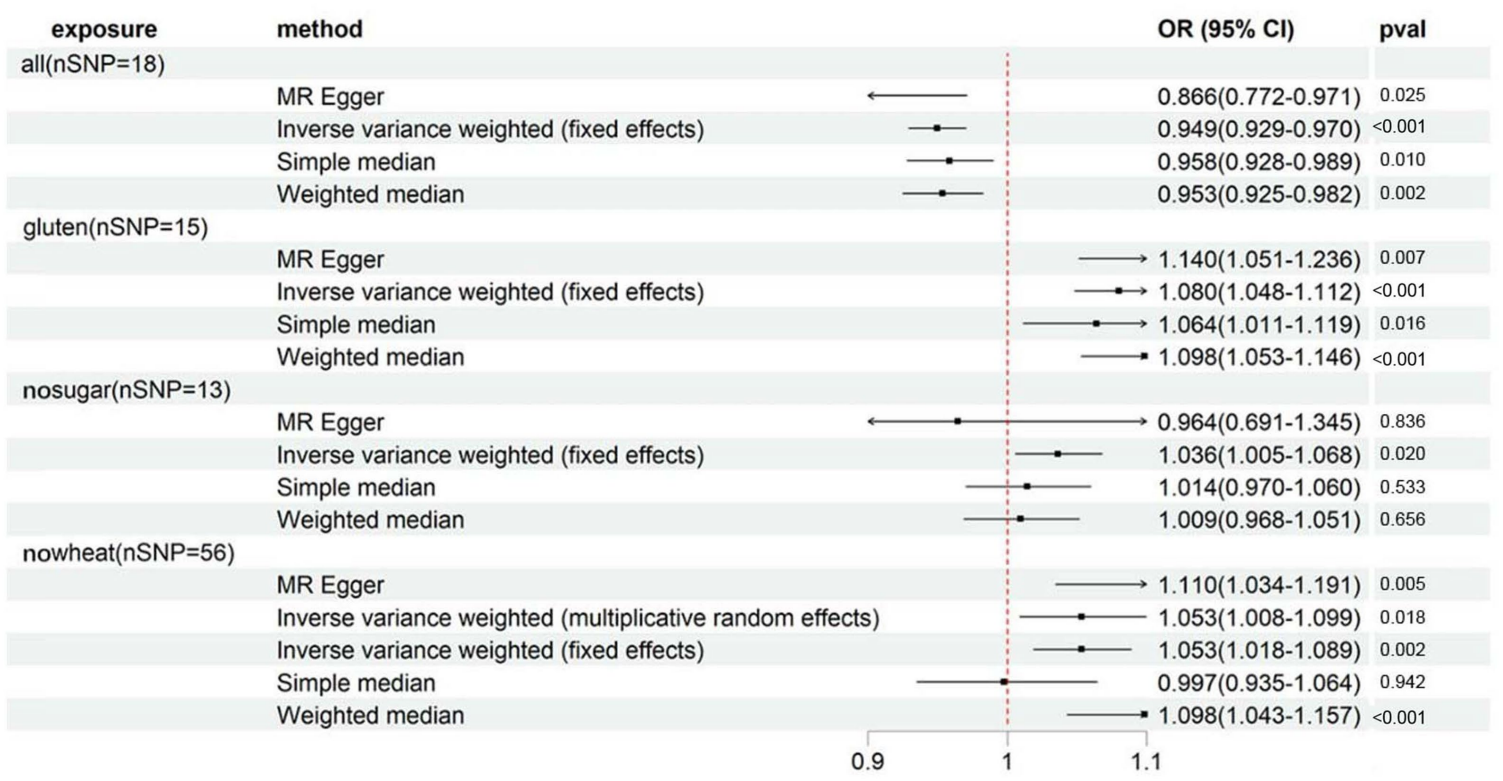
Forest plot showing results from Mendelian randomization study to assess associations between special diets and osteoporosis (self-reported). nSNP, number of single-nucleotide polymorphisms; OR, odds ratio; CI, confidence interval; all, Eggs, dairy, wheat, sugar: I eat all of the above; gluten, Type of special diet followed: Gluten-free; nosugar, Never eat sugar: Sugar or foods/drinks containing sugar; nowheat, Never eat wheat: Wheat or products containing wheat.

Supplementary Table S3 presents the distinct characteristics of 346 IVs across 8 special diets. With all IVs exhibiting F statistics exceeding 10 (minimum = 16, maximum = 672), the risk of weak instrument bias is effectively mitigated. By referencing Supplementary Table S4 and Figure 2, the fixed-effects IVW method indicated that osteoporosis (self-reported) was significantly associated with ALL (OR: 0.949, 95%CI: 0.929–0.970, *p* = 3.00E-06), GLUTEN (OR: 1.080, 95%CI: 1.048–1.112, *p* = 4.23E-07) and NOWHEAT (OR: 1.053, 95%CI: 1.018–1.089, *p* = 2.23E-03), while NOSUGAR (OR: 1.036, 95%CI: 1.005–1.068, *p* = 1.97E-02) was suggestively associated. Notably, evidence of heterogeneity was found in NOWHEAT (*P*_Cochran’s Q_ < 0.05), suggesting the possibility of fixed-effects IVW estimation bias (refer to Supplementary Table S5). However, the random-effects IVW method proposed a suggestive association between NOWHEAT and osteoporosis (self-reported). Results of sensitivity analyses, except for MR-Egger, were directionally consistent with the IVW method. The MR-Egger analysis revealed a conflicting point estimate for the association between SUGAR and osteoporosis (self-reported) in comparison to the main analysis (IVW method). No horizontal pleiotropy was observed in the MR-Egger intercept test (shown in Supplementary Table S5). Lastly, the MR-PRESSO Global Test identified no outliers in the four specific diets (refer to Supplementary Table S5). In summary, significant associations between these diets and osteoporosis (self-reported) were visually confirmed (shown in Supplementary Figures S1–S4).

**Figure 3:**
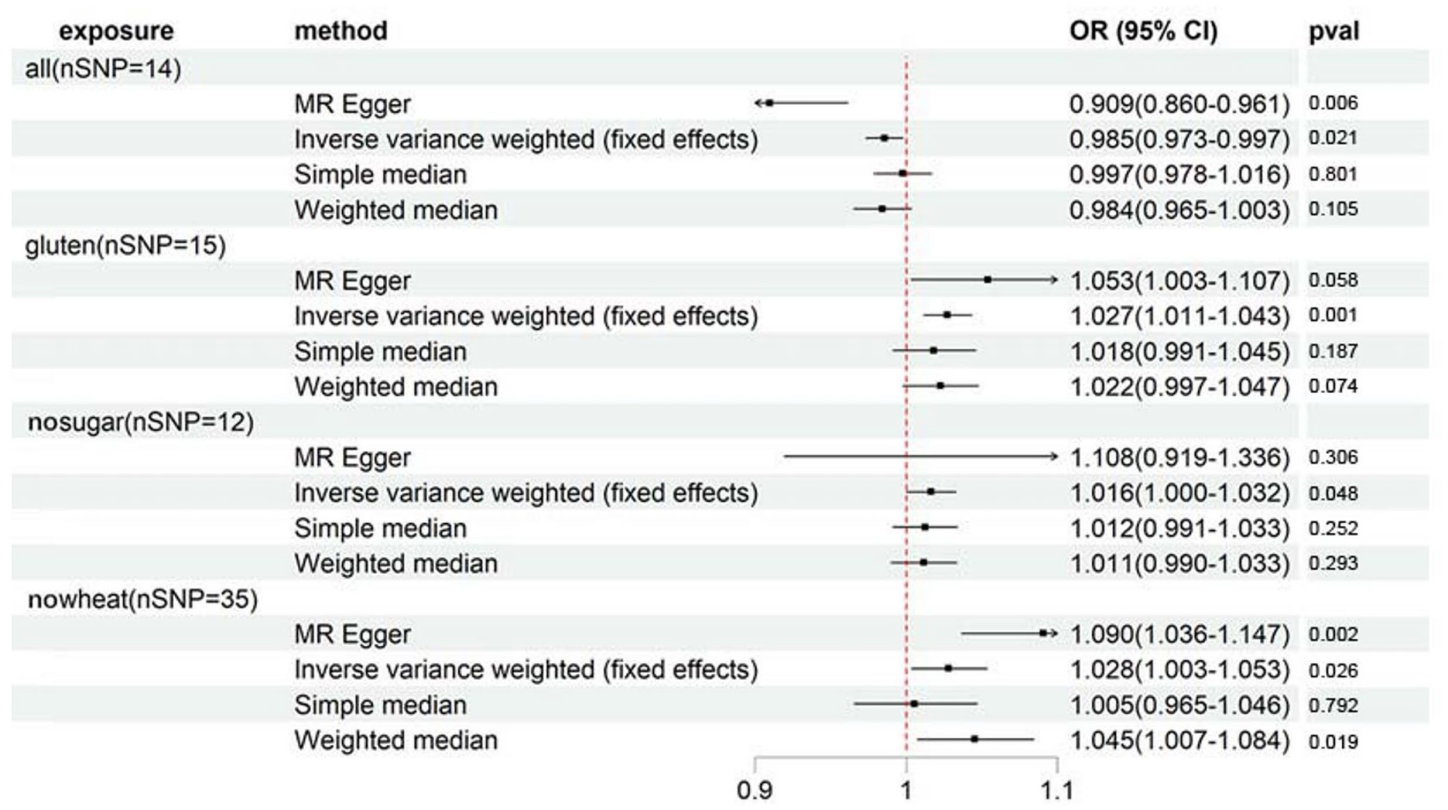
Forest plot showing results from Mendelian randomization study to assess associations between special diets and osteoporosis (diagnoses). nSNP, number of single-nucleotide polymorphisms; OR, odds ratio; CI, confidence interval; all, Eggs, dairy, wheat, sugar: I eat all of the above; gluten, Type of special diet followed: Gluten-free; nosugar, Never eat sugar: Sugar or foods/drinks containing sugar; nowheat, Never eat wheat: Wheat or products containing wheat.

In order to argue the study’s credibility, a secondary outcome - osteoporosis diagnoses - was incorporated as a validation measure. Despite the smaller number of cases (1976) for diagnosed osteoporosis compared to self-reported osteoporosis (number of cases: 7,547), the clearly diagnosed osteoporosis patients provide high reliability to the sample’s osteoporosis diagnosis. Supplementary Table S6 and Figure 3 indicate that although the 4 specialized diets are also linked to osteoporosis diagnoses, the strength of their association differs considerably from that of self-reported osteoporosis. Only GLUTEN exhibits a significant association with osteoporosis diagnoses. The sensitivity analyses were directionally consistent with the IVW method. MR-Egger intercept testing revealed horizontal pleiotropy in ALL and NOWHEAT (shown in Supplementary Table S7), indicating that there are unknown factors, other than ALL and NOWHEAT, that affect osteoporosis diagnoses. Consequently, we cannot establish a causal relationship between the two specialized diets and osteoporosis diagnoses. Finally, the association between GLUTEN, NOSUGAR, and osteoporosis diagnoses is visualized in Supplementary Figures S5, S6.

### 3.2 Special diet and bone mineral density

**Figure 4:**
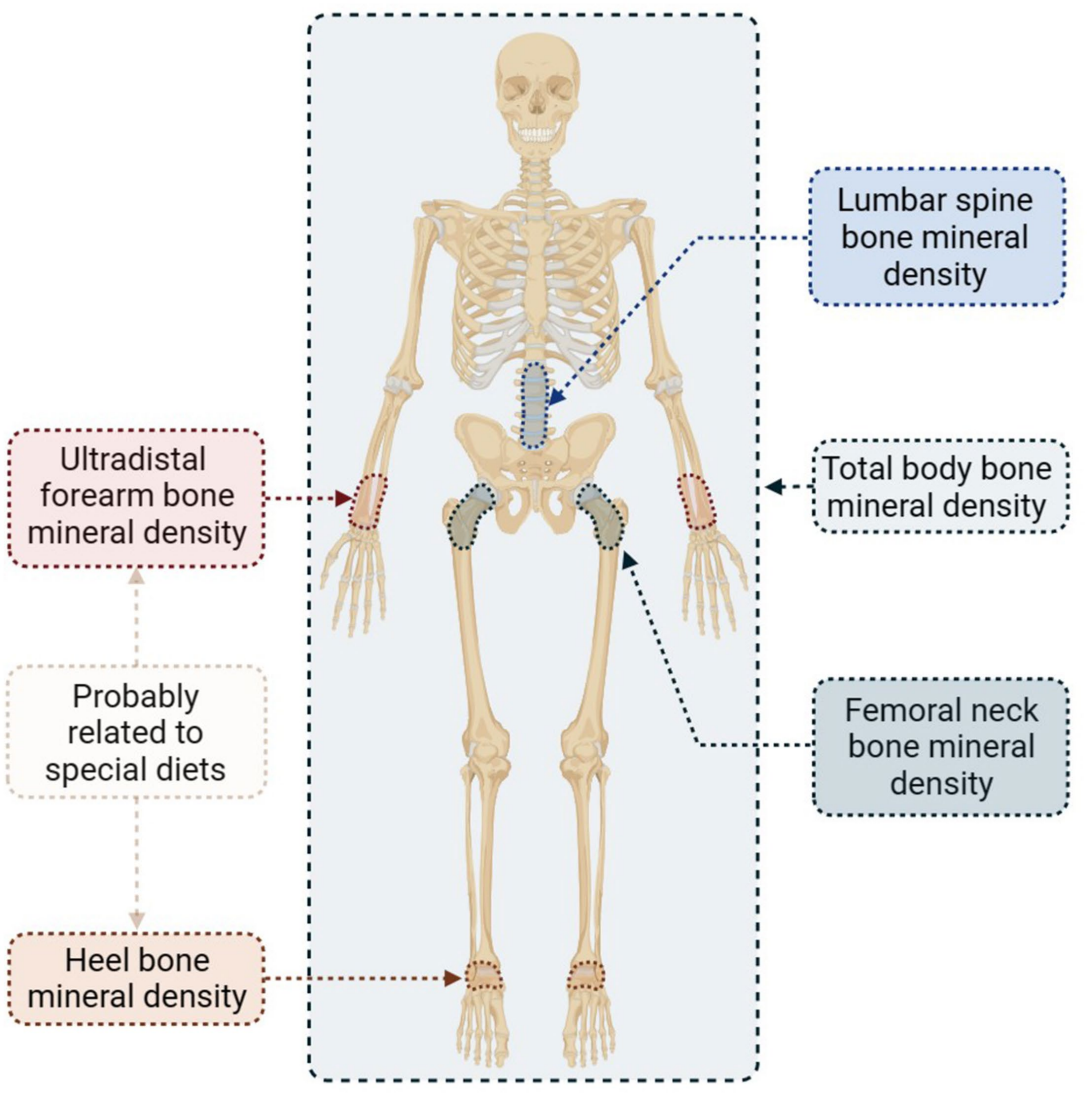
Bone mineral density selected sites and brief results.

The BMD serves as a valuable indicator for the diagnosis of osteoporosis. In this study, we analyzed five distinct BMD sites separately to determine the specific impact of specialized diets on osteoporosis. Figure 4 presents the selected sites along with concise information.

**Figure 5:**
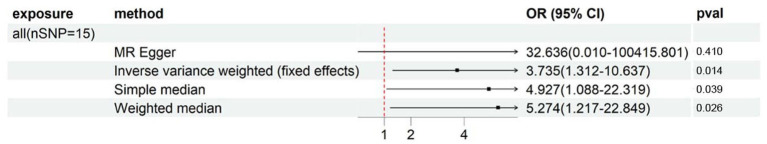
Forest plot showing results from Mendelian randomization study to assess associations between special diets and Ultradistal forearm BMD. nSNP, number of single-nucleotide polymorphisms; OR, odds ratio; CI, confidence interval; all, Eggs, dairy, wheat, sugar: I eat all of the above.

As evidenced by Supplementary Table S8 and Figure 5, there was a suggestive association between genetically predicted ALL (OR: 3.735, 95%CI: 1.312–10.637, *p* = 1.36E-02) and an increase in Ultradistal forearm BMD. Cochran’s *Q* test did not reveal any heterogeneity between the IVs of ALL and Ultradistal forearm BMD (shown in Supplementary Table S9). In conducting sensitivity analysis, the MR-Egger method yielded a point estimate that was consistent with that of the IVW method, and no horizontal pleiotropy was detected by the MR-Egger regression intercept (shown in Supplementary Table S9). Moreover, further global tests did not uncover any outliers (shown in Supplementary Table S9).

**Figure 6:**
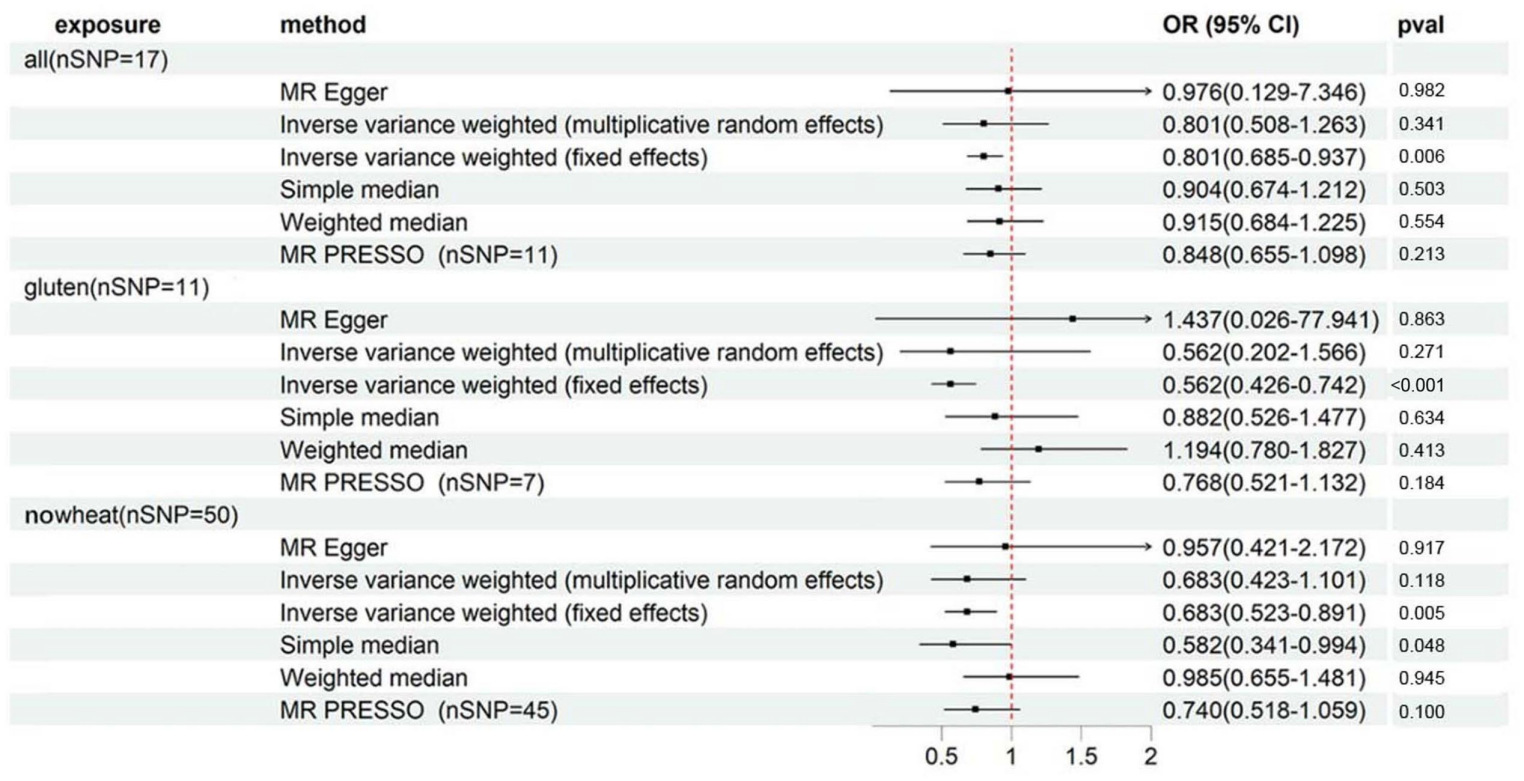
Forest plot showing results from Mendelian randomization study to assess associations between special diets and heel BMD. nSNP, number of single-nucleotide polymorphisms; OR, odds ratio; CI, confidence interval; all, Eggs, dairy, wheat, sugar: I eat all of the above; gluten, Type of special diet followed: Gluten-free; nowheat, Never eat wheat: Wheat or products containing wheat.

As per Supplementary Table S10 and Figure 6, we observed significant associations between heel BMD and ALL (OR: 0.801, 95%CI: 0.685–0.937, *p* = 5.59E-03), GLUTEN (OR: 0.562, 95%CI: 0.426–0.742, *p* = 4.69E-05), and NOWHEAT (OR: 0.683, 95%CI: 0.523–0.891, *p* = 5.07E-03) in the fixed-effects IVW method. However, heterogeneity was observed in all 3 special diets (PCochran’s *Q* < 0.05), indicating a possible bias in the fixed-effects IVW estimation (Supplementary Table S11). On the other hand, the random-effects IVW method did not show any association between ALL, GLUTEN, NOWHEAT, and heel BMD.

The MR-Egger sensitivity analysis indicated a contradictory point estimation of the association between GLUTEN and heel BMD compared to the main analysis (IVW method), but no horizontal pleiotropy was identified in the MR-Egger intercept test (Supplementary Table S11). The MR-PRESSO Global Test detected outliers in all 3 special diets (Supplementary Table S11), and after their exclusion, there was no association observed either.

There exists no correlation between the BMD of the residual 3 portions and specialized diets. Specific outcomes are exhibited in Supplementary Tables S12–S17.

## 4 Discussion

In this two-sample MR study, we explicated the correlation between 8 special diets and the incidence of osteoporosis or BMD. Also, we discovered a highly plausible relationship between GLUTEN and osteoporosis. Furthermore, we have also observed suggestive connections between ALL, NOSUGAR, and NOWHEAT, and osteoporosis.

Gluten-free diet (GFD) specifically eliminates gluten of diet, which is the chief protein constituent in wheat, rye, and barley (23). And it is also the primary cause of Celiac Disease (CD). Currently, the sole effective treatment for CD is a stringent lifelong GFD (24). As the implementation of a specialized GFD has been shown to be fraught with challenges and difficulties (23, 25), we deem it unlikely for individuals to undertake stringent GFD autonomously without medical guidance. Therefore, in this study, we believe that the crowd of GFD overlaps with the crowd of CD. And CD’s various complications, including osteoporosis.

The main cause for the association between CD and low BMD lies primarily in the characteristic malabsorption, resulting in deficiencies in vitamin D and intestinal calcium absorption. In addition, given the close hormonal interrelationship, deficiencies in calcium and vitamin D stimulate the secretion of parathormone (PTH), and hyperparathyroidism itself becomes another contributing factor, as elevated levels of PTH have been linked to bone mass loss through the activation of bone resorption (26). Combining with previous research (26–28), it is shown that GFD only exhibits significant improvement in BMD for CD patients initially presenting with secondary hyperparathyroidism, low serum calcium, and vitamin D. We can infer that gluten initiates the onset of CD in specific populations, and the occurrence of CD affects bone metabolism, leading to declining BMD and the manifestation of osteoporosis. According to this speculation, the elimination of the pathogenic source is the clearest approach, where lifelong abstinence from gluten should improve both CD and BMD simultaneously. Existing studies have also confirmed that while GFD cannot fully restore BMD to normal levels, it still exerts a positive effect on BMD improvement (29).

However, it is important to note that gluten merely acts as a “key” to trigger the onset of CD in specific populations, and the direct impact of gluten on the skeletal system has not been investigated. The establishment of steady-state in the skeletal system relies on the dynamic balance between bone formation and resorption (30). GFD blocks the entry of gluten to halt the destructive effects of CD on the skeletal system, reducing the outflow of bone mineral loss. However, if gluten is a component of bone formation, limiting its entry would impede new bone formation, making it challenging for BMD to fully recover to a normal state, which aligns with the aforementioned research findings (29). Similarly, the most significant correlation observed in this study is the promotive effect of GFD on the incidence of osteoporosis. Aligning with the superior strategy of GFD (gluten is the protein in wheat, so avoiding wheat will inevitably avoid gluten), the avoidance of wheat (NOWHEAT) exhibits the same outcome, while the consumption of wheat (ALL) in contrast demonstrates an inhibitory effect on the risk of osteoporosis. It is thus reasonable to believe that gluten merely triggers the onset of CD in specific populations without direct bone resorption effects, and it may even have a role in bone formation or promoting osteogenesis.

In accordance with a systematic review conducted in 2018, excessive consumption of sugar potentially carries the risk for osteoporosis (31). This hypothesis was initially confirmed in 2023 through a cohort study of 6,620 young individuals aged between 18 and 23 in Brazil, which demonstrated a correlation between frequent intake of sugary drinks and low lumbar BMD (32). While there are currently no further clinical studies examining the impact of dietary sugar intake on osteoporosis, the effect of sugar on BMD is worth considering. Diabetes, a metabolic condition characterized by hyperglycemia (33), is believed to affect bone remodeling and turnover, leading to defects in bone material quality. Apart from other organs, diabetes affects bone through impairment of glucose metabolism, toxic effects of glucose oxidative derivatives, and impairment of bone microvascular and muscle endocrine function (34). Recent animal experiments have shown that the “high glucose and high-fat diet”-induced ferroptosis in osteoblasts may be the primary cause of osteoporosis in diabetes through the activation of the METTL3/ASK1-p38 signaling pathway (35, 36). Combining the preliminary results of the relationship between sugar intake and osteoporosis obtained in this study, it is apparent that both excess and abstain from sugar consumption can lead to the onset of osteoporosis. Given the essential relationship between sugar and BMD, we can further hypothesize that since there needs to be a steady state between sugar and BMD, sugar may have an effect on BMD, and bone may also have an impact on glucose metabolism. This conjecture was confirmed in a 2020 study (37). Consequently, for the management of diabetic patients, commencing with the improvement of the bone environment may be a novel and promising idea.

Contrary to conventional wisdom, which posits the bone-strengthening benefits of milk and dairy products (38, 39), this study yielded unexpected results by indicating no correlation between the avoidance of dairy products and osteoporosis. This finding can be interpreted in a number of ways. For instance, dietary supplements are sufficient in compensating for the nutritional deficiency caused by the rejection of dairy products or dairy products themselves have no impact on bone health. While certain studies have suggested that dairy products play an irreplaceable role in bone health (40), meta-analyses of numerous large-scale clinical cohorts have demonstrated that the positive effects of consuming milk and dairy products on osteoporosis and fracture risk, as reported in cross-sectional and case–control studies, were not found in cohorts. Due to the heightened reliability of cohort studies over case–control studies, it is not the case that there exists a link between dairy products and osteoporosis and fractures (41, 42). This discovery merits a comprehensive investigation into the relationship between dairy products and bone health.

The impact of ceasing egg intake on osteoporosis was not observed in this study, despite the fact that eggs constitute a primary source of daily protein intake. Nonetheless, in contrast to the equivocal role of dairy products, research has suggested that eggs may have a beneficial effect on bone health. A cross-sectional study found a favorable association between whole egg consumption and bone mineral density (43). Additionally, an oral peptide derived from egg yolk is believed to promote bone repair in mice (44), and the duck egg white-derived peptide VSEE (Val-ser-glu-glu) has been demonstrated to enhance bone repair through the wnt/β-catenin signaling pathway, as well as regulate bone and lipid metabolism through gut microbiota (45). Furthermore, a retrospective study in Spain demonstrated that eggs also regulate osteoporosis induced by vitamin D deficiency (46). In summary, while eggs may impact bone health, the mechanisms underlying their effects are multifaceted, and abstaining from egg consumption is not detrimental to bone health.

Some scholars contend that the vegetarian diet, which engender controversy, can lead to a deficiency of calcium and vitamin D, potentially resulting in adverse impacts on bone mineral density (47, 48). A cross-sectional study conducted in Poland has suggested that vegetarian diets may be associated with an increased risk of nutrient deficiencies, as well as decreased bone mineral content and height, although the nutrient deficiency was insignificant among vegetarians (49). However, recent studies have presented differing perspectives. One three-year retrospective survey found no link between vegetarianism and BMD, except for women aged 40–55 (50). Additionally, despite the distinct acid–base profiles of vegetarians and omnivores, no association was found between bone health and the range from alkaline to low acid load (51). Vegetarian diets typically contain many other micronutrients vital for bone health, including vitamins C and K, carotenoids, potassium, and magnesium, among others (52). Thus, taking into account the conclusions of MR, this study proposes that there may be no association between a vegetarian diet and osteoporosis.

The study on the low-calorie diet is the most equivocal among investigations. Not only was there no correlation with osteoporosis in this study, but the inquiry into the relationship between calorie restriction (CR) and bone health has not been renewed for a prolonged duration. In 2014, a study alluded to the unfavorable effects of CR on trabecular and cortical bone (53), but in the updated systematic review of 2019, it was demonstrated that CR appears to decrease BMD, while it does not appear to impact bone integrity (54).

One of the merits of this study is the exploration of the relationship between osteoporosis and multiple special diets through MR analysis, which renders it the most extensive study to characterize the correlations between diet and osteoporosis. Moreover, the MR design itself remains impervious to residual clutter. By employing various MR methods to eliminate SNPs associated with multiple special diets, we have eliminated the effects of potential pleiotropy on the results, thus making it less likely for horizontal pleiotropy to disrupt our findings. Furthermore, the genetic variants in special diets and osteoporosis are derived from summary-level data from GWAS with large sample sizes, which is another advantage of this study.

However, this study also has some limitations. Firstly, although we have taken control measures, IVs may still have unmeasurable confounding and has affected the outcome as a result. Secondly, many IVs rely on monotonicity conditions, which means estimating the IVs effect under monotonicity often involves an unrecognized subgroup in the study population. Using subgroup results to guide decision-making is not an ideal method, and if more information is provided, the correlation between the subgroup effects will significantly increase (55). Our IVs are genetic variants identified from the United Kingdom Biobank, and we only know the size of the subgroup of IV origins, while the specific characteristics of this subgroup remain unknown to us. Additionally, it is difficult to quantify the sensitivity of effect estimation to monotonicity bias. Therefore, our analysis may violate monotonicity, which may render our results unsuitable for an extension to a larger population. Thirdly, while bone mineral density is the gold standard for the diagnosis of osteoporosis, only the Ultradistal forearm BMD suggested an association with special diets in this study. However, this index is usually not used in the clinical diagnosis of osteoporosis. Given that special diets are associated with osteoporosis, but almost not associated with its diagnostic criteria, we believe that a larger database should be used to verify the results. Finally, although the MR method can provide associational estimates, the results reported here cannot automatically be assumed to be causal because there is considerable room for other explanations. For example, GFD is not only limited to gluten but also other nutrients because of the elimination of certain grains. Some people use pre-prepared processed foods that are GF, which may have higher levels of sodium. Others might consume more GF starches such as potatoes and rice, therefore, overall nutrients from GFD could be quite variable and if not executed appropriately such diets could lead to nutrient deficiency beyond GF. Many people use GF diets even though they do not have CD either because they suspect they have gluten allergy or they think GF diets are healthier due to perhaps low carbohydrate levels. But we think this study, it is still one of the results with higher credibility and has considerable in-depth value.

Therefore, our findings should be interpreted with caution, and well-designed prospective studies are still needed to confirm our results in the future.

## 5 Conclusion

This work characterizes the correlations between genetically predicted special diets and osteoporosis. Our study preliminarily showed that simultaneous intake of eggs, dairy, wheat, and sugar could significantly reduce the risk of osteoporosis; and the abstain of gluten, wheat, and sugar could raise the risk of osteoporosis. Moreover, based on the results, a hypothesis was put forward that apart from CD, GFD for treating CD also caused osteoporosis. Our results should be interpreted carefully, and well-designed prospective studies are still needed to confirm our findings in the future.

## Data availability statement

The original contributions presented in the study are included in the article/Supplementary material, further inquiries can be directed to the corresponding author.

## Ethics statement

Ethical review and approval was not required for the study, in accordance with the local legislation and institutional requirements.

## Author contributions

CZ: Conceptualization, Methodology, Software, Investigation, Resources, Data curation, Writing – original draft, Project administration. LY: Validation, Visualization, Writing – review & editing. CL: Validation, Formal analysis, Writing - original draft. HM: Formal analysis, Writing – original draft. FY: Methodology, Validation, Funding acquisition, Writing – review & editing. LC: Conceptualization, Writing – review & editing, Supervision.

## Footnotes

^1^ https://gwas.mrcieu.ac.uk/
