# Supplementary material for: Associations between special diet and incidence risk of osteoporosis: a Mendelian randomization study
Source: Front Public Health. 2024 May 30;12:1364735. doi: 10.3389/fpubh.2024.1364735 (PMC11171419; doi:10.3389/fpubh.2024.1364735)
Supplement: Supplementary file 1 [file Data_Sheet_1.docx]

| A  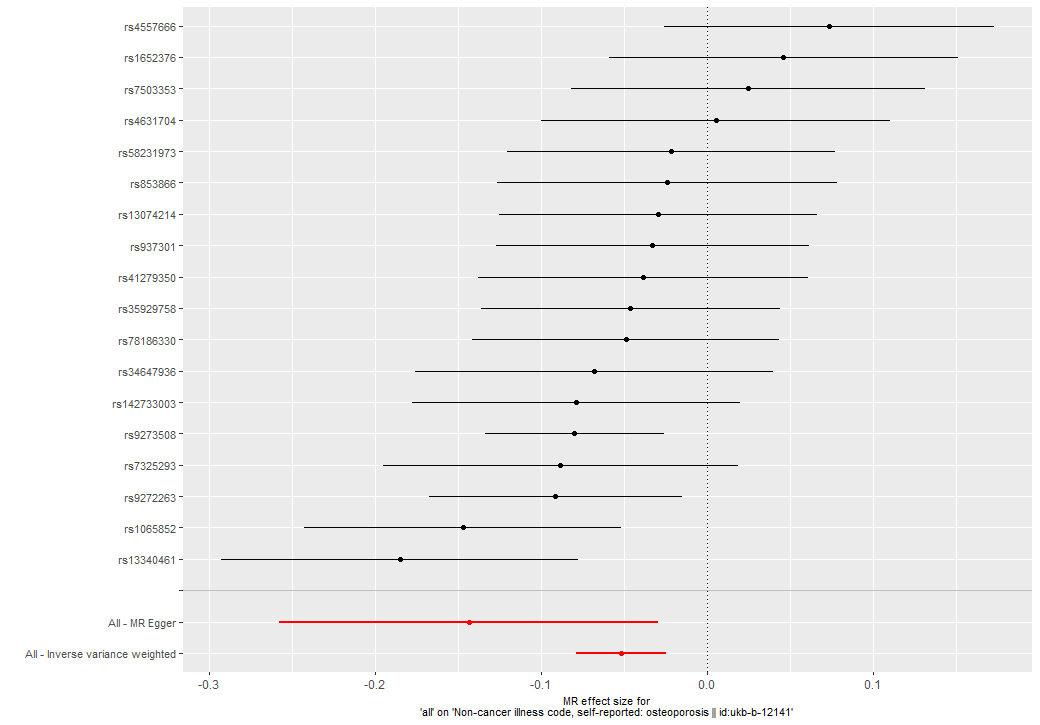 | B  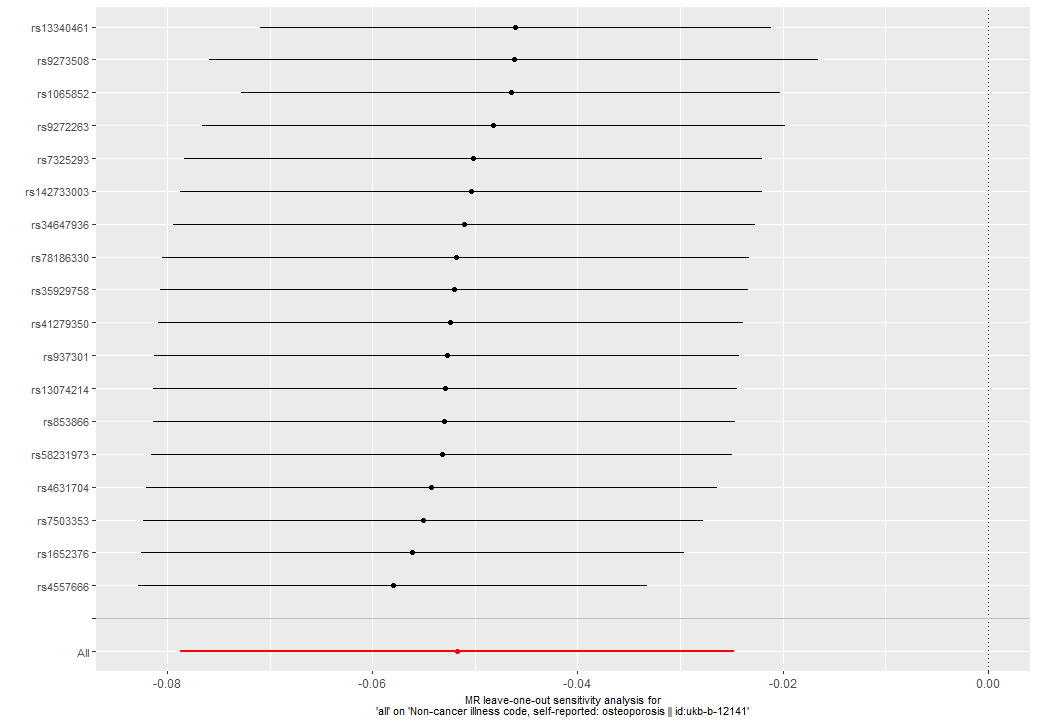 |
| --- | --- |
| C  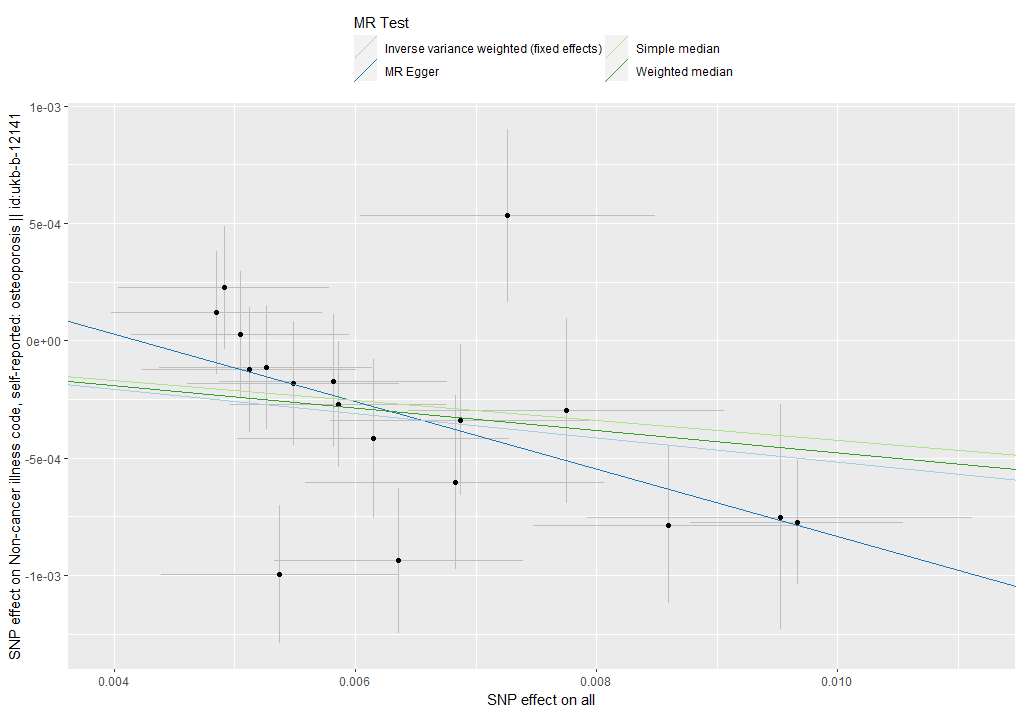 | D  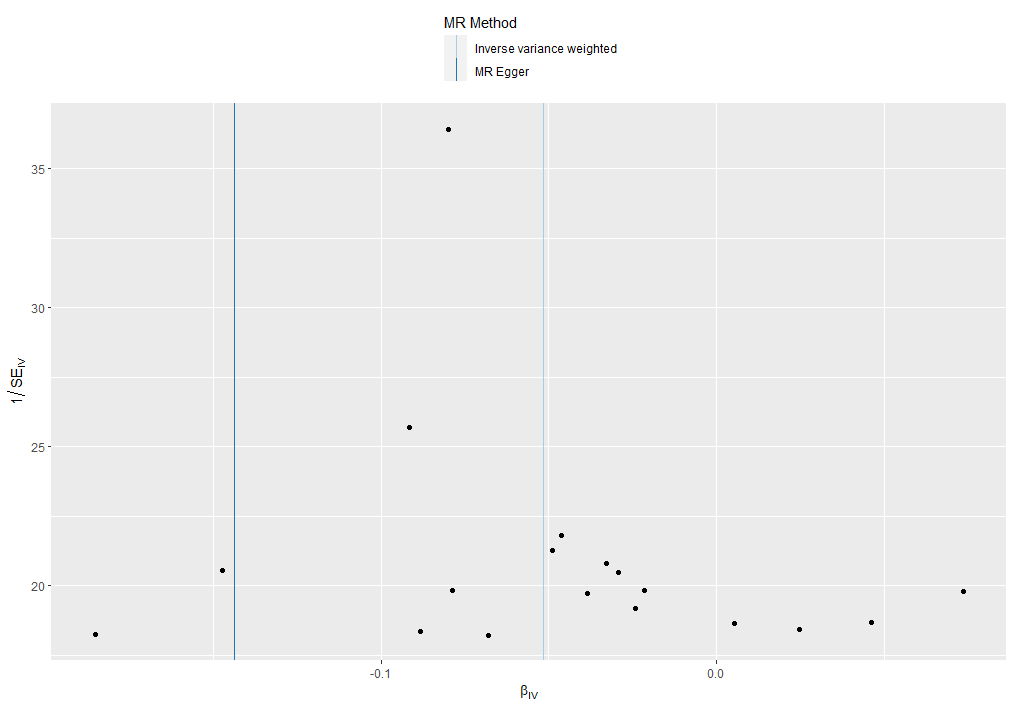 |

Supplementary Figure 1. The forest plot (A), leave-one-out plot (B), scatter plot (C) and funnel plot (D) for the association of “Eggs, dairy, wheat, sugar: I eat all of the above” and osteoporosis(self-reported) in Mendelian randomization analysis.

| A  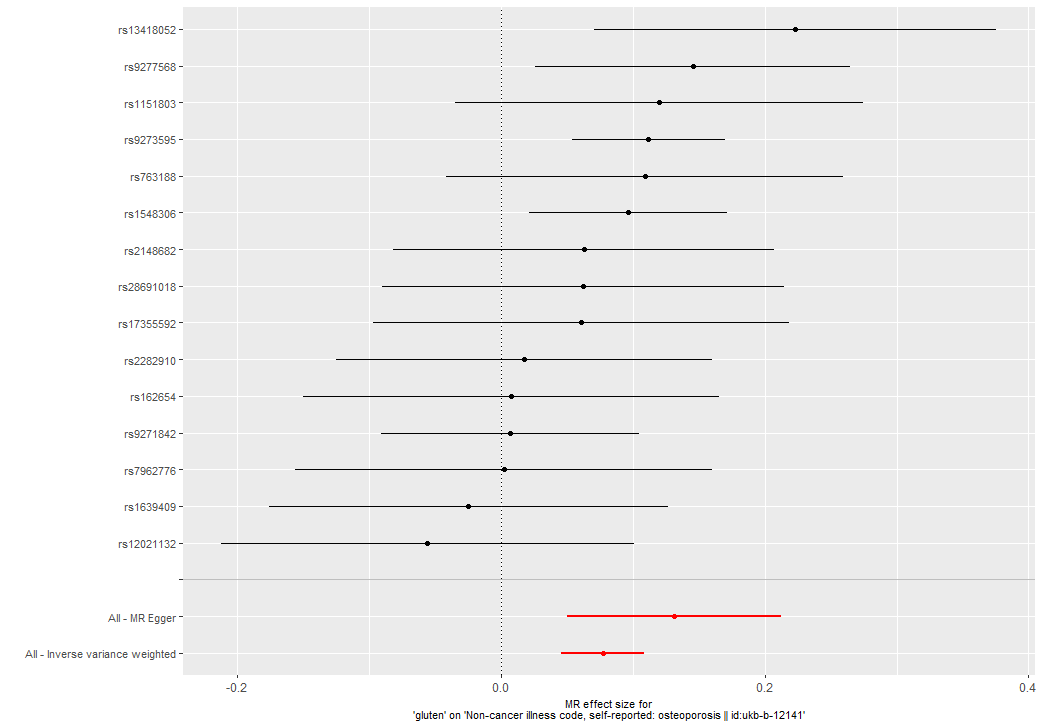 | B  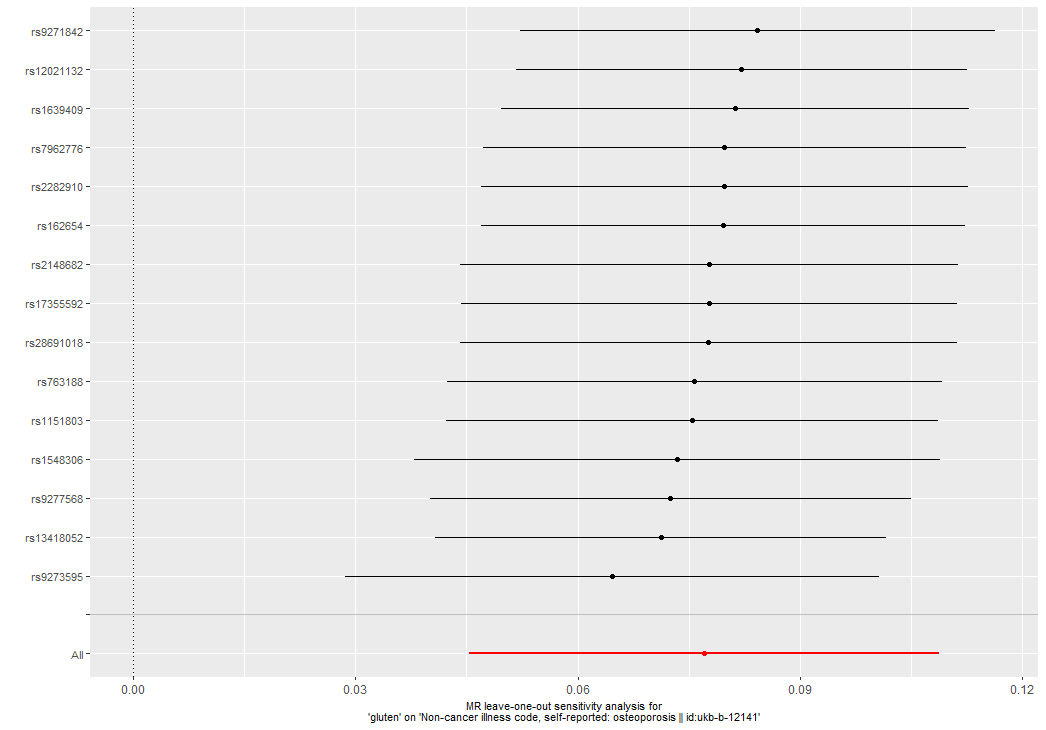 |
| --- | --- |
| C  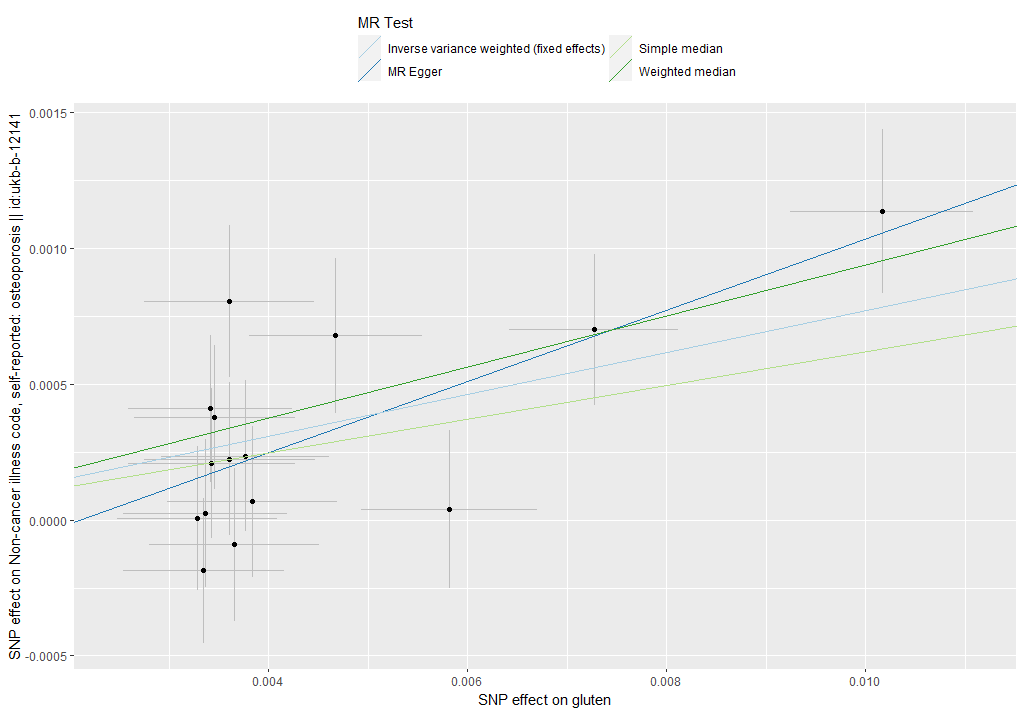 | D  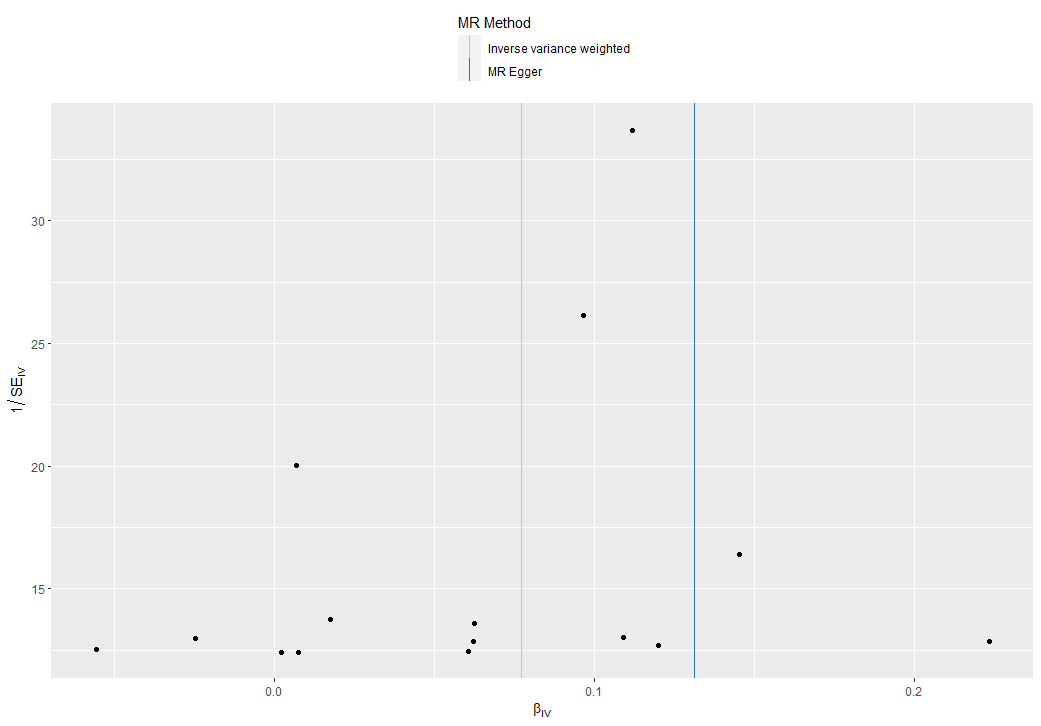 |

Supplementary Figure 2. The forest plot (A), leave-one-out plot (B), scatter plot (C) and funnel plot (D) for the association of “Type of special diet followed: Gluten-free” and osteoporosis(self-reported) in Mendelian randomization analysis.

| A  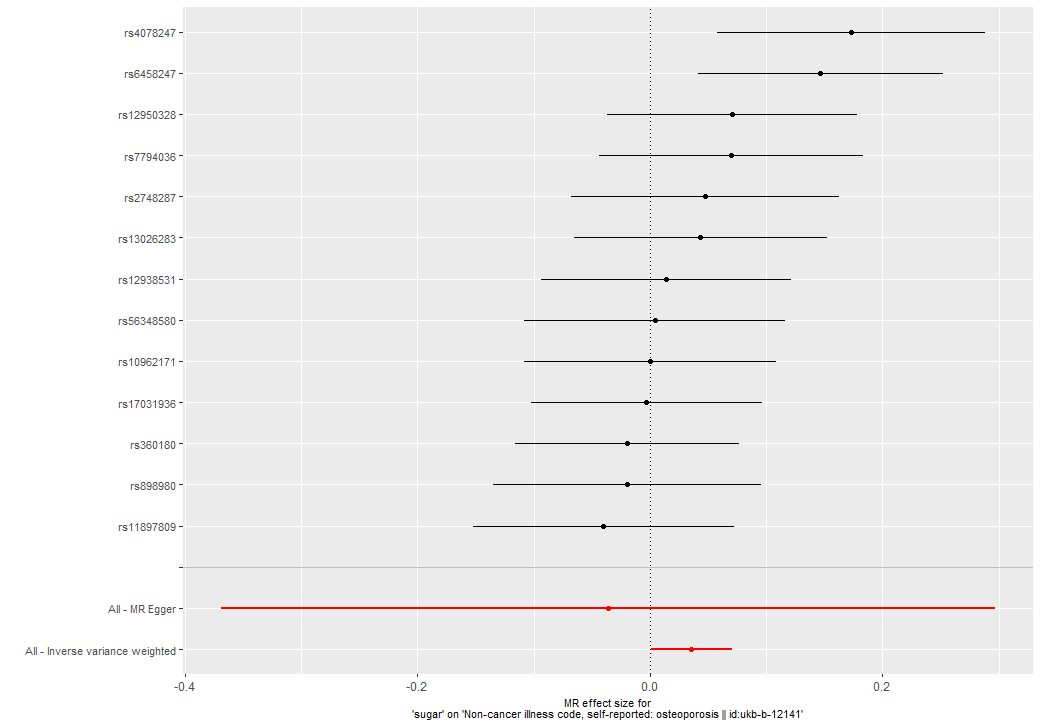 | B  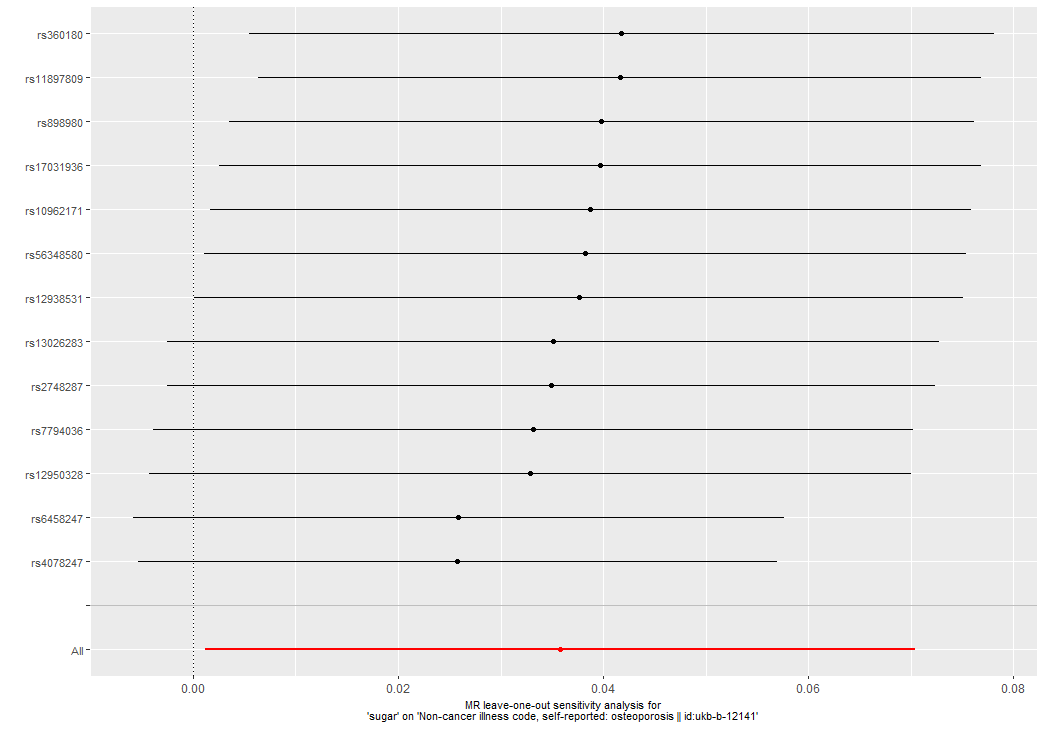 |
| --- | --- |
| C  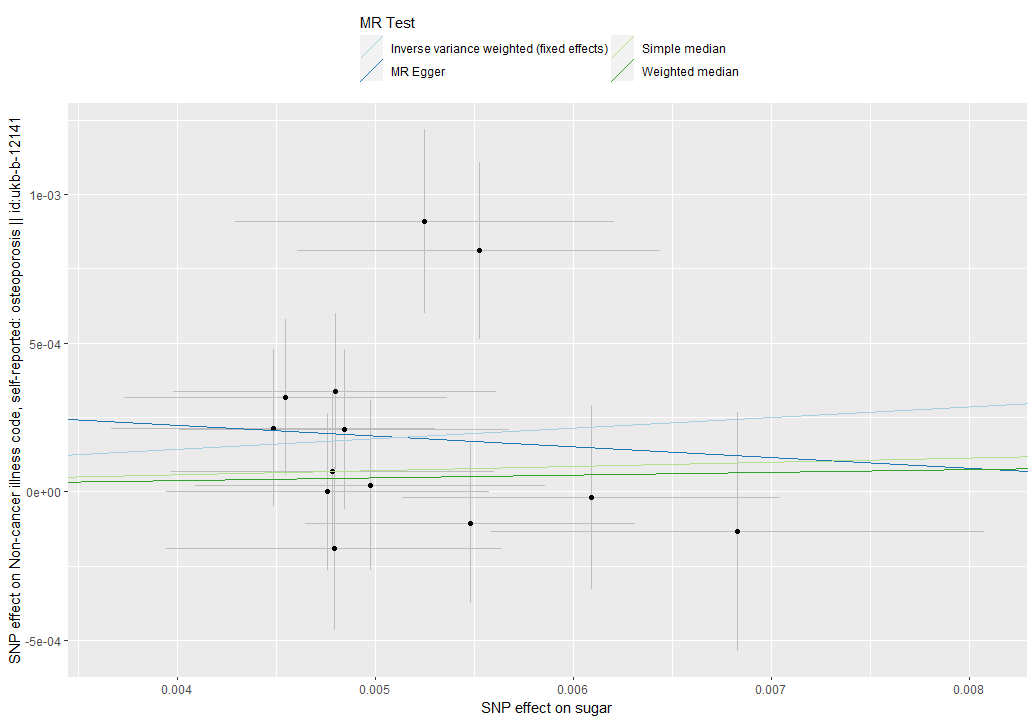 | D  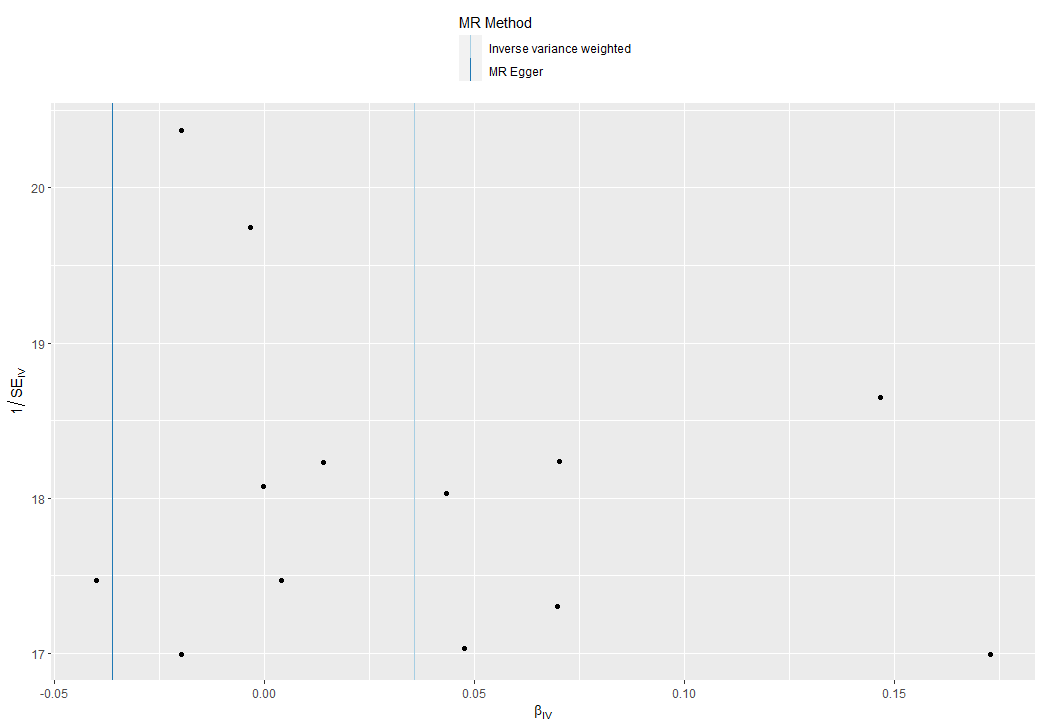 |

Supplementary Figure 3. The forest plot (A), leave-one-out plot (B), scatter plot (C) and funnel plot (D) for the association of “Never eat sugar: Sugar or foods/drinks containing sugar” and osteoporosis(self-reported) in Mendelian randomization analysis.

| A  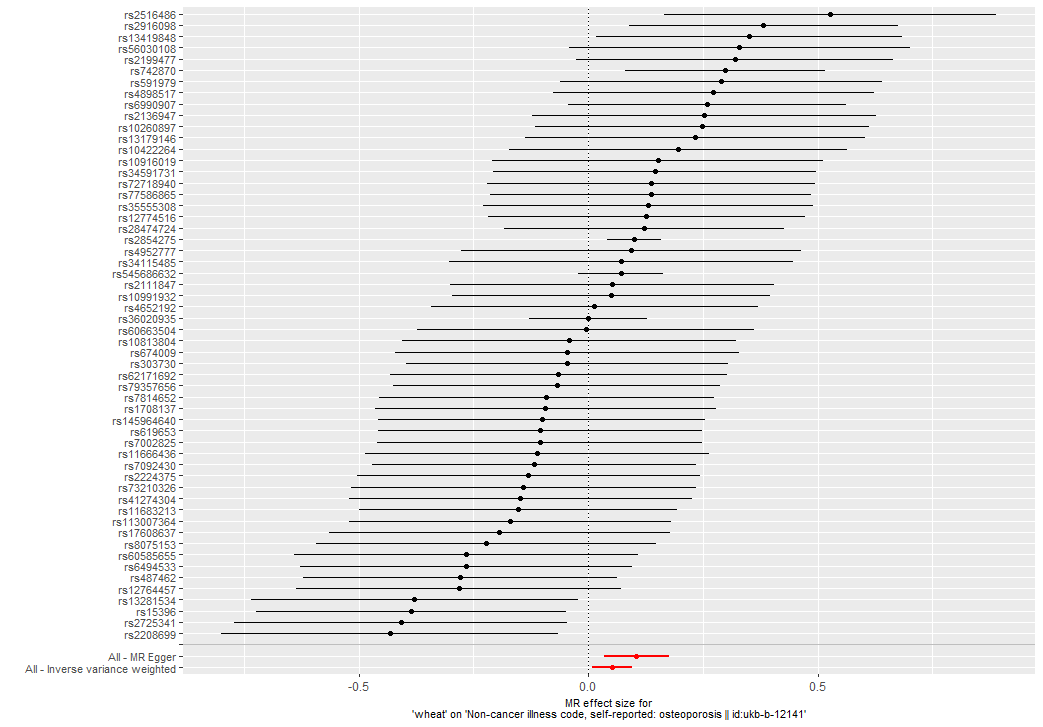 | B  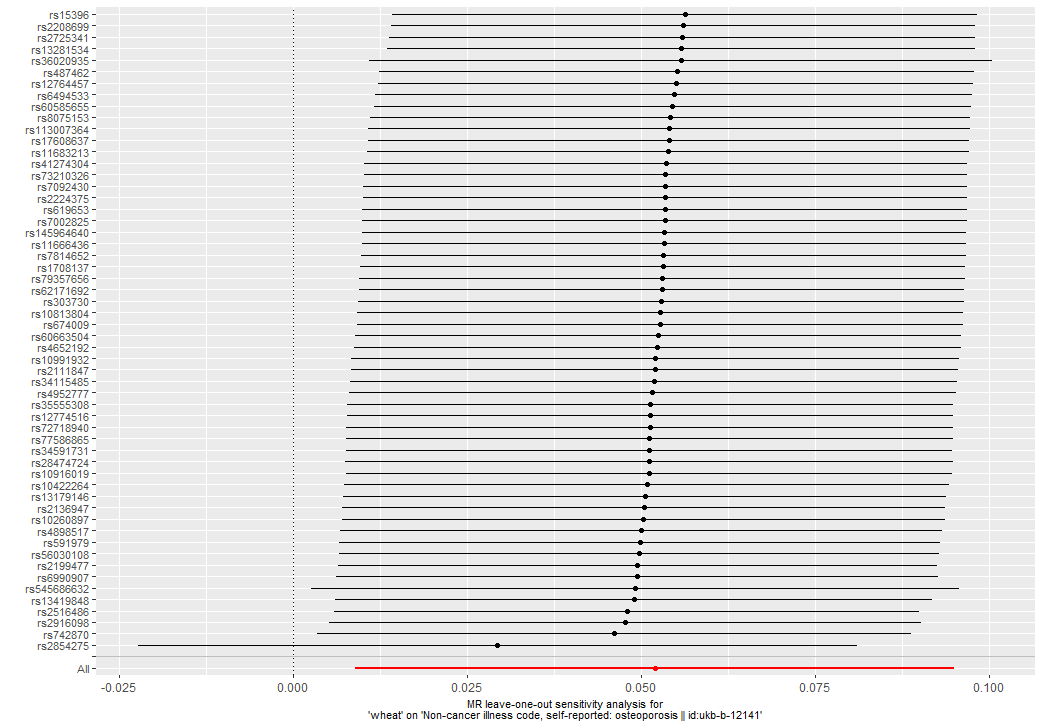 |
| --- | --- |
| C  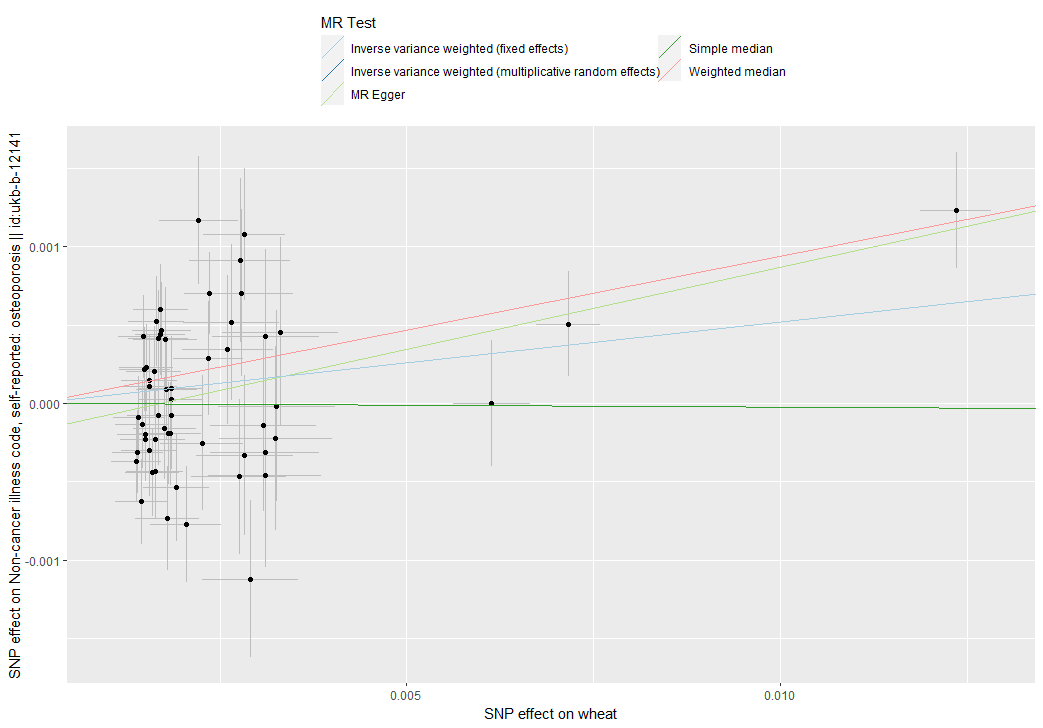 | D  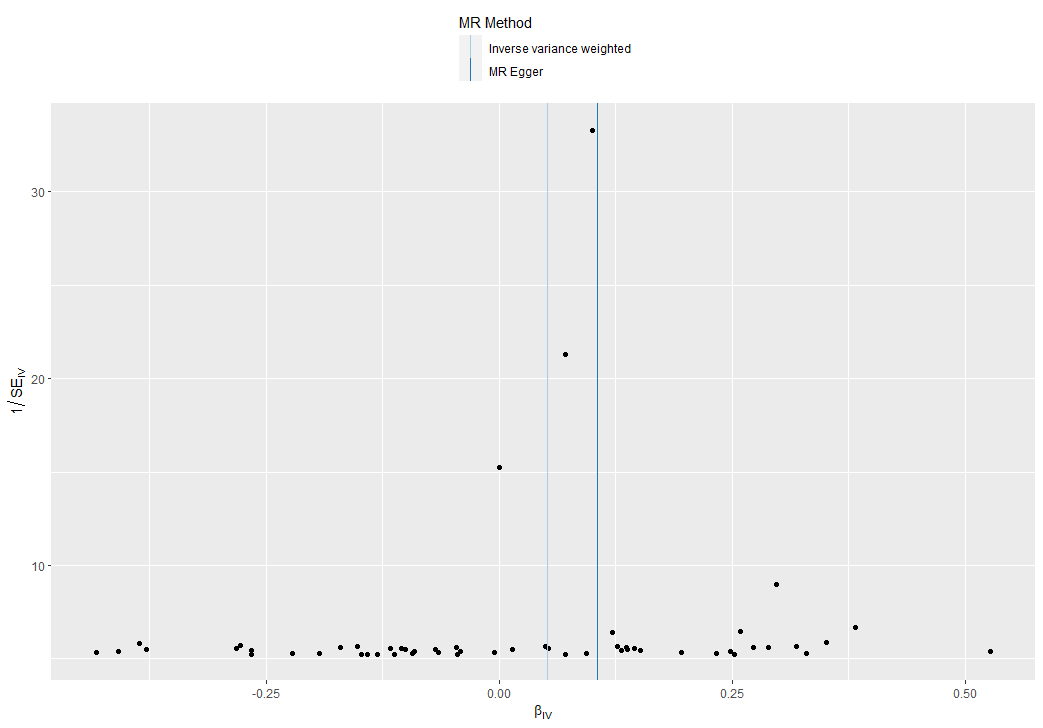 |

Supplementary Figure 4. The forest plot (A), leave-one-out plot (B), scatter plot (C) and funnel plot (D) for the association of “Never eat wheat: Wheat or products containing wheat” and osteoporosis(self-reported) in Mendelian randomization analysis.

| A  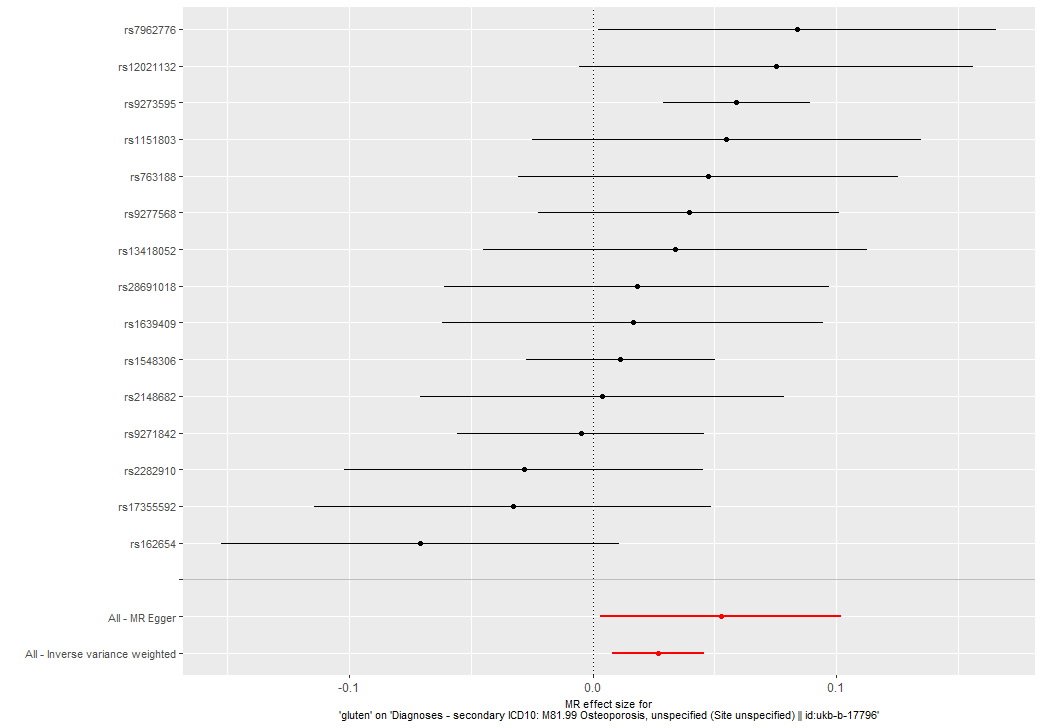 | B  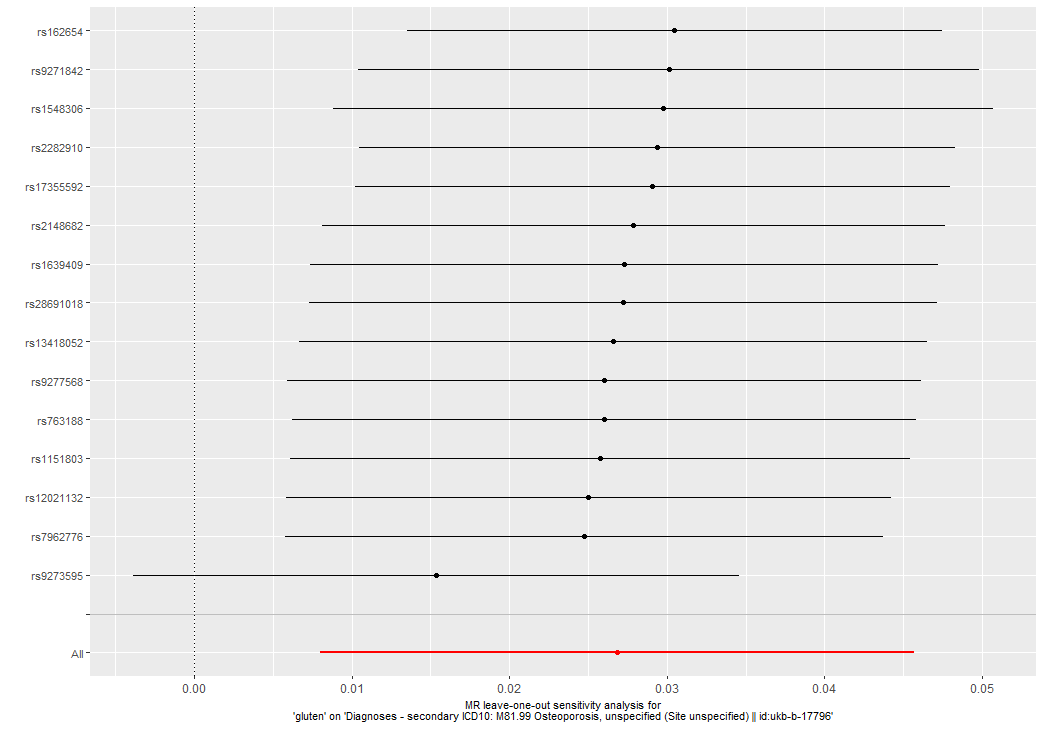 |
| --- | --- |
| C  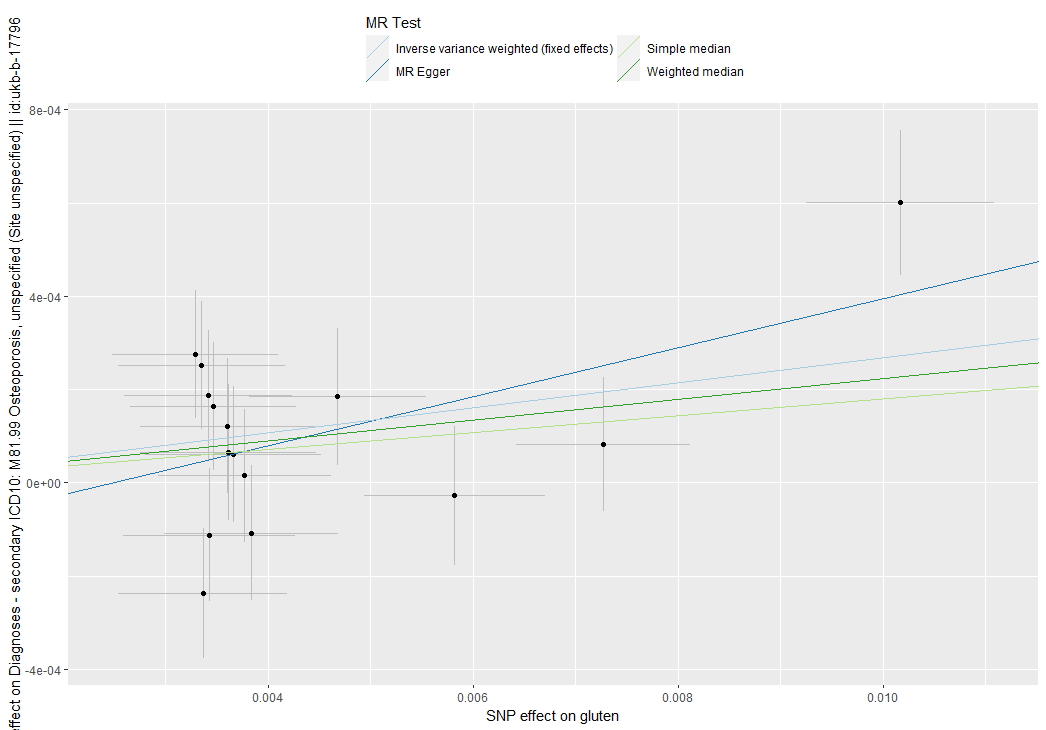 | D  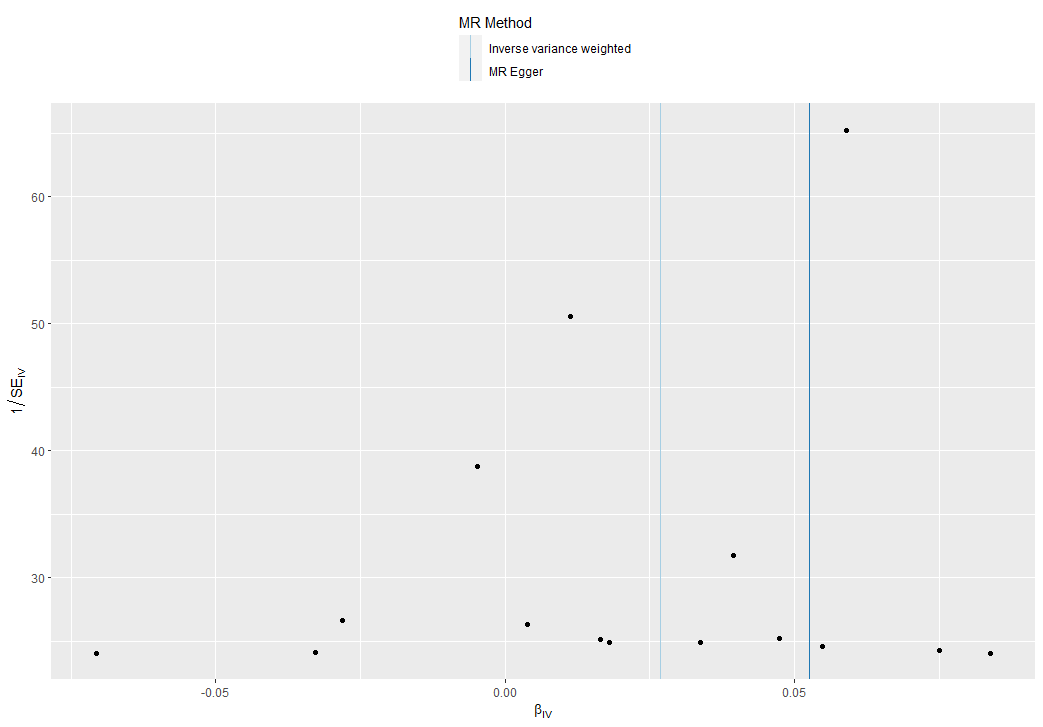 |

Supplementary Figure 5. The forest plot (A), leave-one-out plot (B), scatter plot (C) and funnel plot (D) for the association of “Type of special diet followed: Gluten-free” and osteoporosis(diagnoses) in Mendelian randomization analysis.

| A  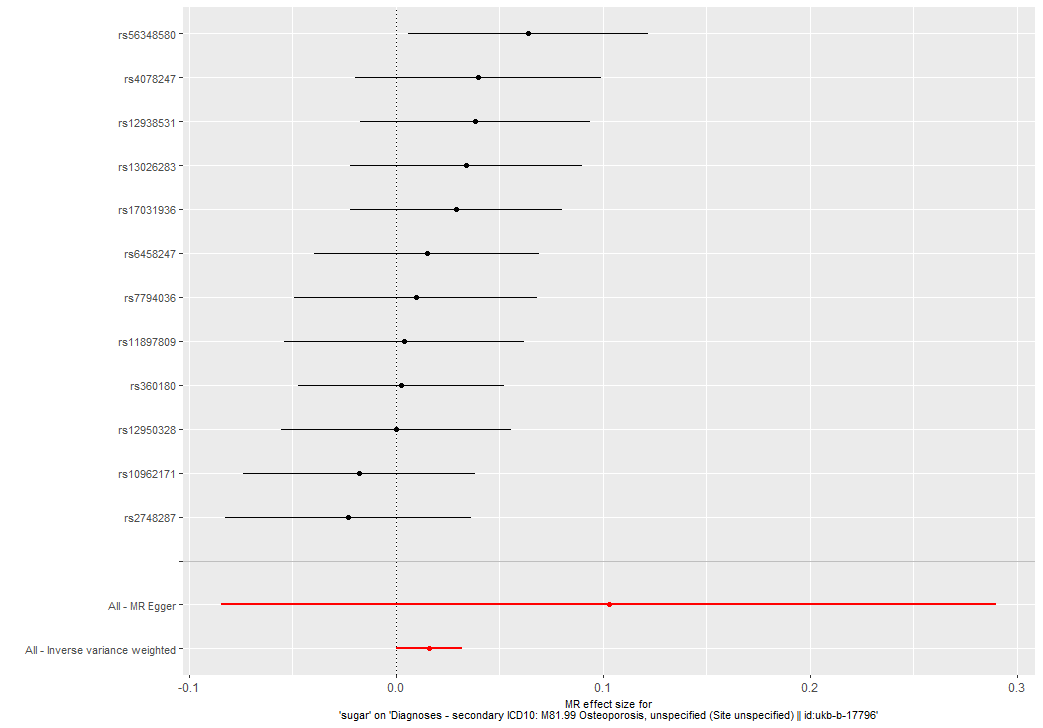 | B  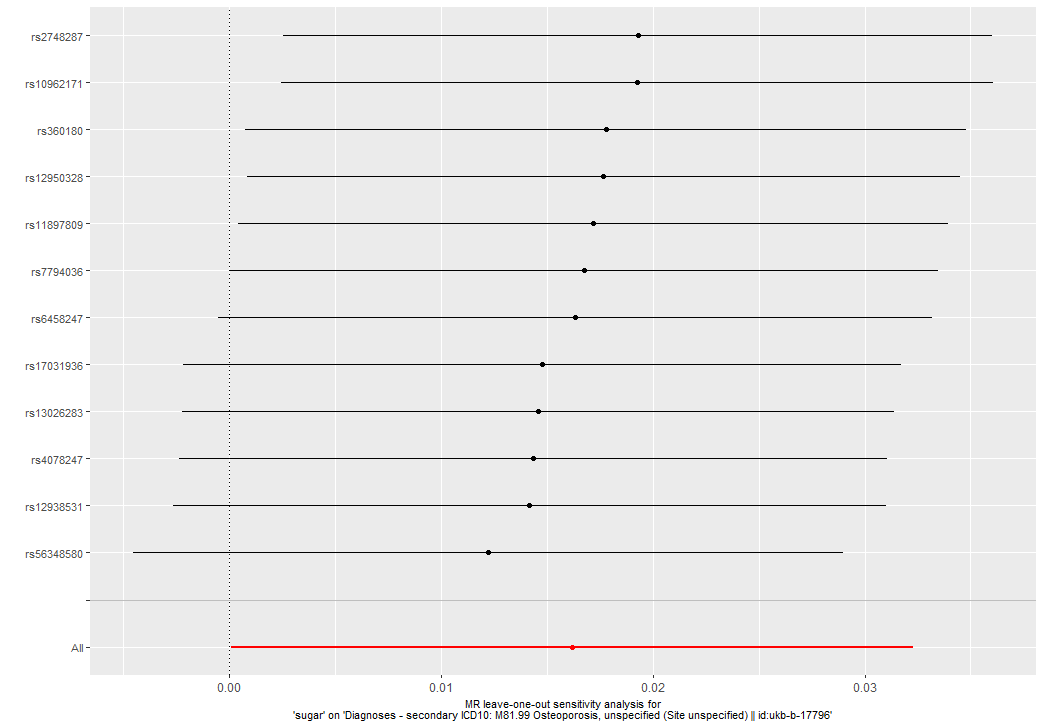 |
| --- | --- |
| C  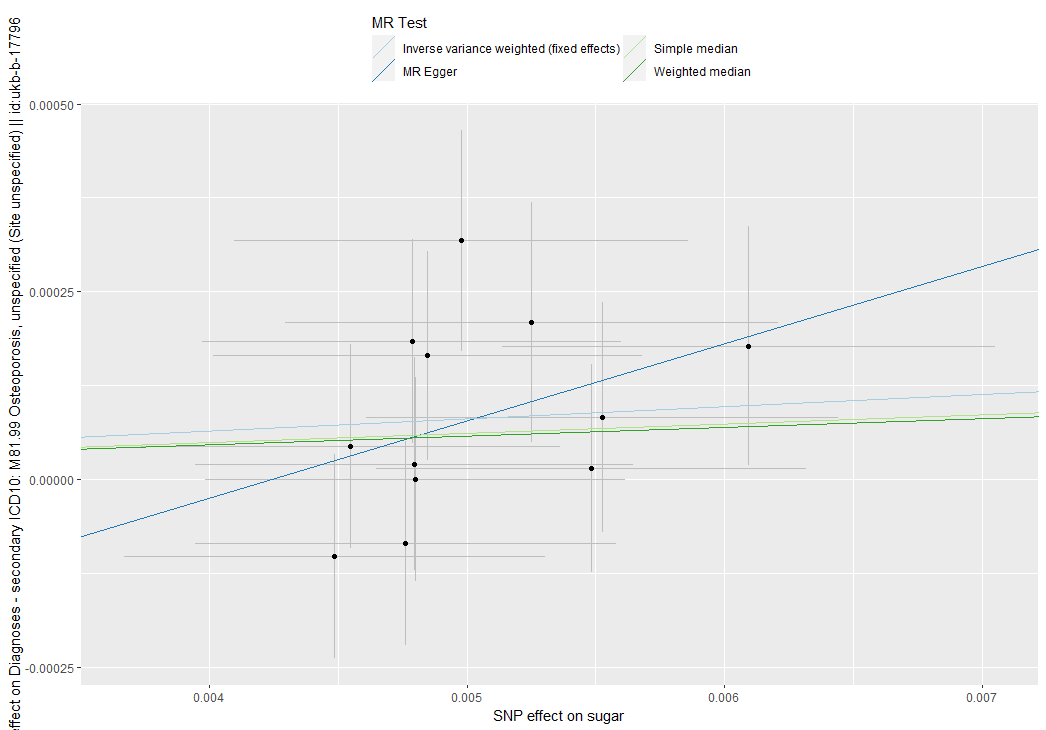 | D  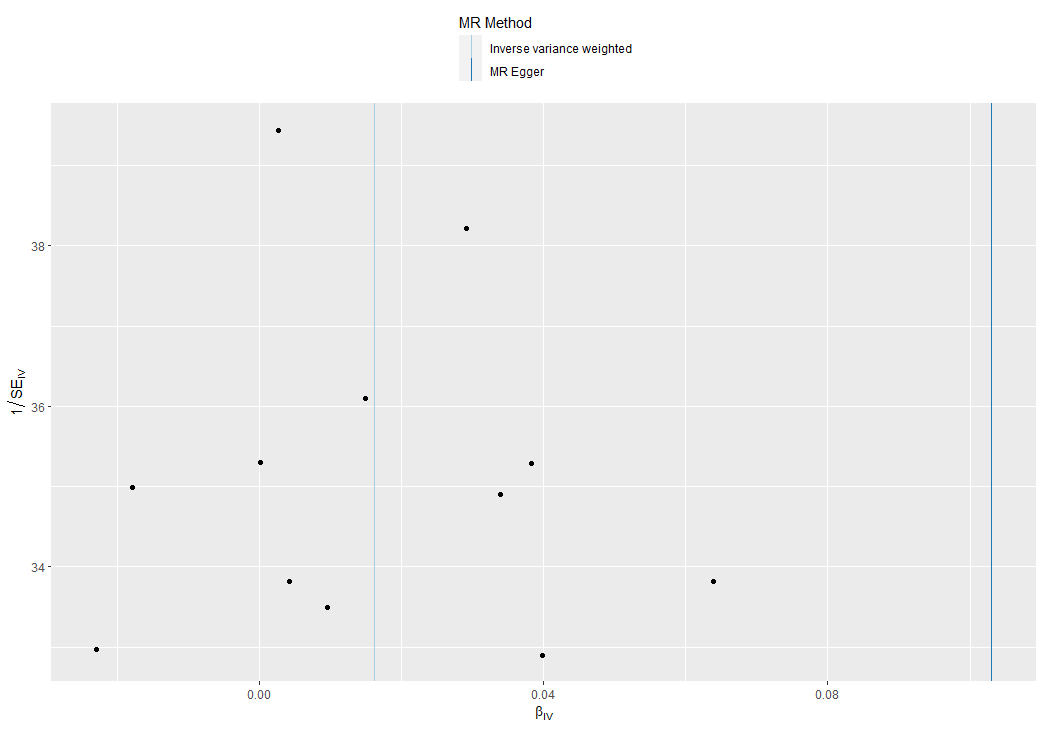 |

Supplementary Figure 6. The forest plot (A), leave-one-out plot (B), scatter plot (C) and funnel plot (D) for the association of “Never eat sugar: Sugar or foods/drinks containing sugar” and osteoporosis(diagnoses) in Mendelian randomization analysis.

Supplementary Table 1. Characteristics of pleiotropic SNPs associated with multiple special diets.

| SNPs | Exposure |
| --- | --- |
| rs11678980 | all, nosugar |
| rs9825208 | all, nosugar |
| rs35225200 | all, nosugar |
| rs3756362 | all, nosugar |
| rs6902789 | all, nosugar |
| rs10760201 | all, nosugar |
| rs7903146 | all, nosugar |
| rs28529403 | all, nosugar |
| rs838133 | all, nosugar |
| rs62033406 | calo, noegg |
| rs35011991 | nodairy, noegg |

all = Eggs, dairy, wheat, sugar: I eat all of the above

calo = Type of special diet followed: Low calorie

nodairy = Never eat dairy: Dairy or products containing dairy

noegg = Never eat eggs: Eggs or foods containing eggs

nosugar = Never eat sugar: Sugar or foods/drinks containing sugar

Supplementary Table 2. The summary of information regarding studies and datasets.

| **Data** | **Sample Size** | ***p*-Value** | **Ancestry** | **Access Link** |
| --- | --- | --- | --- | --- |
| **Exposure** |  |  |  |  |
| Never eat eggs: Eggs or foods containing eggs | 461,046 | 5*10^-5^ | European | https://gwas.mrcieu.ac.uk/datasets/ukb-b-17455/ |
| Never eat dairy: Dairy or products containing dairy | 461,046 | 5*10^-5^ | European | https://gwas.mrcieu.ac.uk/datasets/ukb-b-18909/ |
| Never eat wheat: Wheat or products containing wheat | 461,046 | 5*10^-5^ | European | https://gwas.mrcieu.ac.uk/datasets/ukb-b-3599/ |
| Never eat sugar: Sugar or foods/drinks containing sugar | 461,046 | 5*10^-8^ | European | https://gwas.mrcieu.ac.uk/datasets/ukb-b-5495/ |
| Eggs, dairy, wheat, sugar: I eat all of the above | 461,046 | 5*10^-8^ | European | https://gwas.mrcieu.ac.uk/datasets/ukb-b-2393/ |
| Type of special diet followed: Gluten-free | 64,949 | 5*10^-5^ | European | https://gwas.mrcieu.ac.uk/datasets/ukb-b-11189/ |
| Type of special diet followed: Vegetarian | 64,949 | 5*10^-5^ | European | https://gwas.mrcieu.ac.uk/datasets/ukb-b-11679/ |
| Type of special diet followed: Low calorie | 64,949 | 5*10^-5^ | European | https://gwas.mrcieu.ac.uk/datasets/ukb-b-15768/ |
| **Outcome** |  |  |  |  |
| Non-cancer illness code, self-reported: osteoporosis | 462,933 |  | European | https://gwas.mrcieu.ac.uk/datasets/ukb-b-12141/ |
| Diagnoses - secondary ICD10: M81.99 Osteoporosis, unspecified (Site unspecified) | 463,010 |  | European | https://gwas.mrcieu.ac.uk/datasets/ukb-b-17796/ |
| Heel bone mineral density | 426,824 |  | European | https://gwas.mrcieu.ac.uk/datasets/ebi-a-GCST006979/ |
| Total body bone mineral density | 56,284 |  | European | https://gwas.mrcieu.ac.uk/datasets/ebi-a-GCST005348/ |
| Femoral neck bone mineral density | 32,735 |  | Mixed | https://gwas.mrcieu.ac.uk/datasets/ieu-a-980/ |
| Lumbar spine bone mineral density | 28,498 |  | Mixed | https://gwas.mrcieu.ac.uk/datasets/ieu-a-982/ |
| Ultradistal forearm bone mineral density | 21,907 |  | European | https://gwas.mrcieu.ac.uk/datasets/ebi-a-GCST90013422/ |

Supplementary Table 3. Summary information for SNPs that were used as genetic instruments for Mendelian randomization analyses of special diets.

| **Exposure** | **SNPs** | **EA** | **NEA** | **EAF** | **BETA** | **SE** | **P** | **R2** | **F** | **N** |
| --- | --- | --- | --- | --- | --- | --- | --- | --- | --- | --- |
| all | rs4631704 | T | C | 0.609094 | 0.005048 | 0.0009 | 2.1E-08 | 6.81946E-05 | 31.44288 | 461046 |
| all | rs853866 | A | T | 0.413056 | 0.00512 | 0.000889 | 8.4E-09 | 7.19718E-05 | 33.18453 | 461046 |
| all | rs13074214 | A | G | 0.306367 | 0.005822 | 0.000946 | 7.5E-10 | 8.21867E-05 | 37.8948 | 461046 |
| all | rs142733003 | A | G | 0.081631 | 0.009521 | 0.001596 | 2.4E-09 | 7.71845E-05 | 35.58818 | 461046 |
| all | rs9272263 | C | G | 0.195074 | -0.00859 | 0.001112 | 1.1E-14 | 0.000129529 | 59.72647 | 461046 |
| all | rs9273508 | A | G | 0.428745 | -0.00966 | 0.000882 | 6.5E-28 | 0.000260108 | 119.9523 | 461046 |
| all | rs13340461 | T | C | 0.274767 | 0.005375 | 0.00098 | 4.1E-08 | 6.52997E-05 | 30.10799 | 461046 |
| all | rs4557666 | A | T | 0.152819 | 0.007264 | 0.001222 | 2.8E-09 | 7.66163E-05 | 35.32619 | 461046 |
| all | rs7325293 | C | G | 0.85449 | -0.00683 | 0.001239 | 3.6E-08 | 6.58629E-05 | 30.36772 | 461046 |
| all | rs58231973 | A | G | 0.578058 | 0.005263 | 0.000883 | 2.5E-09 | 7.7056E-05 | 35.52897 | 461046 |
| all | rs35929758 | C | T | 0.602645 | -0.00586 | 0.000895 | 5.8E-11 | 9.30166E-05 | 42.88872 | 461046 |
| all | rs937301 | G | A | 0.44435 | 0.005485 | 0.000878 | 4.2E-10 | 8.45976E-05 | 39.00652 | 461046 |
| all | rs78186330 | A | G | 0.208604 | -0.00687 | 0.001075 | 1.6E-10 | 8.85834E-05 | 40.84448 | 461046 |
| all | rs7503353 | T | G | 0.531723 | -0.00485 | 0.000877 | 3.1E-08 | 6.64746E-05 | 30.64975 | 461046 |
| all | rs1652376 | T | G | 0.462281 | 0.004913 | 0.000875 | 2E-08 | 6.83703E-05 | 31.52388 | 461046 |
| all | rs34647936 | G | T | 0.185389 | -0.00615 | 0.001124 | 4.5E-08 | 6.48763E-05 | 29.91275 | 461046 |
| all | rs41279350 | G | A | 0.127622 | 0.007751 | 0.001309 | 3.2E-09 | 7.60681E-05 | 35.07339 | 461046 |
| all | rs1065852 | A | G | 0.221183 | 0.00636 | 0.00103 | 6.5E-10 | 8.27447E-05 | 38.15208 | 461046 |
| calo | rs2776749 | T | C | 0.556138 | 0.007562 | 0.001835 | 3.8E-05 | 0.000261483 | 16.987 | 64949 |
| calo | rs6713924 | A | G | 0.057006 | -0.01648 | 0.003833 | 0.000017 | 0.000284672 | 18.49385 | 64949 |
| calo | rs4672587 | G | A | 0.321622 | 0.008579 | 0.00193 | 8.7E-06 | 0.000304249 | 19.76604 | 64949 |
| calo | rs7574062 | T | C | 0.065568 | -0.01501 | 0.003635 | 3.6E-05 | 0.000262589 | 17.05888 | 64949 |
| calo | rs11128534 | A | C | 0.305315 | 0.008811 | 0.001939 | 5.5E-06 | 0.000317919 | 20.65443 | 64949 |
| calo | rs830620 | T | C | 0.416427 | -0.00801 | 0.001807 | 9.3E-06 | 0.000302546 | 19.65539 | 64949 |
| calo | rs1994961 | T | C | 0.359952 | 0.007643 | 0.001857 | 3.8E-05 | 0.000260843 | 16.94538 | 64949 |
| calo | rs78723258 | T | C | 0.126634 | 0.011903 | 0.002697 | 0.00001 | 0.000299804 | 19.47723 | 64949 |
| calo | rs116501483 | C | T | 0.060087 | 0.016747 | 0.003808 | 1.1E-05 | 0.000297769 | 19.34498 | 64949 |
| calo | rs34141146 | G | A | 0.204691 | 0.009628 | 0.002214 | 1.4E-05 | 0.000291195 | 18.91776 | 64949 |
| calo | rs9309767 | T | C | 0.343118 | -0.0081 | 0.00189 | 1.8E-05 | 0.000282702 | 18.36586 | 64949 |
| calo | rs11729430 | T | C | 0.254546 | 0.009448 | 0.002065 | 4.8E-06 | 0.000322158 | 20.92996 | 64949 |
| calo | rs4380571 | A | C | 0.196297 | 0.009162 | 0.002256 | 4.9E-05 | 0.000253924 | 16.49576 | 64949 |
| calo | rs62280170 | C | A | 0.076575 | 0.013703 | 0.00336 | 4.5E-05 | 0.000256046 | 16.63367 | 64949 |
| calo | rs3734140 | T | C | 0.515107 | -0.0081 | 0.001829 | 9.6E-06 | 0.000301552 | 19.5908 | 64949 |
| calo | rs10074788 | C | T | 0.264641 | 0.008225 | 0.00202 | 4.7E-05 | 0.00025518 | 16.57739 | 64949 |
| calo | rs75471887 | T | C | 0.076976 | -0.01377 | 0.003339 | 3.7E-05 | 0.000261777 | 17.00606 | 64949 |
| calo | rs13194366 | G | A | 0.067335 | 0.014664 | 0.003584 | 4.3E-05 | 0.00025768 | 16.73984 | 64949 |
| calo | rs12665548 | T | C | 0.063307 | 0.015938 | 0.003705 | 0.000017 | 0.000284822 | 18.50359 | 64949 |
| calo | rs317104 | A | G | 0.778365 | -0.00902 | 0.00216 | 2.9E-05 | 0.000268723 | 17.45747 | 64949 |
| calo | rs62423097 | C | G | 0.130081 | 0.010814 | 0.002647 | 4.4E-05 | 0.0002568 | 16.68265 | 64949 |
| calo | rs12199120 | C | T | 0.064078 | -0.01554 | 0.003694 | 2.6E-05 | 0.000272424 | 17.69793 | 64949 |
| calo | rs9719210 | C | T | 0.471157 | -0.00795 | 0.001786 | 8.5E-06 | 0.00030522 | 19.82917 | 64949 |
| calo | rs6962738 | A | G | 0.234394 | -0.00882 | 0.002147 | 3.9E-05 | 0.000260068 | 16.89505 | 64949 |
| calo | rs17170324 | C | G | 0.155661 | -0.01047 | 0.002458 | 0.00002 | 0.000279456 | 18.15493 | 64949 |
| calo | rs62496292 | G | A | 0.102078 | 0.01294 | 0.002971 | 1.3E-05 | 0.000291909 | 18.96417 | 64949 |
| calo | rs7816237 | T | A | 0.735793 | -0.00838 | 0.002025 | 3.5E-05 | 0.000263672 | 17.12925 | 64949 |
| calo | rs11998486 | A | C | 0.658183 | -0.00771 | 0.001877 | 0.00004 | 0.000259581 | 16.86336 | 64949 |
| calo | rs10117336 | A | C | 0.133184 | 0.012665 | 0.002639 | 1.6E-06 | 0.000354575 | 23.03676 | 64949 |
| calo | rs12376598 | T | C | 0.197863 | 0.012772 | 0.002985 | 1.9E-05 | 0.000281754 | 18.30424 | 64949 |
| calo | rs1571570 | C | G | 0.072176 | -0.0146 | 0.003464 | 0.000025 | 0.000273332 | 17.75693 | 64949 |
| calo | rs7902467 | G | A | 0.510167 | 0.007613 | 0.001786 | 0.00002 | 0.000279656 | 18.16788 | 64949 |
| calo | rs2387595 | T | C | 0.422337 | 0.007622 | 0.001816 | 2.7E-05 | 0.000271011 | 17.60612 | 64949 |
| calo | rs4128662 | C | T | 0.136569 | 0.010823 | 0.0026 | 3.1E-05 | 0.000266804 | 17.33273 | 64949 |
| calo | rs11012730 | A | G | 0.354872 | 0.008721 | 0.001872 | 3.2E-06 | 0.000334202 | 21.71269 | 64949 |
| calo | rs73012993 | G | A | 0.091726 | 0.013721 | 0.003099 | 9.5E-06 | 0.000301704 | 19.6007 | 64949 |
| calo | rs6589774 | A | G | 0.343441 | -0.00807 | 0.001891 | 0.00002 | 0.0002802 | 18.20322 | 64949 |
| calo | rs746541 | T | A | 0.567065 | -0.0077 | 0.001861 | 3.5E-05 | 0.000263374 | 17.10984 | 64949 |
| calo | rs1167688 | A | G | 0.337595 | 0.00841 | 0.001881 | 7.8E-06 | 0.000307661 | 19.9878 | 64949 |
| calo | rs10587831 | C | T | 0.052283 | 0.023704 | 0.005484 | 0.000015 | 0.000287572 | 18.68232 | 64949 |
| calo | rs2857665 | G | C | 0.109664 | 0.011933 | 0.002859 | 3E-05 | 0.000268197 | 17.42328 | 64949 |
| calo | rs9576912 | A | G | 0.661703 | 0.008421 | 0.002027 | 3.3E-05 | 0.000265659 | 17.25836 | 64949 |
| calo | rs9517473 | A | G | 0.694459 | -0.00823 | 0.001936 | 0.000021 | 0.000277943 | 18.05657 | 64949 |
| calo | rs9537509 | C | T | 0.255877 | -0.00856 | 0.002042 | 2.7E-05 | 0.000270717 | 17.58701 | 64949 |
| calo | rs7155478 | G | A | 0.381725 | -0.00865 | 0.001839 | 2.5E-06 | 0.000340786 | 22.14057 | 64949 |
| calo | rs73348837 | A | C | 0.167108 | -0.00991 | 0.002403 | 3.7E-05 | 0.000261763 | 17.00515 | 64949 |
| calo | rs1190821 | T | C | 0.491054 | 0.007701 | 0.00178 | 0.000015 | 0.00028801 | 18.71079 | 64949 |
| calo | rs8037652 | A | G | 0.1149 | -0.01286 | 0.002812 | 4.8E-06 | 0.000322082 | 20.925 | 64949 |
| calo | rs28568418 | A | G | 0.108898 | -0.0118 | 0.002873 | 0.00004 | 0.000259736 | 16.87345 | 64949 |
| calo | rs68125569 | C | T | 0.381622 | 0.008367 | 0.001854 | 6.4E-06 | 0.000313383 | 20.35965 | 64949 |
| calo | rs9889546 | C | G | 0.516314 | 0.008052 | 0.001773 | 5.6E-06 | 0.000317469 | 20.6252 | 64949 |
| calo | rs2037151 | G | A | 0.060748 | 0.018701 | 0.004443 | 2.6E-05 | 0.000272725 | 17.7175 | 64949 |
| calo | rs78588607 | T | G | 0.059022 | -0.01589 | 0.003808 | 3E-05 | 0.000268033 | 17.41261 | 64949 |
| calo | rs7255731 | A | G | 0.279978 | 0.008745 | 0.001998 | 0.000012 | 0.000294796 | 19.15174 | 64949 |
| calo | rs6102949 | A | C | 0.188505 | 0.009332 | 0.002272 | 0.00004 | 0.000259604 | 16.86485 | 64949 |
| calo | rs12165440 | G | A | 0.21011 | 0.009457 | 0.00222 | 0.00002 | 0.000279264 | 18.14244 | 64949 |
| nodairy | rs4240847 | A | C | 0.737477 | 0.001456 | 0.000352 | 3.5E-05 | 3.71477E-05 | 17.12738 | 461046 |
| nodairy | rs3014708 | C | T | 0.061707 | 0.002779 | 0.000642 | 0.000015 | 4.06888E-05 | 18.76009 | 461046 |
| nodairy | rs6688840 | T | C | 0.079531 | -0.00251 | 0.000574 | 0.000012 | 4.14526E-05 | 19.11226 | 461046 |
| nodairy | rs12723846 | A | G | 0.16731 | 0.001784 | 0.000421 | 2.3E-05 | 3.88879E-05 | 17.92975 | 461046 |
| nodairy | rs77045602 | A | G | 0.116472 | -0.00197 | 0.000485 | 4.7E-05 | 3.58968E-05 | 16.55059 | 461046 |
| nodairy | rs4954393 | G | A | 0.080298 | 0.002406 | 0.000569 | 2.3E-05 | 3.88436E-05 | 17.90933 | 461046 |
| nodairy | rs61399217 | C | T | 0.16168 | 0.00174 | 0.00042 | 3.4E-05 | 3.72438E-05 | 17.17169 | 461046 |
| nodairy | rs2194511 | T | C | 0.633718 | 0.001436 | 0.000322 | 8.3E-06 | 4.30932E-05 | 19.86871 | 461046 |
| nodairy | rs6798983 | T | A | 0.754846 | 0.001568 | 0.000361 | 1.4E-05 | 4.10085E-05 | 18.90752 | 461046 |
| nodairy | rs718144 | G | A | 0.119024 | -0.00195 | 0.000478 | 4.7E-05 | 3.59195E-05 | 16.56105 | 461046 |
| nodairy | rs10513210 | T | C | 0.064947 | 0.002591 | 0.000627 | 3.6E-05 | 3.70217E-05 | 17.06926 | 461046 |
| nodairy | rs7614732 | G | T | 0.512606 | -0.00129 | 0.000309 | 2.8E-05 | 3.80598E-05 | 17.54791 | 461046 |
| nodairy | rs59796915 | C | A | 0.059811 | 0.002666 | 0.000652 | 4.3E-05 | 3.63207E-05 | 16.74604 | 461046 |
| nodairy | rs77223212 | A | G | 0.144808 | -0.00202 | 0.000444 | 0.000005 | 4.5199E-05 | 20.83965 | 461046 |
| nodairy | rs13107325 | T | C | 0.074882 | 0.002681 | 0.000587 | 0.000005 | 4.51836E-05 | 20.83257 | 461046 |
| nodairy | rs10056743 | T | C | 0.215736 | 0.001596 | 0.000378 | 2.4E-05 | 3.87173E-05 | 17.85106 | 461046 |
| nodairy | rs2747739 | A | G | 0.280486 | -0.00143 | 0.000344 | 3.3E-05 | 3.74252E-05 | 17.2553 | 461046 |
| nodairy | rs9399158 | T | C | 0.309836 | 0.00139 | 0.000334 | 0.000032 | 3.74726E-05 | 17.27715 | 461046 |
| nodairy | rs87152 | C | T | 0.509471 | -0.00135 | 0.00031 | 1.3E-05 | 4.12413E-05 | 19.01484 | 461046 |
| nodairy | rs2237358 | C | T | 0.135275 | 0.0019 | 0.000453 | 2.8E-05 | 3.80589E-05 | 17.5475 | 461046 |
| nodairy | rs117546412 | T | C | 0.106255 | 0.002105 | 0.000514 | 4.2E-05 | 3.6438E-05 | 16.80013 | 461046 |
| nodairy | rs3801777 | C | G | 0.121991 | -0.00205 | 0.000478 | 0.000017 | 4.00409E-05 | 18.46137 | 461046 |
| nodairy | rs7809842 | G | A | 0.062555 | -0.00273 | 0.00064 | 1.9E-05 | 3.96372E-05 | 18.27523 | 461046 |
| nodairy | rs13310351 | T | C | 0.086422 | -0.00241 | 0.000556 | 0.000015 | 4.07632E-05 | 18.7944 | 461046 |
| nodairy | rs73434111 | G | A | 0.088194 | 0.002357 | 0.000545 | 0.000015 | 4.05348E-05 | 18.68908 | 461046 |
| nodairy | rs6557919 | G | A | 0.686418 | -0.0014 | 0.000337 | 3.3E-05 | 3.73425E-05 | 17.2172 | 461046 |
| nodairy | rs2127807 | T | C | 0.598329 | 0.00141 | 0.000315 | 7.6E-06 | 4.34809E-05 | 20.04747 | 461046 |
| nodairy | rs1489582 | C | A | 0.149434 | -0.00184 | 0.000435 | 2.4E-05 | 3.86282E-05 | 17.81001 | 461046 |
| nodairy | rs1440288 | C | G | 0.663016 | 0.001482 | 0.000328 | 6.1E-06 | 4.4395E-05 | 20.46897 | 461046 |
| nodairy | rs73359182 | G | C | 0.087203 | -0.00226 | 0.000555 | 4.5E-05 | 3.60718E-05 | 16.6313 | 461046 |
| nodairy | rs7024672 | A | G | 0.850765 | -0.00177 | 0.000434 | 4.4E-05 | 3.61701E-05 | 16.67663 | 461046 |
| nodairy | rs4836901 | A | G | 0.878889 | -0.00203 | 0.000475 | 0.00002 | 3.95003E-05 | 18.21209 | 461046 |
| nodairy | rs1547190 | T | C | 0.548817 | -0.00161 | 0.00031 | 2E-07 | 5.85581E-05 | 26.99944 | 461046 |
| nodairy | rs62534709 | T | C | 0.141473 | -0.00218 | 0.000444 | 8.9E-07 | 5.23852E-05 | 24.15313 | 461046 |
| nodairy | rs2296826 | G | T | 0.407695 | -0.0014 | 0.000316 | 9.6E-06 | 4.24953E-05 | 19.59304 | 461046 |
| nodairy | rs2804602 | G | A | 0.091543 | -0.00227 | 0.000537 | 2.2E-05 | 3.89702E-05 | 17.96767 | 461046 |
| nodairy | rs34690395 | A | G | 0.216895 | -0.00155 | 0.000378 | 4.2E-05 | 3.63759E-05 | 16.77151 | 461046 |
| nodairy | rs35710625 | C | T | 0.545558 | -0.00141 | 0.000312 | 5.8E-06 | 4.45613E-05 | 20.54565 | 461046 |
| nodairy | rs1676752 | T | G | 0.878906 | 0.002031 | 0.000473 | 0.000017 | 4.00088E-05 | 18.44657 | 461046 |
| nodairy | rs10742615 | T | G | 0.773924 | -0.00172 | 0.000369 | 3.2E-06 | 4.69893E-05 | 21.66514 | 461046 |
| nodairy | rs116920960 | A | G | 0.051565 | 0.003185 | 0.000701 | 5.5E-06 | 4.48272E-05 | 20.66823 | 461046 |
| nodairy | rs111417789 | A | G | 0.081141 | 0.002411 | 0.000575 | 2.7E-05 | 3.81679E-05 | 17.59777 | 461046 |
| nodairy | rs113765952 | G | A | 0.086192 | -0.00242 | 0.000562 | 0.000017 | 4.0102E-05 | 18.48951 | 461046 |
| nodairy | rs12367587 | A | G | 0.061549 | -0.00272 | 0.000663 | 0.00004 | 3.65535E-05 | 16.8534 | 461046 |
| nodairy | rs11043241 | G | A | 0.535102 | -0.00133 | 0.00031 | 1.9E-05 | 3.97697E-05 | 18.33631 | 461046 |
| nodairy | rs74711508 | T | G | 0.053194 | 0.002865 | 0.000689 | 0.000032 | 3.74499E-05 | 17.2667 | 461046 |
| nodairy | rs3803256 | T | C | 0.431478 | 0.001311 | 0.000311 | 0.000025 | 3.8448E-05 | 17.72692 | 461046 |
| nodairy | rs4517701 | T | G | 0.733655 | -0.00147 | 0.00035 | 2.9E-05 | 3.79696E-05 | 17.50634 | 461046 |
| nodairy | rs1118964 | T | C | 0.479492 | 0.001312 | 0.00031 | 2.4E-05 | 3.87532E-05 | 17.86762 | 461046 |
| nodairy | rs12911725 | A | G | 0.248894 | -0.00162 | 0.000357 | 5.9E-06 | 4.45034E-05 | 20.51893 | 461046 |
| nodairy | rs117217816 | A | T | 0.120539 | -0.00217 | 0.000477 | 5.5E-06 | 4.48044E-05 | 20.65773 | 461046 |
| nodairy | rs55746941 | T | G | 0.059556 | -0.00276 | 0.00066 | 2.9E-05 | 3.79856E-05 | 17.51368 | 461046 |
| nodairy | rs117786076 | G | A | 0.076454 | 0.002459 | 0.000599 | 0.00004 | 3.65447E-05 | 16.84935 | 461046 |
| nodairy | rs12716937 | G | A | 0.422759 | 0.001302 | 0.000314 | 3.3E-05 | 3.73811E-05 | 17.23498 | 461046 |
| nodairy | rs56343657 | G | A | 0.121744 | 0.001981 | 0.000473 | 2.8E-05 | 3.80815E-05 | 17.55794 | 461046 |
| nodairy | rs76079816 | G | A | 0.095657 | -0.00226 | 0.00053 | 1.9E-05 | 3.96056E-05 | 18.26064 | 461046 |
| nodairy | rs8072509 | G | T | 0.921015 | -0.0024 | 0.000576 | 3.1E-05 | 3.76378E-05 | 17.35333 | 461046 |
| nodairy | rs4969252 | C | T | 0.263428 | -0.00147 | 0.000354 | 3.4E-05 | 3.72865E-05 | 17.19136 | 461046 |
| nodairy | rs12960460 | C | T | 0.126711 | 0.001913 | 0.000464 | 3.8E-05 | 3.68488E-05 | 16.98954 | 461046 |
| nodairy | rs8096948 | C | T | 0.214716 | 0.001572 | 0.000377 | 3E-05 | 3.7821E-05 | 17.43781 | 461046 |
| nodairy | rs56385135 | C | G | 0.240914 | -0.00159 | 0.000362 | 1.1E-05 | 4.20437E-05 | 19.38479 | 461046 |
| nodairy | rs279448 | G | A | 0.171368 | 0.001751 | 0.000417 | 2.6E-05 | 3.83272E-05 | 17.67121 | 461046 |
| nodairy | rs10414043 | A | G | 0.126632 | 0.002038 | 0.000466 | 0.000012 | 4.15415E-05 | 19.15326 | 461046 |
| nodairy | rs2297197 | G | C | 0.285909 | 0.001513 | 0.000346 | 0.000012 | 4.14205E-05 | 19.09747 | 461046 |
| nodairy | rs111924272 | G | A | 0.074073 | 0.002433 | 0.00059 | 3.7E-05 | 3.68865E-05 | 17.00691 | 461046 |
| nodairy | rs1475862 | G | T | 0.277898 | 0.001404 | 0.000345 | 4.7E-05 | 3.58868E-05 | 16.54599 | 461046 |
| nodairy | rs2849942 | T | C | 0.255423 | -0.00149 | 0.000356 | 2.9E-05 | 3.79862E-05 | 17.51399 | 461046 |
| nodairy | rs11090148 | T | C | 0.488884 | -0.00141 | 0.000313 | 6.1E-06 | 4.43755E-05 | 20.45995 | 461046 |
| noegg | rs72667460 | T | C | 0.055493 | -0.00334 | 0.00073 | 4.6E-06 | 4.55141E-05 | 20.98494 | 461046 |
| noegg | rs197603 | T | C | 0.411673 | -0.0014 | 0.000339 | 3.6E-05 | 3.7031E-05 | 17.07355 | 461046 |
| noegg | rs10733052 | G | C | 0.70847 | 0.001732 | 0.000366 | 2.3E-06 | 4.84778E-05 | 22.35147 | 461046 |
| noegg | rs9425441 | G | A | 0.055891 | -0.00306 | 0.000724 | 2.4E-05 | 3.8777E-05 | 17.87861 | 461046 |
| noegg | rs1200121 | G | A | 0.075874 | 0.002821 | 0.000637 | 9.5E-06 | 4.25239E-05 | 19.60622 | 461046 |
| noegg | rs3008403 | G | A | 0.907764 | -0.00237 | 0.000577 | 0.00004 | 3.66155E-05 | 16.88199 | 461046 |
| noegg | rs2352723 | C | T | 0.809293 | -0.00177 | 0.000427 | 0.000032 | 3.74866E-05 | 17.2836 | 461046 |
| noegg | rs910766 | A | C | 0.584152 | 0.001386 | 0.000339 | 4.4E-05 | 3.62041E-05 | 16.69227 | 461046 |
| noegg | rs2257101 | G | T | 0.428894 | 0.00138 | 0.000337 | 4.2E-05 | 3.64409E-05 | 16.80145 | 461046 |
| noegg | rs479125 | A | T | 0.235264 | 0.00165 | 0.000393 | 2.6E-05 | 3.82928E-05 | 17.65533 | 461046 |
| noegg | rs12568865 | T | A | 0.134305 | 0.00201 | 0.000492 | 4.5E-05 | 3.61179E-05 | 16.65254 | 461046 |
| noegg | rs11889271 | A | G | 0.062075 | -0.00313 | 0.000691 | 5.8E-06 | 4.45717E-05 | 20.55042 | 461046 |
| noegg | rs7595223 | C | T | 0.845117 | -0.00195 | 0.00046 | 2.3E-05 | 3.88346E-05 | 17.90517 | 461046 |
| noegg | rs11896394 | A | G | 0.292384 | -0.00152 | 0.000367 | 3.7E-05 | 3.68823E-05 | 17.00499 | 461046 |
| noegg | rs2048206 | A | C | 0.210148 | 0.001962 | 0.000409 | 1.6E-06 | 4.98539E-05 | 22.98598 | 461046 |
| noegg | rs6774732 | C | T | 0.141014 | -0.00198 | 0.000479 | 3.7E-05 | 3.68695E-05 | 16.99909 | 461046 |
| noegg | rs535526 | T | C | 0.944404 | -0.00328 | 0.000728 | 6.6E-06 | 4.401E-05 | 20.29144 | 461046 |
| noegg | rs1066602 | G | A | 0.735334 | -0.00155 | 0.000378 | 4.2E-05 | 3.63941E-05 | 16.7799 | 461046 |
| noegg | rs4686706 | A | G | 0.071057 | 0.002732 | 0.000655 | 3E-05 | 3.771E-05 | 17.38663 | 461046 |
| noegg | rs6786206 | C | T | 0.857847 | -0.00195 | 0.000478 | 4.3E-05 | 3.62579E-05 | 16.71708 | 461046 |
| noegg | rs1655062 | A | G | 0.152321 | -0.00198 | 0.000465 | 0.00002 | 3.94853E-05 | 18.20516 | 461046 |
| noegg | rs6857642 | A | G | 0.825901 | -0.00208 | 0.000441 | 2.3E-06 | 4.84221E-05 | 22.3258 | 461046 |
| noegg | rs62301644 | A | G | 0.095234 | -0.00244 | 0.000575 | 2.2E-05 | 3.91429E-05 | 18.0473 | 461046 |
| noegg | rs10014233 | C | T | 0.670016 | -0.00145 | 0.000358 | 4.9E-05 | 3.57375E-05 | 16.47714 | 461046 |
| noegg | rs28460491 | G | T | 0.241514 | -0.0018 | 0.00039 | 4.1E-06 | 4.60083E-05 | 21.21281 | 461046 |
| noegg | rs1455292 | C | T | 0.90383 | -0.00247 | 0.000568 | 1.4E-05 | 4.1063E-05 | 18.93265 | 461046 |
| noegg | rs10035149 | A | G | 0.107454 | 0.002353 | 0.000539 | 1.3E-05 | 4.13297E-05 | 19.0556 | 461046 |
| noegg | rs329193 | A | G | 0.509395 | -0.00136 | 0.000333 | 4.8E-05 | 3.58778E-05 | 16.54185 | 461046 |
| noegg | rs72816043 | G | T | 0.092479 | -0.00234 | 0.000575 | 4.9E-05 | 3.57734E-05 | 16.49368 | 461046 |
| noegg | rs9273393 | A | G | 0.101363 | 0.002596 | 0.000632 | 4.1E-05 | 3.65359E-05 | 16.84525 | 461046 |
| noegg | rs2268442 | A | G | 0.408648 | -0.00148 | 0.000348 | 0.00002 | 3.93699E-05 | 18.15195 | 461046 |
| noegg | rs7752833 | T | C | 0.802491 | 0.001833 | 0.000418 | 1.1E-05 | 4.18124E-05 | 19.27815 | 461046 |
| noegg | rs34557294 | C | T | 0.112593 | -0.00231 | 0.000542 | 0.000021 | 3.91965E-05 | 18.07203 | 461046 |
| noegg | rs142863425 | T | C | 0.090146 | 0.002549 | 0.000581 | 1.1E-05 | 4.17609E-05 | 19.25443 | 461046 |
| noegg | rs6912704 | A | G | 0.115033 | -0.00228 | 0.000526 | 0.000015 | 4.06717E-05 | 18.7522 | 461046 |
| noegg | rs6940301 | G | A | 0.831332 | -0.00188 | 0.000449 | 2.9E-05 | 3.7926E-05 | 17.48622 | 461046 |
| noegg | rs2613121 | G | T | 0.516077 | -0.00167 | 0.000338 | 8.3E-07 | 5.26933E-05 | 24.29519 | 461046 |
| noegg | rs7742212 | A | G | 0.562483 | 0.001457 | 0.000339 | 0.000017 | 4.01588E-05 | 18.51571 | 461046 |
| noegg | rs10249355 | T | C | 0.819685 | -0.00189 | 0.000434 | 1.3E-05 | 4.13186E-05 | 19.0505 | 461046 |
| noegg | rs6963101 | G | A | 0.257676 | -0.00161 | 0.000381 | 2.4E-05 | 3.87128E-05 | 17.84901 | 461046 |
| noegg | rs141018170 | T | C | 0.059729 | 0.002883 | 0.00071 | 4.9E-05 | 3.57839E-05 | 16.49856 | 461046 |
| noegg | rs113905912 | C | T | 0.342603 | -0.00148 | 0.000351 | 2.6E-05 | 3.83171E-05 | 17.66653 | 461046 |
| noegg | rs79752055 | G | A | 0.053233 | 0.003182 | 0.000748 | 0.000021 | 3.92616E-05 | 18.10202 | 461046 |
| noegg | rs73100394 | G | A | 0.174356 | 0.001872 | 0.000441 | 2.2E-05 | 3.89961E-05 | 17.97961 | 461046 |
| noegg | rs12544890 | G | A | 0.86525 | -0.00221 | 0.000488 | 5.7E-06 | 4.46156E-05 | 20.57069 | 461046 |
| noegg | rs2003213 | C | G | 0.182558 | 0.001753 | 0.000432 | 4.9E-05 | 3.57815E-05 | 16.49743 | 461046 |
| noegg | rs10987123 | G | C | 0.523884 | -0.00143 | 0.000336 | 0.00002 | 3.94809E-05 | 18.20314 | 461046 |
| noegg | rs57183880 | A | G | 0.474982 | -0.0015 | 0.000335 | 7.7E-06 | 4.34041E-05 | 20.01205 | 461046 |
| noegg | rs4877210 | G | C | 0.060393 | -0.0029 | 0.000699 | 3.3E-05 | 3.73256E-05 | 17.20937 | 461046 |
| noegg | rs2890718 | A | G | 0.485362 | -0.00141 | 0.000337 | 2.8E-05 | 3.80296E-05 | 17.53399 | 461046 |
| noegg | rs4918162 | C | T | 0.572343 | 0.0014 | 0.000336 | 3.1E-05 | 3.75882E-05 | 17.33045 | 461046 |
| noegg | rs56012318 | T | C | 0.59269 | -0.00149 | 0.000341 | 0.000012 | 4.16591E-05 | 19.20747 | 461046 |
| noegg | rs7070663 | T | C | 0.162483 | 0.001961 | 0.000452 | 1.4E-05 | 4.08461E-05 | 18.83263 | 461046 |
| noegg | rs5030883 | C | A | 0.212317 | 0.001753 | 0.000407 | 0.000017 | 4.01497E-05 | 18.51154 | 461046 |
| noegg | rs2508681 | G | T | 0.763267 | -0.00198 | 0.000412 | 1.6E-06 | 5.00648E-05 | 23.08324 | 461046 |
| noegg | rs7935823 | G | A | 0.361738 | -0.00141 | 0.000347 | 4.8E-05 | 3.58428E-05 | 16.5257 | 461046 |
| noegg | rs2098844 | T | C | 0.534002 | 0.001462 | 0.000336 | 1.3E-05 | 4.11181E-05 | 18.95805 | 461046 |
| noegg | rs7137775 | G | A | 0.152785 | -0.00196 | 0.000467 | 2.6E-05 | 3.8327E-05 | 17.67111 | 461046 |
| noegg | rs74089557 | A | G | 0.060665 | -0.00292 | 0.000698 | 2.9E-05 | 3.79229E-05 | 17.4848 | 461046 |
| noegg | rs138062324 | A | G | 0.146742 | -0.00202 | 0.000474 | 1.9E-05 | 3.96243E-05 | 18.26926 | 461046 |
| noegg | rs1344431 | G | T | 0.585517 | -0.00153 | 0.000339 | 6.1E-06 | 4.43729E-05 | 20.45876 | 461046 |
| noegg | rs12429072 | C | G | 0.237258 | -0.00166 | 0.000392 | 2.2E-05 | 3.90244E-05 | 17.99266 | 461046 |
| noegg | rs9572817 | T | C | 0.085262 | -0.00249 | 0.000604 | 3.9E-05 | 3.67218E-05 | 16.931 | 461046 |
| noegg | rs945345 | A | C | 0.867494 | -0.00201 | 0.000492 | 4.5E-05 | 3.6152E-05 | 16.66828 | 461046 |
| noegg | rs1242937 | T | C | 0.154695 | -0.00197 | 0.000462 | 0.00002 | 3.95416E-05 | 18.23112 | 461046 |
| noegg | rs13329672 | T | C | 0.266059 | -0.00154 | 0.000378 | 4.8E-05 | 3.58739E-05 | 16.54006 | 461046 |
| noegg | rs4843134 | T | A | 0.471126 | -0.00138 | 0.000338 | 4.6E-05 | 3.59921E-05 | 16.59452 | 461046 |
| noegg | rs1429750 | C | T | 0.678575 | 0.001511 | 0.00036 | 2.7E-05 | 3.82784E-05 | 17.64872 | 461046 |
| noegg | rs140078626 | G | C | 0.058227 | -0.00309 | 0.000759 | 4.7E-05 | 3.58987E-05 | 16.55149 | 461046 |
| noegg | rs9938970 | G | C | 0.635525 | 0.001565 | 0.000346 | 6E-06 | 4.44522E-05 | 20.49533 | 461046 |
| noegg | rs11643584 | G | C | 0.37706 | 0.001427 | 0.000345 | 3.4E-05 | 3.72249E-05 | 17.16296 | 461046 |
| noegg | rs7203740 | T | C | 0.843752 | -0.00214 | 0.000458 | 3.1E-06 | 4.72108E-05 | 21.76727 | 461046 |
| noegg | rs12926653 | A | G | 0.170123 | 0.001874 | 0.000446 | 2.6E-05 | 3.83172E-05 | 17.66659 | 461046 |
| noegg | rs34106818 | G | C | 0.327029 | -0.00145 | 0.000356 | 4.6E-05 | 3.60599E-05 | 16.6258 | 461046 |
| noegg | rs4794660 | G | A | 0.618465 | 0.001708 | 0.000386 | 9.7E-06 | 4.2429E-05 | 19.56248 | 461046 |
| noegg | rs8092765 | T | C | 0.083359 | 0.00259 | 0.000603 | 0.000017 | 4.00236E-05 | 18.4534 | 461046 |
| noegg | rs569047 | T | A | 0.928324 | 0.002927 | 0.000698 | 2.7E-05 | 3.81806E-05 | 17.60359 | 461046 |
| noegg | rs1157347 | C | T | 0.203304 | 0.001788 | 0.00042 | 0.000021 | 3.92436E-05 | 18.09375 | 461046 |
| noegg | rs132472 | G | A | 0.05763 | 0.003051 | 0.000713 | 1.9E-05 | 3.96583E-05 | 18.28494 | 461046 |
| noegg | rs12165902 | G | C | 0.115589 | 0.002216 | 0.000522 | 2.2E-05 | 3.90764E-05 | 18.01665 | 461046 |
| gluten | rs2148682 | C | T | 0.342146 | -0.00377 | 0.000843 | 7.6E-06 | 0.000308217 | 20.02393 | 64949 |
| gluten | rs1151803 | G | A | 0.410831 | 0.003415 | 0.00082 | 3.1E-05 | 0.000266841 | 17.33513 | 64949 |
| gluten | rs13418052 | T | G | 0.325488 | 0.003605 | 0.000851 | 2.3E-05 | 0.000275894 | 17.92346 | 64949 |
| gluten | rs17355592 | G | A | 0.346195 | 0.003429 | 0.000839 | 4.3E-05 | 0.000257339 | 16.7177 | 64949 |
| gluten | rs10018536 | C | G | 0.466617 | -0.00348 | 0.000802 | 1.4E-05 | 0.000290749 | 18.88875 | 64949 |
| gluten | rs1639409 | C | T | 0.6754 | 0.003656 | 0.000858 | 0.00002 | 0.00027953 | 18.15972 | 64949 |
| gluten | rs9271842 | A | C | 0.391156 | 0.005816 | 0.000882 | 4.3E-11 | 0.000669103 | 43.48533 | 64949 |
| gluten | rs763188 | C | T | 0.57506 | -0.00346 | 0.000808 | 1.9E-05 | 0.000282155 | 18.33029 | 64949 |
| gluten | rs1548306 | T | A | 0.666268 | -0.00727 | 0.000849 | 1.1E-17 | 0.001127925 | 73.33804 | 64949 |
| gluten | rs9273595 | G | C | 0.261591 | 0.010164 | 0.000919 | 2.1E-28 | 0.001878017 | 122.2011 | 64949 |
| gluten | rs9277568 | C | T | 0.305637 | 0.004676 | 0.000864 | 6.3E-08 | 0.000450277 | 29.25733 | 64949 |
| gluten | rs28691018 | G | A | 0.318868 | 0.003611 | 0.000856 | 2.4E-05 | 0.00027406 | 17.80423 | 64949 |
| gluten | rs2282910 | T | C | 0.665719 | -0.00384 | 0.00085 | 6.4E-06 | 0.000313518 | 20.36843 | 64949 |
| gluten | rs7962776 | T | C | 0.564364 | -0.00329 | 0.000808 | 4.8E-05 | 0.000254279 | 16.51887 | 64949 |
| gluten | rs12021132 | G | A | 0.463905 | -0.00335 | 0.000812 | 3.7E-05 | 0.000261773 | 17.00583 | 64949 |
| gluten | rs162654 | T | C | 0.610404 | -0.00336 | 0.000824 | 4.5E-05 | 0.000256579 | 16.66828 | 64949 |
| nosugar | rs898980 | T | C | 0.877965 | 0.006831 | 0.001245 | 4.1E-08 | 6.53171E-05 | 30.116 | 461046 |
| nosugar | rs11897809 | C | A | 0.35418 | 0.004795 | 0.00085 | 1.7E-08 | 6.90431E-05 | 31.83411 | 461046 |
| nosugar | rs13026283 | T | C | 0.393454 | -0.00485 | 0.000833 | 5.8E-09 | 7.34995E-05 | 33.88901 | 461046 |
| nosugar | rs17031936 | G | A | 0.237496 | -0.00609 | 0.000956 | 1.8E-10 | 8.81161E-05 | 40.62898 | 461046 |
| nosugar | rs2748287 | A | G | 0.473693 | 0.004487 | 0.000816 | 3.8E-08 | 6.55583E-05 | 30.22726 | 461046 |
| nosugar | rs6458247 | G | A | 0.732251 | 0.005525 | 0.000918 | 1.7E-09 | 7.86126E-05 | 36.24674 | 461046 |
| nosugar | rs7794036 | C | T | 0.529654 | -0.00455 | 0.000815 | 2.3E-08 | 6.76548E-05 | 31.19393 | 461046 |
| nosugar | rs10962171 | A | G | 0.463127 | 0.004762 | 0.000816 | 5.4E-09 | 7.38445E-05 | 34.0481 | 461046 |
| nosugar | rs56348580 | C | G | 0.307311 | -0.00498 | 0.000883 | 1.7E-08 | 6.89854E-05 | 31.8075 | 461046 |
| nosugar | rs360180 | G | T | 0.610617 | -0.00548 | 0.000834 | 4.8E-11 | 9.37979E-05 | 43.24901 | 461046 |
| nosugar | rs12950328 | T | C | 0.539392 | 0.0048 | 0.000815 | 3.9E-09 | 7.51522E-05 | 34.65106 | 461046 |
| nosugar | rs12938531 | G | A | 0.502832 | 0.004788 | 0.000813 | 4E-09 | 7.51159E-05 | 34.63432 | 461046 |
| nosugar | rs4078247 | C | T | 0.222064 | -0.00525 | 0.000957 | 4.1E-08 | 6.52408E-05 | 30.08085 | 461046 |
| vege | rs12121178 | T | A | 0.25634 | -0.00504 | 0.001161 | 1.4E-05 | 0.000290448 | 18.8692 | 64949 |
| vege | rs10875135 | G | A | 0.426938 | -0.00417 | 0.001021 | 4.4E-05 | 0.000256885 | 16.6882 | 64949 |
| vege | rs17033934 | C | T | 0.319946 | 0.004813 | 0.001091 | 0.00001 | 0.000299456 | 19.45457 | 64949 |
| vege | rs12036939 | G | T | 0.188702 | 0.005282 | 0.001282 | 3.8E-05 | 0.000261149 | 16.96526 | 64949 |
| vege | rs34478580 | A | G | 0.460013 | 0.004194 | 0.001013 | 3.5E-05 | 0.000263752 | 17.13444 | 64949 |
| vege | rs79918449 | T | G | 0.218426 | -0.00501 | 0.001218 | 3.9E-05 | 0.000260179 | 16.90224 | 64949 |
| vege | rs61364479 | T | C | 0.216581 | -0.00498 | 0.001215 | 4.2E-05 | 0.000258278 | 16.77874 | 64949 |
| vege | rs700613 | C | A | 0.305836 | 0.004486 | 0.001093 | 4.1E-05 | 0.000259153 | 16.83559 | 64949 |
| vege | rs2400169 | C | T | 0.598946 | 0.004483 | 0.001026 | 1.3E-05 | 0.000293543 | 19.07034 | 64949 |
| vege | rs62383426 | T | C | 0.202352 | 0.005424 | 0.001257 | 0.000016 | 0.000286652 | 18.62251 | 64949 |
| vege | rs7705570 | G | A | 0.218745 | -0.00513 | 0.001217 | 0.000025 | 0.000273469 | 17.76582 | 64949 |
| vege | rs757257 | A | G | 0.47622 | -0.00419 | 0.001 | 2.8E-05 | 0.000270514 | 17.57385 | 64949 |
| vege | rs6980833 | T | C | 0.385279 | 0.004256 | 0.001027 | 3.4E-05 | 0.00026412 | 17.15834 | 64949 |
| vege | rs10978745 | C | T | 0.206786 | 0.005246 | 0.001286 | 4.5E-05 | 0.000256043 | 16.63346 | 64949 |
| vege | rs1219939 | T | C | 0.170804 | 0.005937 | 0.001336 | 8.9E-06 | 0.000303759 | 19.73424 | 64949 |
| vege | rs6477097 | C | A | 0.354375 | 0.004511 | 0.001061 | 0.000021 | 0.000278143 | 18.06959 | 64949 |
| vege | rs11562314 | T | C | 0.758171 | -0.00496 | 0.001207 | 0.00004 | 0.000259525 | 16.85973 | 64949 |
| vege | rs1220819 | A | G | 0.761296 | -0.00479 | 0.001175 | 4.6E-05 | 0.00025586 | 16.6216 | 64949 |
| vege | rs893587 | G | A | 0.67859 | 0.004652 | 0.001081 | 0.000017 | 0.000285317 | 18.53579 | 64949 |
| vege | rs67761120 | C | T | 0.273678 | 0.004613 | 0.001134 | 4.7E-05 | 0.000254831 | 16.55474 | 64949 |
| vege | rs664596 | A | C | 0.264373 | 0.00474 | 0.001141 | 0.000032 | 0.000265796 | 17.26727 | 64949 |
| vege | rs7936649 | G | T | 0.713186 | -0.00513 | 0.001141 | 6.9E-06 | 0.000311416 | 20.23183 | 64949 |
| vege | rs4118073 | A | G | 0.310775 | 0.004734 | 0.001115 | 2.2E-05 | 0.000277375 | 18.01967 | 64949 |
| vege | rs2955524 | T | C | 0.220786 | -0.00499 | 0.001214 | 3.9E-05 | 0.000260207 | 16.90405 | 64949 |
| vege | rs11115556 | G | A | 0.401998 | 0.004208 | 0.001033 | 4.6E-05 | 0.000255635 | 16.60697 | 64949 |
| vege | rs9796292 | A | G | 0.395558 | -0.00449 | 0.001029 | 1.3E-05 | 0.000292633 | 19.0112 | 64949 |
| vege | rs11631964 | G | A | 0.704528 | 0.004728 | 0.001135 | 3.1E-05 | 0.000267324 | 17.36656 | 64949 |
| vege | rs7168044 | C | T | 0.592967 | -0.00428 | 0.001024 | 3E-05 | 0.000268311 | 17.43069 | 64949 |
| vege | rs62035995 | A | G | 0.545649 | -0.00424 | 0.001013 | 2.9E-05 | 0.000269426 | 17.5031 | 64949 |
| vege | rs2526526 | G | A | 0.764716 | 0.004858 | 0.001196 | 4.9E-05 | 0.000253902 | 16.49434 | 64949 |
| vege | rs9303272 | G | A | 0.449881 | -0.00421 | 0.001013 | 0.000032 | 0.00026587 | 17.27204 | 64949 |
| vege | rs8072720 | A | T | 0.716636 | 0.005134 | 0.001175 | 0.000012 | 0.000294012 | 19.10082 | 64949 |
| vege | rs2252425 | T | C | 0.40192 | 0.004178 | 0.001027 | 4.7E-05 | 0.000254958 | 16.563 | 64949 |
| vege | rs846222 | T | C | 0.212209 | 0.005114 | 0.001233 | 3.3E-05 | 0.000264931 | 17.21105 | 64949 |
| vege | rs1701003 | G | C | 0.382634 | 0.004524 | 0.001042 | 1.4E-05 | 0.000290322 | 18.86105 | 64949 |
| vege | rs2833149 | C | T | 0.296608 | -0.0048 | 0.001102 | 1.3E-05 | 0.000291602 | 18.9442 | 64949 |
| vege | rs932302 | T | G | 0.301705 | -0.00561 | 0.001164 | 1.4E-06 | 0.000357775 | 23.24473 | 64949 |
| nowheat | rs591979 | T | C | 0.505715 | 0.001473 | 0.000339 | 1.4E-05 | 4.10267E-05 | 18.9159 | 461046 |
| nowheat | rs10916019 | A | G | 0.384433 | 0.001511 | 0.000358 | 2.4E-05 | 3.872E-05 | 17.85232 | 461046 |
| nowheat | rs15396 | C | A | 0.073673 | 0.002909 | 0.000644 | 6.3E-06 | 4.42167E-05 | 20.38675 | 461046 |
| nowheat | rs60585655 | T | C | 0.536518 | -0.00138 | 0.000339 | 4.7E-05 | 3.59118E-05 | 16.55752 | 461046 |
| nowheat | rs4652192 | A | C | 0.19136 | -0.00185 | 0.000432 | 1.8E-05 | 3.98175E-05 | 18.35833 | 461046 |
| nowheat | rs11683213 | C | A | 0.452015 | -0.0015 | 0.000342 | 1.1E-05 | 4.19583E-05 | 19.34545 | 461046 |
| nowheat | rs4952777 | C | T | 0.290587 | 0.001548 | 0.000376 | 3.8E-05 | 3.68238E-05 | 16.97803 | 461046 |
| nowheat | rs13419848 | G | T | 0.30561 | 0.001704 | 0.000371 | 4.5E-06 | 4.56099E-05 | 21.02912 | 461046 |
| nowheat | rs62171692 | A | G | 0.443527 | 0.001412 | 0.00034 | 3.3E-05 | 3.74374E-05 | 17.26096 | 461046 |
| nowheat | rs77586865 | T | C | 0.059445 | 0.003111 | 0.000713 | 1.3E-05 | 4.12829E-05 | 19.03401 | 461046 |
| nowheat | rs1708137 | A | T | 0.350142 | 0.001456 | 0.000355 | 4.1E-05 | 3.64428E-05 | 16.80233 | 461046 |
| nowheat | rs73210326 | C | T | 0.227873 | -0.00163 | 0.000402 | 4.9E-05 | 3.57365E-05 | 16.47668 | 461046 |
| nowheat | rs56030108 | A | G | 0.066796 | -0.00277 | 0.000675 | 4.2E-05 | 3.64055E-05 | 16.78514 | 461046 |
| nowheat | rs145964640 | G | C | 0.060551 | 0.003099 | 0.000724 | 1.9E-05 | 3.97556E-05 | 18.32979 | 461046 |
| nowheat | rs17608637 | C | T | 0.274415 | 0.001551 | 0.000377 | 0.00004 | 3.66161E-05 | 16.88223 | 461046 |
| nowheat | rs2136947 | C | T | 0.06468 | -0.00279 | 0.000685 | 4.6E-05 | 3.60017E-05 | 16.59899 | 461046 |
| nowheat | rs13179146 | T | C | 0.194162 | 0.001762 | 0.000429 | 3.9E-05 | 3.66541E-05 | 16.89979 | 461046 |
| nowheat | rs487462 | C | G | 0.658178 | 0.001592 | 0.000357 | 8.3E-06 | 4.31099E-05 | 19.87641 | 461046 |
| nowheat | rs619653 | A | C | 0.20713 | -0.00181 | 0.000418 | 0.000016 | 4.04616E-05 | 18.65534 | 461046 |
| nowheat | rs742870 | C | T | 0.470007 | 0.002363 | 0.000338 | 2.7E-12 | 0.000106072 | 48.90928 | 461046 |
| nowheat | rs2854275 | A | C | 0.146173 | 0.012351 | 0.000476 | 3.7E-148 | 0.0014554 | 671.9814 | 461046 |
| nowheat | rs545686632 | A | G | 0.287931 | 0.007159 | 0.000432 | 8.8E-62 | 0.000596308 | 275.0881 | 461046 |
| nowheat | rs2516486 | G | A | 0.116098 | -0.00221 | 0.000525 | 0.000025 | 3.84856E-05 | 17.74425 | 461046 |
| nowheat | rs36020935 | T | G | 0.199675 | 0.006136 | 0.000517 | 1.5E-32 | 0.000305908 | 141.0802 | 461046 |
| nowheat | rs2111847 | G | A | 0.801021 | 0.001848 | 0.000427 | 0.000015 | 4.06138E-05 | 18.72551 | 461046 |
| nowheat | rs79357656 | T | A | 0.053883 | 0.003242 | 0.000756 | 1.8E-05 | 3.98841E-05 | 18.38904 | 461046 |
| nowheat | rs10260897 | G | A | 0.241904 | -0.00167 | 0.000399 | 2.8E-05 | 3.79938E-05 | 17.51749 | 461046 |
| nowheat | rs13281534 | G | A | 0.15108 | -0.00205 | 0.000477 | 1.8E-05 | 3.98334E-05 | 18.36568 | 461046 |
| nowheat | rs113007364 | C | T | 0.076328 | 0.002753 | 0.000634 | 1.4E-05 | 4.0885E-05 | 18.85055 | 461046 |
| nowheat | rs6990907 | G | A | 0.488958 | 0.001699 | 0.000338 | 4.9E-07 | 5.48855E-05 | 25.306 | 461046 |
| nowheat | rs7002825 | C | T | 0.209081 | -0.00183 | 0.000424 | 0.000016 | 4.02878E-05 | 18.5752 | 461046 |
| nowheat | rs2725341 | A | T | 0.807571 | -0.0018 | 0.000428 | 2.6E-05 | 3.83547E-05 | 17.68391 | 461046 |
| nowheat | rs7814652 | A | G | 0.200649 | -0.00176 | 0.000421 | 2.9E-05 | 3.78762E-05 | 17.46324 | 461046 |
| nowheat | rs10991932 | G | A | 0.225191 | 0.001785 | 0.000405 | 1.1E-05 | 4.20801E-05 | 19.40157 | 461046 |
| nowheat | rs303730 | C | T | 0.937687 | -0.00308 | 0.000707 | 1.3E-05 | 4.12216E-05 | 19.00578 | 461046 |
| nowheat | rs72718940 | T | G | 0.050876 | 0.003308 | 0.000775 | 0.00002 | 3.95365E-05 | 18.22877 | 461046 |
| nowheat | rs2208699 | G | A | 0.618326 | -0.00145 | 0.000348 | 0.000032 | 3.75301E-05 | 17.30366 | 461046 |
| nowheat | rs10813804 | A | G | 0.184384 | 0.001841 | 0.000439 | 2.7E-05 | 3.82088E-05 | 17.61661 | 461046 |
| nowheat | rs7092430 | G | A | 0.927271 | -0.00283 | 0.000655 | 0.000015 | 4.05334E-05 | 18.68845 | 461046 |
| nowheat | rs12764457 | G | A | 0.176654 | -0.00191 | 0.000443 | 0.000016 | 4.0342E-05 | 18.60018 | 461046 |
| nowheat | rs2224375 | G | C | 0.302497 | -0.00149 | 0.000367 | 4.6E-05 | 3.60018E-05 | 16.59901 | 461046 |
| nowheat | rs12774516 | C | T | 0.309256 | 0.001617 | 0.000366 | 9.9E-06 | 4.23459E-05 | 19.52415 | 461046 |
| nowheat | rs2199477 | T | G | 0.287258 | 0.001642 | 0.000373 | 1.1E-05 | 4.21006E-05 | 19.41107 | 461046 |
| nowheat | rs4898517 | G | A | 0.244803 | -0.00172 | 0.000393 | 1.3E-05 | 4.12397E-05 | 19.0141 | 461046 |
| nowheat | rs2916098 | A | C | 0.120285 | 0.002826 | 0.000545 | 2.1E-07 | 5.84268E-05 | 26.93888 | 461046 |
| nowheat | rs34591731 | T | C | 0.59729 | -0.00149 | 0.000345 | 0.000015 | 4.0584E-05 | 18.71177 | 461046 |
| nowheat | rs6494533 | T | C | 0.257695 | 0.001627 | 0.000385 | 2.4E-05 | 3.86776E-05 | 17.83276 | 461046 |
| nowheat | rs35555308 | T | C | 0.082133 | 0.002603 | 0.000614 | 2.2E-05 | 3.90364E-05 | 17.99818 | 461046 |
| nowheat | rs28474724 | G | A | 0.153312 | 0.002348 | 0.000469 | 5.6E-07 | 5.43483E-05 | 25.05834 | 461046 |
| nowheat | rs8075153 | T | C | 0.445572 | -0.0014 | 0.000339 | 3.8E-05 | 3.67599E-05 | 16.94854 | 461046 |
| nowheat | rs4627401 | T | A | 0.556892 | 0.001393 | 0.000342 | 4.7E-05 | 3.58969E-05 | 16.55066 | 461046 |
| nowheat | rs12603429 | G | C | 0.543689 | -0.00141 | 0.00034 | 3.5E-05 | 3.71307E-05 | 17.11954 | 461046 |
| nowheat | rs41274304 | G | A | 0.054869 | -0.0031 | 0.000759 | 4.5E-05 | 3.61501E-05 | 16.66737 | 461046 |
| nowheat | rs34115485 | G | A | 0.272038 | 0.001549 | 0.000381 | 4.7E-05 | 3.58967E-05 | 16.55057 | 461046 |
| nowheat | rs674009 | A | G | 0.21296 | -0.00168 | 0.000412 | 4.7E-05 | 3.5908E-05 | 16.55575 | 461046 |
| nowheat | rs60663504 | C | A | 0.050133 | -0.00325 | 0.000783 | 3.3E-05 | 3.7411E-05 | 17.24878 | 461046 |
| nowheat | rs10422264 | T | G | 0.077596 | 0.002652 | 0.000641 | 3.5E-05 | 3.71864E-05 | 17.1452 | 461046 |
| nowheat | rs11666436 | T | G | 0.114152 | -0.00226 | 0.000555 | 4.8E-05 | 3.588E-05 | 16.54284 | 461046 |

R^2^ refers to the proportion of variance explained for the association between the SNPs and the exposure variable. The calculation formula is R^2^ = 2*β^2^*EAF*(1-EAF)/(2*β^2^*EAF*(1-EAF) + 2*SE^2^*N*EAF*(1-EAF))

F refers to the F-statistic. The calculation formula is F = R^2^*(N-2)/(1-R^2^), where F < 10 indicated a weak instrument variant.

N refers to the sample size of the initial GWAS from which the genetic variants were selected.

Abbreviations: SNPs: single nucleotide polymorphisms; NEA: non-effect allele; EA: effect allele; EAF: effect allele frequency; SE: standard error.

all = Eggs, dairy, wheat, sugar: I eat all of the above

calo = Type of special diet followed: Low calorie

nodairy = Never eat dairy: Dairy or products containing dairy

noegg = Never eat eggs: Eggs or foods containing eggs

gluten = Type of special diet followed: Gluten-free

nosugar = Never eat sugar: Sugar or foods/drinks containing sugar

vege = Type of special diet followed: Vegetarian

nowheat = Never eat wheat: Wheat or products containing wheat

Supplementary Table 4. IVW method and sensitivity analyses for Mendelian randomization analyses of special diets on osteoporosis(self-reported).

| **exposure** | **outcome** | **nsnp** | **method** | **or** | **or_lci95** | **or_uci95** | **pval** |
| --- | --- | --- | --- | --- | --- | --- | --- |
| all | OP | 18 | MR Egger | 0.866195 | 0.7727 | 0.971016928 | 0.025416809 |
| all | OP | 18 | Inverse variance weighted (fixed effects) | 0.949595 | 0.9292 | 0.970428514 | 2.99666E-06 |
| all | OP | 18 | Simple median | 0.958516 | 0.928 | 0.989993643 | 0.010169143 |
| all | OP | 18 | Weighted median | 0.953348 | 0.9251 | 0.982472818 | 0.0018601 |
| calo | OP | 54 | MR Egger | 0.973981 | 0.9376 | 1.011824335 | 0.181093695 |
| calo | OP | 54 | Inverse variance weighted (fixed effects) | 0.99128 | 0.9823 | 1.000389407 | 0.060575251 |
| calo | OP | 54 | Simple median | 0.994848 | 0.9818 | 1.008114573 | 0.444714135 |
| calo | OP | 54 | Weighted median | 0.994886 | 0.9816 | 1.008333023 | 0.454170871 |
| nodairy | OP | 68 | MR Egger | 0.862403 | 0.7044 | 1.055915728 | 0.156513219 |
| nodairy | OP | 68 | Inverse variance weighted (fixed effects) | 0.959345 | 0.9153 | 1.005530555 | 0.083612941 |
| nodairy | OP | 68 | Simple median | 0.951977 | 0.8904 | 1.017838076 | 0.149313699 |
| nodairy | OP | 68 | Weighted median | 0.953283 | 0.8896 | 1.021473066 | 0.17469478 |
| noegg | OP | 78 | MR Egger | 1.035378 | 0.8655 | 1.238651208 | 0.7049045 |
| noegg | OP | 78 | Inverse variance weighted (fixed effects) | 1.035108 | 0.9937 | 1.078210239 | 0.097364079 |
| noegg | OP | 78 | Simple median | 1.018515 | 0.9604 | 1.080095089 | 0.540183179 |
| noegg | OP | 78 | Weighted median | 1.017356 | 0.9572 | 1.081249901 | 0.579797872 |
| gluten | OP | 15 | MR Egger | 1.14021 | 1.0514 | 1.236526747 | 0.007362892 |
| gluten | OP | 15 | Inverse variance weighted (fixed effects) | 1.080087 | 1.0483 | 1.112817621 | 4.23388E-07 |
| gluten | OP | 15 | Simple median | 1.064016 | 1.0114 | 1.119348076 | 0.016442176 |
| gluten | OP | 15 | Weighted median | 1.098708 | 1.0531 | 1.146336756 | 1.37532E-05 |
| nosugar | OP | 13 | MR Egger | 0.964611 | 0.6918 | 1.345017 | 0.835657184 |
| nosugar | OP | 13 | Inverse variance weighted (fixed effects) | 1.036426 | 1.0057 | 1.068073102 | 0.019730318 |
| nosugar | OP | 13 | Simple median | 1.014197 | 0.9703 | 1.060103782 | 0.532551207 |
| nosugar | OP | 13 | Weighted median | 1.009377 | 0.9687 | 1.051722317 | 0.656209134 |
| vege | OP | 37 | MR Egger | 1.428881 | 1.144 | 1.784715314 | 0.003373268 |
| vege | OP | 37 | Inverse variance weighted (fixed effects) | 0.996651 | 0.977 | 1.01670234 | 0.74133913 |
| vege | OP | 37 | Simple median | 0.985939 | 0.9576 | 1.015084673 | 0.340734963 |
| vege | OP | 37 | Weighted median | 0.986137 | 0.9582 | 1.014835483 | 0.340176789 |
| nowheat | OP | 56 | MR Egger | 1.110543 | 1.0347 | 1.19192409 | 0.005299859 |
| nowheat | OP | 56 | Inverse variance weighted (multiplicative random effects) | 1.053346 | 1.009 | 1.099678496 | 0.017962452 |
| nowheat | OP | 56 | Inverse variance weighted (fixed effects) | 1.053346 | 1.0188 | 1.089024892 | 0.002228433 |
| nowheat | OP | 56 | Simple median | 0.997597 | 0.935 | 1.064370534 | 0.941976551 |
| nowheat | OP | 56 | Weighted median | 1.098721 | 1.0431 | 1.157256847 | 0.000377951 |

OP = Non-cancer illness code, self-reported: osteoporosis || id:ukb-b-12141

all = Eggs, dairy, wheat, sugar: I eat all of the above

calo = Type of special diet followed: Low calorie

nodairy = Never eat dairy: Dairy or products containing dairy

noegg = Never eat eggs: Eggs or foods containing eggs

gluten = Type of special diet followed: Gluten-free

nosugar = Never eat sugar: Sugar or foods/drinks containing sugar

vege = Type of special diet followed: Vegetarian

nowheat = Never eat wheat: Wheat or products containing wheat

nsnp = number of single nucleotide polymorphisms

Supplementary Table 5. Using different methods to evaluation the heterogeneity and pleiotropy of special diets on osteoporosis(self-reported).

|  |  |  | **Heterogeneity** | | **Pleiotropy** | | | **MR-PRESSO** | |
| --- | --- | --- | --- | --- | --- | --- | --- | --- | --- |
| **exposure** | **outcome** | **No of SNPs** | **Cochran’s Q statistic^1^** | **P-value** | **MR-Egger intercept^2^** | **SE** | **P-value** | **Global Test^3^** | **P-value** |
| all | op | 18 | 26.33599 | 0.068553 | 0.00060247 | 0.000372 | 0.124964 | 29.22502 | 0.145 |
| calo | op | 54 | 54.74558 | 0.408131 | 0.00017766 | 0.00019 | 0.354887 | 57.33949 | 0.442 |
| nodairy | op | 68 | 68.24927 | 0.434551 | 0.000192863 | 0.000182 | 0.292606 | 70.27436 | 0.426 |
| noegg | op | 78 | 81.58449 | 0.338765 | -4.98E-07 | 0.00017 | 0.997669 | 84.50628 | 0.365 |
| gluten | op | 15 | 15.77357 | 0.327397 | -0.000275164 | 0.000195 | 0.18089 | 18.39335 | 0.44 |
| nosugar | op | 13 | 15.902 | 0.195766 | 0.000367563 | 0.000863 | 0.678424 | 18.63755 | 0.231 |
| vege | op | 37 | 39.54767 | 0.314495 | -0.001706268 | 0.000535 | 0.003013 | 41.76715 | 0.338 |
| nowheat | op | 56 | 91.845 | 0.001348 | -0.000174048 | 9.53E-05 | 0.073479 | 99.17288 | 0.003 |

^1^The Cochran’s Q test is a statistical test for heterogeneity.

^2^The intercept term from the MR-Egger regression method is a statistical test of horizontal pleiotropy.

^3^The MR-PRESSO method detected the existence of outlier IVs that may have horizontal pleiotropy through the global test.

MR-PRESSO = the Mendelian Randomization Pleiotropy RESidual Sum and Outlier

SNPs = single nucleotide polymorphisms

SE = standard error.

op = Non-cancer illness code, self-reported: osteoporosis || id:ukb-b-12141

all = Eggs, dairy, wheat, sugar: I eat all of the above

calo = Type of special diet followed: Low calorie

nodairy = Never eat dairy: Dairy or products containing dairy

noegg = Never eat eggs: Eggs or foods containing eggs

gluten = Type of special diet followed: Gluten-free

nosugar = Never eat sugar: Sugar or foods/drinks containing sugar

vege = Type of special diet followed: Vegetarian

nowheat = Never eat wheat: Wheat or products containing wheat

Supplementary Table 6. IVW method and sensitivity analyses for Mendelian randomization analyses of special diets on osteoporosis(diagnoses).

| **exposure** | **outcome** | **nsnp** | **method** | **or** | **or_lci95** | **or_uci95** | **pval** |
| --- | --- | --- | --- | --- | --- | --- | --- |
| all | Opdia | 14 | MR Egger | 0.909542069 | 0.860630586 | 0.961233297 | 0.005653628 |
| all | Opdia | 14 | Inverse variance weighted (fixed effects) | 0.985526283 | 0.973364182 | 0.997840348 | 0.021377391 |
| all | Opdia | 14 | Simple median | 0.997548157 | 0.978641168 | 1.016820422 | 0.801469346 |
| all | Opdia | 14 | Weighted median | 0.984156819 | 0.96535921 | 1.003320457 | 0.104570286 |
| calo | Opdia | 31 | MR Egger | 1.031653495 | 0.963427857 | 1.104710566 | 0.379364588 |
| calo | Opdia | 31 | Inverse variance weighted (fixed effects) | 1.003878857 | 0.997660869 | 1.010135599 | 0.221994244 |
| calo | Opdia | 31 | Simple median | 1.001239993 | 0.991803167 | 1.010766609 | 0.797575541 |
| calo | Opdia | 31 | Weighted median | 1.001185689 | 0.992298233 | 1.010152745 | 0.794494681 |
| nodairy | Opdia | 29 | MR Egger | 0.868200663 | 0.526185129 | 1.432523176 | 0.584697751 |
| nodairy | Opdia | 29 | Inverse variance weighted (fixed effects) | 1.027063427 | 0.989927646 | 1.065592306 | 0.15525438 |
| nodairy | Opdia | 29 | Simple median | 1.01698223 | 0.96719368 | 1.069333762 | 0.510837347 |
| nodairy | Opdia | 29 | Weighted median | 1.009143257 | 0.960029806 | 1.060769267 | 0.720676597 |
| noegg | Opdia | 37 | MR Egger | 1.179674856 | 0.880406437 | 1.580670821 | 0.27591962 |
| noegg | Opdia | 37 | Inverse variance weighted (fixed effects) | 1.009675959 | 0.979372506 | 1.040917052 | 0.535675585 |
| noegg | Opdia | 37 | Simple median | 0.996867342 | 0.955135912 | 1.040422085 | 0.885655146 |
| noegg | Opdia | 37 | Weighted median | 0.996852185 | 0.954559536 | 1.041018649 | 0.886653753 |
| gluten | Opdia | 15 | MR Egger | 1.053960519 | 1.003028898 | 1.107478336 | 0.057907026 |
| gluten | Opdia | 15 | Inverse variance weighted (fixed effects) | 1.027222977 | 1.011502916 | 1.043187347 | 0.000641113 |
| gluten | Opdia | 15 | Simple median | 1.018207297 | 0.991263561 | 1.045883395 | 0.187270009 |
| gluten | Opdia | 15 | Weighted median | 1.022554625 | 0.997877579 | 1.047841923 | 0.073529526 |
| nosugar | Opdia | 12 | MR Egger | 1.10857287 | 0.919289561 | 1.336829939 | 0.305912999 |
| nosugar | Opdia | 12 | Inverse variance weighted (fixed effects) | 1.016336345 | 1.000110029 | 1.032825925 | 0.048450135 |
| nosugar | Opdia | 12 | Simple median | 1.012368607 | 0.991288855 | 1.033896619 | 0.252196843 |
| nosugar | Opdia | 12 | Weighted median | 1.011600654 | 0.990064254 | 1.033605524 | 0.293480462 |
| vege | Opdia | 36 | MR Egger | 1.10776158 | 0.976905912 | 1.256145246 | 0.119811103 |
| vege | Opdia | 36 | Inverse variance weighted (fixed effects) | 0.994688558 | 0.984351573 | 1.005134094 | 0.317699108 |
| vege | Opdia | 36 | Simple median | 1.00648199 | 0.991978674 | 1.021197353 | 0.382951086 |
| vege | Opdia | 36 | Weighted median | 1.007057613 | 0.991932098 | 1.022413769 | 0.362373196 |
| nowheat | Opdia | 35 | MR Egger | 1.090482223 | 1.036424678 | 1.147359286 | 0.002094152 |
| nowheat | Opdia | 35 | Inverse variance weighted (fixed effects) | 1.028002623 | 1.003283322 | 1.053330968 | 0.026151015 |
| nowheat | Opdia | 35 | Simple median | 1.005452627 | 0.96555617 | 1.046997591 | 0.792368628 |
| nowheat | Opdia | 35 | Weighted median | 1.045166669 | 1.007257943 | 1.084502112 | 0.019095549 |

Opdia = Diagnoses - secondary ICD10: M81.99 Osteoporosis, unspecified (Site unspecified) || id:ukb-b-17796

all = Eggs, dairy, wheat, sugar: I eat all of the above

calo = Type of special diet followed: Low calorie

nodairy = Never eat dairy: Dairy or products containing dairy

noegg = Never eat eggs: Eggs or foods containing eggs

gluten = Type of special diet followed: Gluten-free

nosugar = Never eat sugar: Sugar or foods/drinks containing sugar

vege = Type of special diet followed: Vegetarian

nowheat = Never eat wheat: Wheat or products containing wheat

nsnp = number of single nucleotide polymorphisms

Supplementary Table 7. Using different methods to evaluation the heterogeneity and pleiotropy of special diets on osteoporosis(diagnoses).

|  |  |  | **Heterogeneity** | | **Pleiotropy** | | | **MR-PRESSO** | |
| --- | --- | --- | --- | --- | --- | --- | --- | --- | --- |
| **exposure** | **outcome** | **No of SNPs** | **Cochran’s Q statistic^1^** | **P-value** | **MR-Egger intercept^2^** | **SE** | **P-value** | **Global Test^3^** | **P-value** |
| all | opdia | 14 | 20.14707 | 0.091603 | 0.000507933 | 0.000174 | 0.012849 | 27.33342 | 0.061 |
| calo | opdia | 31 | 28.66122 | 0.535441 | -0.000231896 | 0.000295 | 0.438793 | 31.33345 | 0.604 |
| nodairy | opdia | 29 | 17.11135 | 0.946357 | 0.000244574 | 0.000371 | 0.515177 | 18.30527 | 0.957 |
| noegg | opdia | 37 | 35.23503 | 0.504783 | -0.000245813 | 0.000235 | 0.301813 | 37.95371 | 0.555 |
| gluten | opdia | 15 | 20.88385 | 0.104646 | -0.000130523 | 0.000119 | 0.292092 | 28.1929 | 0.09 |
| nosugar | opdia | 12 | 8.345087 | 0.682099 | -0.000436667 | 0.000478 | 0.382841 | 9.851657 | 0.685 |
| vege | opdia | 36 | 35.56713 | 0.441525 | -0.0005068 | 0.000301 | 0.101233 | 37.59186 | 0.448 |
| nowheat | opdia | 35 | 35.53164 | 0.396013 | -0.000139781 | 5.40E-05 | 0.014152 | 43.36123 | 0.315 |

^1^The Cochran’s Q test is a statistical test for heterogeneity.

^2^The intercept term from the MR-Egger regression method is a statistical test of horizontal pleiotropy.

^3^The MR-PRESSO method detected the existence of outlier IVs that may have horizontal pleiotropy through the global test.

MR-PRESSO = the Mendelian Randomization Pleiotropy RESidual Sum and Outlier

SNPs = single nucleotide polymorphisms

SE = standard error.

opdia = Diagnoses - secondary ICD10: M81.99 Osteoporosis, unspecified (Site unspecified) || id:ukb-b-17796

all = Eggs, dairy, wheat, sugar: I eat all of the above

calo = Type of special diet followed: Low calorie

nodairy = Never eat dairy: Dairy or products containing dairy

noegg = Never eat eggs: Eggs or foods containing eggs

gluten = Type of special diet followed: Gluten-free

nosugar = Never eat sugar: Sugar or foods/drinks containing sugar

vege = Type of special diet followed: Vegetarian

nowheat = Never eat wheat: Wheat or products containing wheat

Supplementary Table 8. IVW method and sensitivity analyses for Mendelian randomization analyses of special diets on Ultradistal forearm bone mineral density.

| **exposure** | **outcome** | **nsnp** | **method** | **or** | **or_lci95** | **or_uci95** | **pval** |
| --- | --- | --- | --- | --- | --- | --- | --- |
| all | Ultradistal | 15 | MR Egger | 32.63617 | 0.0106 | 100415.8017 | 0.410405251 |
| all | Ultradistal | 15 | Inverse variance weighted (fixed effects) | 3.735925 | 1.312 | 10.63791411 | 0.013562236 |
| all | Ultradistal | 15 | Simple median | 4.927856 | 1.088 | 22.31927455 | 0.038503186 |
| all | Ultradistal | 15 | Weighted median | 5.274093 | 1.2174 | 22.84912936 | 0.026218239 |
| calo | Ultradistal | 44 | MR Egger | 0.80314 | 0.143 | 4.510888664 | 0.804583463 |
| calo | Ultradistal | 44 | Inverse variance weighted (fixed effects) | 1.03511 | 0.6858 | 1.562334103 | 0.869499368 |
| calo | Ultradistal | 44 | Simple median | 1.075068 | 0.5992 | 1.928911074 | 0.808241255 |
| calo | Ultradistal | 44 | Weighted median | 1.082631 | 0.6192 | 1.893016372 | 0.780638195 |
| nodairy | Ultradistal | 62 | MR Egger | 329.1492 | 0.0278 | 3898201.044 | 0.230539632 |
| nodairy | Ultradistal | 62 | Inverse variance weighted (fixed effects) | 1.163043 | 0.1579 | 8.566227892 | 0.882139931 |
| nodairy | Ultradistal | 62 | Simple median | 0.898748 | 0.0464 | 17.42208486 | 0.943731944 |
| nodairy | Ultradistal | 62 | Weighted median | 0.916291 | 0.0467 | 17.96724834 | 0.95408601 |
| noegg | Ultradistal | 65 | MR Egger | 3.019636 | 0.0005 | 19557.93908 | 0.805852296 |
| noegg | Ultradistal | 65 | Inverse variance weighted (fixed effects) | 0.824066 | 0.1305 | 5.205078113 | 0.836968104 |
| noegg | Ultradistal | 65 | Simple median | 2.660403 | 0.1757 | 40.27974545 | 0.480336474 |
| noegg | Ultradistal | 65 | Weighted median | 1.912386 | 0.1337 | 27.36121018 | 0.632939368 |
| gluten | Ultradistal | 11 | MR Egger | 3.24E-11 | 1E-27 | 872146.885 | 0.242370227 |
| gluten | Ultradistal | 11 | Inverse variance weighted (fixed effects) | 5.176209 | 0.8017 | 33.41921894 | 0.084030326 |
| gluten | Ultradistal | 11 | Simple median | 2.687476 | 0.1728 | 41.79489164 | 0.480125581 |
| gluten | Ultradistal | 11 | Weighted median | 2.388775 | 0.1887 | 30.23359792 | 0.501313486 |
| nosugar | Ultradistal | 12 | MR Egger | 7.1E-05 | 9E-10 | 5.532774151 | 0.127430764 |
| nosugar | Ultradistal | 12 | Inverse variance weighted (fixed effects) | 1.060286 | 0.2993 | 3.756296485 | 0.927724918 |
| nosugar | Ultradistal | 12 | Simple median | 1.718228 | 0.3061 | 9.643648114 | 0.5385328 |
| nosugar | Ultradistal | 12 | Weighted median | 1.68557 | 0.3058 | 9.289572521 | 0.548799181 |
| vege | Ultradistal | 34 | MR Egger | 73.18227 | 0.0051 | 1058773.402 | 0.386307892 |
| vege | Ultradistal | 34 | Inverse variance weighted (fixed effects) | 1.383834 | 0.5926 | 3.231630664 | 0.452810834 |
| vege | Ultradistal | 34 | Simple median | 1.688342 | 0.5197 | 5.484373663 | 0.383582419 |
| vege | Ultradistal | 34 | Weighted median | 1.730651 | 0.5341 | 5.608116137 | 0.360515571 |
| nowheat | Ultradistal | 48 | MR Egger | 11.38026 | 0.0005 | 263499.9371 | 0.6375436 |
| nowheat | Ultradistal | 48 | Inverse variance weighted (fixed effects) | 1.132227 | 0.1367 | 9.377883158 | 0.908341705 |
| nowheat | Ultradistal | 48 | Simple median | 0.851224 | 0.0403 | 17.95809211 | 0.917531449 |
| nowheat | Ultradistal | 48 | Weighted median | 0.841116 | 0.0388 | 18.23993981 | 0.912228875 |

Ultradistal = Ultradistal forearm bone mineral density || id:ebi-a-GCST90013422

all = Eggs, dairy, wheat, sugar: I eat all of the above

calo = Type of special diet followed: Low calorie

nodairy = Never eat dairy: Dairy or products containing dairy

noegg = Never eat eggs: Eggs or foods containing eggs

gluten = Type of special diet followed: Gluten-free

nosugar = Never eat sugar: Sugar or foods/drinks containing sugar

vege = Type of special diet followed: Vegetarian

nowheat = Never eat wheat: Wheat or products containing wheat

nsnp = number of single nucleotide polymorphisms

Supplementary Table 9. Using different methods to evaluation the heterogeneity and pleiotropy of special diets on Ultradistal forearm bone mineral density.

|  |  |  | **Heterogeneity** | | **Pleiotropy** | | | **MR-PRESSO** | |
| --- | --- | --- | --- | --- | --- | --- | --- | --- | --- |
| **exposure** | **outcome** | **No of SNPs** | **Cochran’s Q statistic^1^** | **P-value** | **MR-Egger intercept^2^** | **SE** | **P-value** | **Global Test^3^** | **P-value** |
| all | Ultradistal | 15 | 19.67377 | 0.140767 | -0.01278055 | 0.023858 | 0.60121 | 22.43778 | 0.145 |
| calo | Ultradistal | 44 | 33.59521 | 0.847524 | 0.002550801 | 0.008596 | 0.768122 | 46.06867 | 0.492 |
| nodairy | Ultradistal | 62 | 74.71186 | 0.111553 | -0.01023078 | 0.00843 | 0.229653 | 77.10265 | 0.137 |
| noegg | Ultradistal | 65 | 72.565 | 0.216512 | -0.002410901 | 0.008099 | 0.766924 | 77.65039 | 0.215 |
| gluten | Ultradistal | 11 | 10.072459 | 0.434159 | 0.09104622 | 0.068041 | 0.213683 | 12.90338 | 0.483 |
| nosugar | Ultradistal | 12 | 13.12897 | 0.28498 | 0.04919743 | 0.029225 | 0.123207 | 15.58688 | 0.294 |
| vege | Ultradistal | 34 | 28.91779 | 0.67068 | -0.01875249 | 0.023007 | 0.421056 | 30.73414 | 0.648 |
| nowheat | Ultradistal | 48 | 55.66431 | 0.180933 | -0.004287918 | 0.00927 | 0.645862 | 62.1935 | 0.14 |

^1^The Cochran’s Q test is a statistical test for heterogeneity.

^2^The intercept term from the MR-Egger regression method is a statistical test of horizontal pleiotropy.

^3^The MR-PRESSO method detected the existence of outlier IVs that may have horizontal pleiotropy through the global test.

MR-PRESSO = the Mendelian Randomization Pleiotropy RESidual Sum and Outlier

SNPs = single nucleotide polymorphisms

SE = standard error.

Ultradistal = Ultradistal forearm bone mineral density || id:ebi-a-GCST90013422

all = Eggs, dairy, wheat, sugar: I eat all of the above

calo = Type of special diet followed: Low calorie

nodairy = Never eat dairy: Dairy or products containing dairy

noegg = Never eat eggs: Eggs or foods containing eggs

gluten = Type of special diet followed: Gluten-free

nosugar = Never eat sugar: Sugar or foods/drinks containing sugar

vege = Type of special diet followed: Vegetarian

nowheat = Never eat wheat: Wheat or products containing wheat

Supplementary Table 10. IVW method and sensitivity analyses for Mendelian randomization analyses of special diets on Heel bone mineral density.

| **exposure** | **outcome** | **nsnp** | **method** | **or** | **or_lci95** | **or_uci95** | **pval** |
| --- | --- | --- | --- | --- | --- | --- | --- |
| all(MR-PRESSO) | Heel | 11 | MR Egger | 1.03092475 | 0.357648 | 2.97164949 | 0.956265896 |
| all(MR-PRESSO) | Heel | 11 | Inverse variance weighted (multiplicative random effects) | 0.8489598 | 0.655944 | 1.098772198 | 0.213408612 |
| all(MR-PRESSO) | Heel | 11 | Inverse variance weighted (fixed effects) | 0.8489598 | 0.70459 | 1.022910542 | 0.085104381 |
| all(MR-PRESSO) | Heel | 11 | Simple median | 0.90449022 | 0.682231 | 1.199157275 | 0.485367389 |
| all(MR-PRESSO) | Heel | 11 | Weighted median | 0.88037219 | 0.661635 | 1.171424823 | 0.381960324 |
| all | Heel | 17 | MR Egger | 0.97688483 | 0.1299 | 7.346444112 | 0.982174026 |
| all | Heel | 17 | Inverse variance weighted (multiplicative random effects) | 0.80143968 | 0.508252 | 1.263753752 | 0.340800825 |
| all | Heel | 17 | Inverse variance weighted (fixed effects) | 0.80143968 | 0.685281 | 0.937288179 | 0.005593801 |
| all | Heel | 17 | Simple median | 0.90449022 | 0.674475 | 1.212946439 | 0.502532147 |
| all | Heel | 17 | Weighted median | 0.91591567 | 0.684633 | 1.225330284 | 0.554190694 |
| calo(MR-PRESSO) | Heel | 41 | MR Egger | 1.07235904 | 0.762492 | 1.508152988 | 0.690230916 |
| calo(MR-PRESSO) | Heel | 41 | Inverse variance weighted (fixed effects) | 1.02645849 | 0.953144 | 1.105412599 | 0.489747896 |
| calo(MR-PRESSO) | Heel | 41 | Simple median | 1.04924677 | 0.938994 | 1.172445545 | 0.396047419 |
| calo(MR-PRESSO) | Heel | 41 | Weighted median | 1.04654259 | 0.93878 | 1.166674829 | 0.411910048 |
| calo | Heel | 45 | MR Egger | 1.24688728 | 0.63796 | 2.437029833 | 0.522123197 |
| calo | Heel | 45 | Inverse variance weighted (multiplicative random effects) | 1.05487445 | 0.904473 | 1.230285714 | 0.496068845 |
| calo | Heel | 45 | Inverse variance weighted (fixed effects) | 1.05487445 | 0.983239 | 1.1317288 | 0.136512571 |
| calo | Heel | 45 | Simple median | 1.04924677 | 0.944368 | 1.165772655 | 0.370947744 |
| calo | Heel | 45 | Weighted median | 1.04503575 | 0.936272 | 1.166433684 | 0.432086894 |
| nodairy(MR-PRESSO) | Heel | 59 | MR Egger | 0.4574947 | 0.063099 | 3.317027286 | 0.442318657 |
| nodairy(MR-PRESSO) | Heel | 59 | Inverse variance weighted (multiplicative random effects) | 0.91720328 | 0.584367 | 1.43961233 | 0.707090954 |
| nodairy(MR-PRESSO) | Heel | 59 | Inverse variance weighted (fixed effects) | 0.91720328 | 0.64261 | 1.3091321 | 0.633996363 |
| nodairy(MR-PRESSO) | Heel | 59 | Simple median | 1.06866755 | 0.627553 | 1.819846982 | 0.806826748 |
| nodairy(MR-PRESSO) | Heel | 59 | Weighted median | 1.26191707 | 0.753527 | 2.113308126 | 0.376540743 |
| nodairy | Heel | 67 | MR Egger | 0.42803846 | 0.030448 | 6.017311056 | 0.531410504 |
| nodairy | Heel | 67 | Inverse variance weighted (multiplicative random effects) | 0.8595829 | 0.462974 | 1.595949079 | 0.631743528 |
| nodairy | Heel | 67 | Inverse variance weighted (fixed effects) | 0.8595829 | 0.615889 | 1.199701709 | 0.373700042 |
| nodairy | Heel | 67 | Simple median | 1.06866755 | 0.62299 | 1.833176851 | 0.809388587 |
| nodairy | Heel | 67 | Weighted median | 1.34098493 | 0.765811 | 2.348150772 | 0.304653537 |
| noegg(MR-PRESSO) | Heel | 67 | MR Egger | 1.00715783 | 0.137773 | 7.362583852 | 0.99441455 |
| noegg(MR-PRESSO) | Heel | 67 | Inverse variance weighted (multiplicative random effects) | 0.80977904 | 0.506826 | 1.293822048 | 0.377491141 |
| noegg(MR-PRESSO) | Heel | 67 | Inverse variance weighted (fixed effects) | 0.80977904 | 0.594195 | 1.103579787 | 0.181565097 |
| noegg(MR-PRESSO) | Heel | 67 | Simple median | 0.80302138 | 0.484085 | 1.332086872 | 0.395577453 |
| noegg(MR-PRESSO) | Heel | 67 | Weighted median | 0.80658586 | 0.487271 | 1.335151655 | 0.403203375 |
| noegg | Heel | 71 | MR Egger | 1.14775036 | 0.123538 | 10.66339548 | 0.903906444 |
| noegg | Heel | 71 | Inverse variance weighted (multiplicative random effects) | 0.84020624 | 0.499664 | 1.41284185 | 0.511426782 |
| noegg | Heel | 71 | Inverse variance weighted (fixed effects) | 0.84020624 | 0.621671 | 1.135563357 | 0.257283671 |
| noegg | Heel | 71 | Simple median | 0.80302138 | 0.478409 | 1.347892199 | 0.406426721 |
| noegg | Heel | 71 | Weighted median | 0.80646509 | 0.469691 | 1.384708794 | 0.435467522 |
| gluten(MR-PRESSO) | Heel | 7 | MR Egger | 0.01270252 | 0.000472 | 0.341993615 | 0.048329385 |
| gluten(MR-PRESSO) | Heel | 7 | Inverse variance weighted (fixed effects) | 0.76874399 | 0.52171 | 1.132751521 | 0.183599664 |
| gluten(MR-PRESSO) | Heel | 7 | Simple median | 0.88244796 | 0.499041 | 1.560422841 | 0.667191283 |
| gluten(MR-PRESSO) | Heel | 7 | Weighted median | 0.87022991 | 0.513579 | 1.474553647 | 0.60542865 |
| gluten | Heel | 11 | MR Egger | 1.43764771 | 0.026518 | 77.94105887 | 0.862521381 |
| gluten | Heel | 11 | Inverse variance weighted (multiplicative random effects) | 0.56294619 | 0.202264 | 1.566803001 | 0.271251131 |
| gluten | Heel | 11 | Inverse variance weighted (fixed effects) | 0.56294619 | 0.426893 | 0.742361022 | 4.68766E-05 |
| gluten | Heel | 11 | Simple median | 0.88244796 | 0.526957 | 1.477756827 | 0.634499657 |
| gluten | Heel | 11 | Weighted median | 1.19441004 | 0.780656 | 1.827456914 | 0.412920469 |
| nosugar | Heel | 12 | MR Egger | 4.18356178 | 0.234629 | 74.59508874 | 0.353180295 |
| nosugar | Heel | 12 | Inverse variance weighted (multiplicative random effects) | 1.18922513 | 0.865408 | 1.634207605 | 0.285234646 |
| nosugar | Heel | 12 | Inverse variance weighted (fixed effects) | 1.18922513 | 0.955837 | 1.479599463 | 0.119998639 |
| nosugar | Heel | 12 | Simple median | 1.28568681 | 0.938222 | 1.761832542 | 0.117982905 |
| nosugar | Heel | 12 | Weighted median | 1.28506407 | 0.93691 | 1.762591841 | 0.119764593 |
| vege(MR-PRESSO) | Heel | 26 | MR Egger | 1.13444532 | 0.096631 | 13.31832348 | 0.920874489 |
| vege(MR-PRESSO) | Heel | 26 | Inverse variance weighted (multiplicative random effects) | 1.11181135 | 0.897902 | 1.376681054 | 0.330958421 |
| vege(MR-PRESSO) | Heel | 26 | Inverse variance weighted (fixed effects) | 1.11181135 | 0.939964 | 1.315076441 | 0.215990123 |
| vege(MR-PRESSO) | Heel | 26 | Simple median | 1.18650948 | 0.923328 | 1.524707552 | 0.181367212 |
| vege(MR-PRESSO) | Heel | 26 | Weighted median | 1.1818884 | 0.919836 | 1.518597598 | 0.191332215 |
| vege | Heel | 30 | MR Egger | 0.34914963 | 0.004201 | 29.02164556 | 0.644410849 |
| vege | Heel | 30 | Inverse variance weighted (multiplicative random effects) | 1.06464153 | 0.727672 | 1.55765495 | 0.746983257 |
| vege | Heel | 30 | Inverse variance weighted (fixed effects) | 1.06464153 | 0.910204 | 1.245283164 | 0.433420088 |
| vege | Heel | 30 | Simple median | 1.13304578 | 0.879251 | 1.460097642 | 0.334338919 |
| vege | Heel | 30 | Weighted median | 1.14244922 | 0.885318 | 1.474261538 | 0.305984592 |
| nowheat(MR-PRESSO) | Heel | 45 | MR Egger | 0.99558708 | 0.546805 | 1.812699714 | 0.98852531 |
| nowheat(MR-PRESSO) | Heel | 45 | Inverse variance weighted (multiplicative random effects) | 0.74094242 | 0.518129 | 1.059573024 | 0.100398559 |
| nowheat(MR-PRESSO) | Heel | 45 | Inverse variance weighted (fixed effects) | 0.74094242 | 0.563404 | 0.974425838 | 0.031923308 |
| nowheat(MR-PRESSO) | Heel | 45 | Simple median | 0.62689671 | 0.358325 | 1.096768549 | 0.101770355 |
| nowheat(MR-PRESSO) | Heel | 45 | Weighted median | 0.98573707 | 0.666906 | 1.456993044 | 0.942554413 |
| nowheat | Heel | 50 | MR Egger | 0.95726099 | 0.42184 | 2.172265893 | 0.917228501 |
| nowheat | Heel | 50 | Inverse variance weighted (multiplicative random effects) | 0.68316596 | 0.423777 | 1.101323574 | 0.117848532 |
| nowheat | Heel | 50 | Inverse variance weighted (fixed effects) | 0.68316596 | 0.52337 | 0.891750657 | 0.005066598 |
| nowheat | Heel | 50 | Simple median | 0.5824105 | 0.341043 | 0.994603283 | 0.047723932 |
| nowheat | Heel | 50 | Weighted median | 0.98565842 | 0.655759 | 1.48152319 | 0.9446099 |

Heel = Heel bone mineral density || id:ebi-a-GCST006979

all = Eggs, dairy, wheat, sugar: I eat all of the above

calo = Type of special diet followed: Low calorie

nodairy = Never eat dairy: Dairy or products containing dairy

noegg = Never eat eggs: Eggs or foods containing eggs

gluten = Type of special diet followed: Gluten-free

nosugar = Never eat sugar: Sugar or foods/drinks containing sugar

vege = Type of special diet followed: Vegetarian

nowheat = Never eat wheat: Wheat or products containing wheat

all(MR-PRESSO) = “Eggs, dairy, wheat, sugar: I eat all of the above” after removing outliers using MR-PRESSO

calo(MR-PRESSO) = “Type of special diet followed: Low calorie” after removing outliers using MR-PRESSO

nodairy(MR-PRESSO) = “Never eat dairy: Dairy or products containing dairy” after removing outliers using MR-PRESSO

noegg(MR-PRESSO) = “Never eat eggs: Eggs or foods containing eggs” after removing outliers using MR-PRESSO

gluten(MR-PRESSO) = “Type of special diet followed: Gluten-free” after removing outliers using MR-PRESSO

vege(MR-PRESSO) = “Type of special diet followed: Vegetarian” after removing outliers using MR-PRESSO

nowheat(MR-PRESSO) = “Never eat wheat: Wheat or products containing wheat” after removing outliers using MR-PRESSO

nsnp = number of single nucleotide polymorphisms

Supplementary Table 11. Using different methods to evaluation the heterogeneity and pleiotropy of special diets on Heel bone mineral density.

|  |  |  | **Heterogeneity** | | **Pleiotropy** | | | **MR-PRESSO** | |
| --- | --- | --- | --- | --- | --- | --- | --- | --- | --- |
| **exposure** | **outcome** | **No of SNPs** | **Cochran’s Q statistic^1^** | **P-value** | **MR-Egger intercept^2^** | **SE** | **P-value** | **Global Test^3^** | **P-value** |
| all | heel | 17 | 135.3594 | 5.81E-21 | -0.001301824 | 0.006583 | 0.845902 | 149.9194 | <0.001 |
| all(MR-PRESSO) | heel | 11 | 19.14943 | 3.84E-02 | -0.001306172 | 0.003513 | 0.71863 | 22.6259 | 0.076 |
| calo | heel | 45 | 210.5183 | 1.38E-23 | -0.00167853 | 0.003339 | 0.617691 | 221.6968 | <0.001 |
| calo(MR-PRESSO) | heel | 41 | 46.01618 | 2.37E-01 | -0.000436312 | 0.001686 | 0.797208 | 49.81704 | 0.225 |
| nodairy | heel | 67 | 227.3684 | 1.35E-19 | 0.00126762 | 0.002383 | 0.596562 | 234.3567 | <0.001 |
| nodairy(MR-PRESSO) | heel | 59 | 93.11229 | 2.35E-03 | 0.001273145 | 0.001801 | 0.482522 | 96.30823 | 0.004 |
| noegg | heel | 71 | 208.356 | 1.15E-15 | -0.000593194 | 0.002102 | 0.778674 | 216.6611 | <0.001 |
| noegg(MR-PRESSO) | heel | 67 | 151.2405 | 1.22E-08 | -0.000417617 | 0.001888 | 0.825604 | 158.1677 | <0.001 |
| gluten | heel | 11 | 136.8993 | 1.82E-24 | -0.004000952 | 0.008378 | 0.644359 | 163.8857 | <0.001 |
| gluten(MR-PRESSO) | heel | 7 | 6.802742 | 3.39E-01 | 0.01517815 | 0.006172 | 0.057285 | 9.510386 | 0.432 |
| nosugar | heel | 12 | 23.28479 | 0.01611 | -0.006426123 | 0.007462 | 0.409325 | 27.65235 | 0.019 |
| vege | heel | 30 | 170.9743 | 4.61E-22 | 0.005191532 | 0.010462 | 0.623595 | 182.6748 | <0.001 |
| vege(MR-PRESSO) | heel | 26 | 40.49159 | 2.59E-02 | -9.42E-05 | 0.005848 | 0.987287 | 43.73295 | 0.021 |
| nowheat | heel | 50 | 157.3875 | 2.68E-13 | -0.001013293 | 0.00102 | 0.325716 | 185.3822 | <0.001 |
| nowheat(MR-PRESSO) | heel | 45 | 75.02771 | 0.002441 | -0.000934088 | 0.000778 | 0.236318 | 81.18582 | 0.011 |

^1^The Cochran’s Q test is a statistical test for heterogeneity.

^2^The intercept term from the MR-Egger regression method is a statistical test of horizontal pleiotropy.

^3^The MR-PRESSO method detected the existence of outlier IVs that may have horizontal pleiotropy through the global test.

MR-PRESSO = the Mendelian Randomization Pleiotropy RESidual Sum and Outlier

SNPs = single nucleotide polymorphisms

SE = standard error.

heel = Heel bone mineral density || id:ebi-a-GCST006979

all = Eggs, dairy, wheat, sugar: I eat all of the above

calo = Type of special diet followed: Low calorie

nodairy = Never eat dairy: Dairy or products containing dairy

noegg = Never eat eggs: Eggs or foods containing eggs

gluten = Type of special diet followed: Gluten-free

nosugar = Never eat sugar: Sugar or foods/drinks containing sugar

vege = Type of special diet followed: Vegetarian

nowheat = Never eat wheat: Wheat or products containing wheat

all(MR-PRESSO) = “Eggs, dairy, wheat, sugar: I eat all of the above” after removing outliers using MR-PRESSO

calo(MR-PRESSO) = “Type of special diet followed: Low calorie” after removing outliers using MR-PRESSO

nodairy(MR-PRESSO) = “Never eat dairy: Dairy or products containing dairy” after removing outliers using MR-PRESSO

noegg(MR-PRESSO) = “Never eat eggs: Eggs or foods containing eggs” after removing outliers using MR-PRESSO

gluten(MR-PRESSO) = “Type of special diet followed: Gluten-free” after removing outliers using MR-PRESSO

vege(MR-PRESSO) = “Type of special diet followed: Vegetarian” after removing outliers using MR-PRESSO

nowheat(MR-PRESSO) = “Never eat wheat: Wheat or products containing wheat” after removing outliers using MR-PRESSO

Supplementary Table 12. IVW method and sensitivity analyses for Mendelian randomization analyses of special diets on Total body bone mineral density.

| **exposure** | **outcome** | **nsnp** | **method** | **or** | **or_lci95** | **or_uci95** | **pval** |
| --- | --- | --- | --- | --- | --- | --- | --- |
| all | total | 17 | MR Egger | 0.331563 | 0.0093 | 11.79430441 | 0.55369212 |
| all | total | 17 | Inverse variance weighted (fixed effects) | 0.976433 | 0.5686 | 1.676705979 | 0.931106245 |
| all | total | 17 | Simple median | 0.814658 | 0.3764 | 1.762968889 | 0.602754694 |
| all | total | 17 | Weighted median | 0.826298 | 0.3706 | 1.842104408 | 0.640884306 |
| calo | total | 54 | MR Egger | 0.594975 | 0.2697 | 1.312467701 | 0.204013219 |
| calo | total | 54 | Inverse variance weighted (fixed effects) | 0.922665 | 0.7533 | 1.130160497 | 0.436736622 |
| calo | total | 54 | Simple median | 0.862526 | 0.661 | 1.125577881 | 0.276175208 |
| calo | total | 54 | Weighted median | 0.826706 | 0.6242 | 1.094960377 | 0.18441406 |
| nodairy | total | 67 | MR Egger | 55.7832 | 0.4981 | 6246.987977 | 0.099628141 |
| nodairy | total | 67 | Inverse variance weighted (fixed effects) | 0.504644 | 0.1731 | 1.470923917 | 0.210206617 |
| nodairy | total | 67 | Simple median | 0.466175 | 0.0994 | 2.185924487 | 0.333020272 |
| nodairy | total | 67 | Weighted median | 0.540596 | 0.1062 | 2.752453205 | 0.458868785 |
| noegg(MR-PRESSO) | total | 77 | MR Egger | 0.351006 | 0.0043 | 28.82848841 | 0.642929621 |
| noegg(MR-PRESSO) | total | 77 | Inverse variance weighted (fixed effects) | 0.575494 | 0.2295 | 1.443016972 | 0.23877016 |
| noegg(MR-PRESSO) | total | 77 | Simple median | 0.740441 | 0.1824 | 3.006542727 | 0.674249449 |
| noegg(MR-PRESSO) | total | 77 | Weighted median | 0.81027 | 0.2034 | 3.228057273 | 0.765458115 |
| noegg | total | 78 | MR Egger | 0.494682 | 0.0049 | 49.89509861 | 0.765754215 |
| noegg | total | 78 | Inverse variance weighted (multiplicative random effects) | 0.672138 | 0.2337 | 1.932869096 | 0.461006912 |
| noegg | total | 78 | Inverse variance weighted (fixed effects) | 0.672138 | 0.2694 | 1.677180843 | 0.394446218 |
| noegg | total | 78 | Simple median | 0.781478 | 0.2013 | 3.034155715 | 0.721642163 |
| noegg | total | 78 | Weighted median | 0.833646 | 0.2045 | 3.398879158 | 0.799690289 |
| gluten(MR-PRESSO) | total | 13 | MR Egger | 0.610207 | 0.0274 | 13.6042682 | 0.760970013 |
| gluten(MR-PRESSO) | total | 13 | Inverse variance weighted (fixed effects) | 0.734746 | 0.3252 | 1.660134405 | 0.458602962 |
| gluten(MR-PRESSO) | total | 13 | Simple median | 0.720111 | 0.2025 | 2.561404739 | 0.612026938 |
| gluten(MR-PRESSO) | total | 13 | Weighted median | 0.708282 | 0.2184 | 2.297248953 | 0.565596313 |
| gluten | total | 14 | MR Egger | 0.97649 | 0.0122 | 77.95678524 | 0.991680486 |
| gluten | total | 14 | Inverse variance weighted (multiplicative random effects) | 0.473224 | 0.1189 | 1.883233848 | 0.288355522 |
| gluten | total | 14 | Inverse variance weighted (fixed effects) | 0.473224 | 0.214 | 1.046508494 | 0.064641008 |
| gluten | total | 14 | Simple median | 0.650632 | 0.2061 | 2.05354634 | 0.463593275 |
| gluten | total | 14 | Weighted median | 0.693164 | 0.2247 | 2.137966408 | 0.523640681 |
| nosugar | total | 12 | MR Egger | 0.898376 | 0.0017 | 471.8198712 | 0.973909014 |
| nosugar | total | 12 | Inverse variance weighted (fixed effects) | 1.554325 | 0.7867 | 3.070906219 | 0.204263969 |
| nosugar | total | 12 | Simple median | 1.43868 | 0.5967 | 3.468904795 | 0.417932283 |
| nosugar | total | 12 | Weighted median | 1.450027 | 0.5749 | 3.657191958 | 0.431131455 |
| vege | total | 37 | MR Egger | 0.01223 | 5E-05 | 3.01065685 | 0.125957696 |
| vege | total | 37 | Inverse variance weighted (fixed effects) | 0.942142 | 0.6017 | 1.475150641 | 0.794450209 |
| vege | total | 37 | Simple median | 1.2476 | 0.6488 | 2.39904582 | 0.50724083 |
| vege | total | 37 | Weighted median | 1.217111 | 0.6347 | 2.333926852 | 0.55419463 |
| nowheat | total | 56 | MR Egger | 4.486896 | 0.5654 | 35.60698402 | 0.161227464 |
| nowheat | total | 56 | Inverse variance weighted (fixed effects) | 1.983214 | 0.7884 | 4.989067365 | 0.145739358 |
| nowheat | total | 56 | Simple median | 2.526011 | 0.6212 | 10.2720895 | 0.195417478 |
| nowheat | total | 56 | Weighted median | 2.91039 | 0.5765 | 14.69185082 | 0.195910985 |

total = Total body bone mineral density || id:ebi-a-GCST005348

all = Eggs, dairy, wheat, sugar: I eat all of the above

calo = Type of special diet followed: Low calorie

nodairy = Never eat dairy: Dairy or products containing dairy

noegg = Never eat eggs: Eggs or foods containing eggs

gluten = Type of special diet followed: Gluten-free

nosugar = Never eat sugar: Sugar or foods/drinks containing sugar

vege = Type of special diet followed: Vegetarian

nowheat = Never eat wheat: Wheat or products containing wheat

noegg(MR-PRESSO) = “Never eat eggs: Eggs or foods containing eggs” after removing outliers using MR-PRESSO

gluten(MR-PRESSO) = “Type of special diet followed: Gluten-free” after removing outliers using MR-PRESSO

nsnp = number of single nucleotide polymorphisms

Supplementary Table 13. Using different methods to evaluation the heterogeneity and pleiotropy of special diets on Total body bone mineral density.

|  |  |  | **Heterogeneity** | | **Pleiotropy** | | | **MR-PRESSO** | |
| --- | --- | --- | --- | --- | --- | --- | --- | --- | --- |
| **exposure** | **outcome** | **No of SNPs** | **Cochran’s Q statistic^1^** | **P-value** | **MR-Egger intercept^2^** | **SE** | **P-value** | **Global Test^3^** | **P-value** |
| all | total | 17 | 22.2072 | 0.136626 | 0.006546367 | 0.01086 | 0.555647 | 24.92158 | 0.182 |
| calo | total | 54 | 41.95077 | 0.862671 | 0.004482915 | 0.003986 | 0.265943 | 44.42836 | 0.911 |
| nodairy | total | 67 | 73.54123 | 0.244952 | -0.008491204 | 0.004224 | 0.04854 | 75.60161 | 0.239 |
| noegg | total | 78 | 102.7507 | 0.026626 | 0.000578664 | 0.004324 | 0.893893 | 111.2849 | 0.021 |
| noegg(MR-PRESSO) | total | 77 | 92.35638 | 0.097641 | 0.000932642 | 0.004128 | 0.821849 | 100.719 | 0.067 |
| gluten | total | 14 | 39.3721 | 0.000174 | -0.003254519 | 0.009489 | 0.737552 | 44.27795 | 0.001 |
| gluten(MR-PRESSO) | total | 13 | 17.858 | 0.120071 | 0.000845848 | 0.006798 | 0.903226 | 20.77892 | 0.168 |
| nosugar | total | 12 | 10.74122 | 0.465191 | 0.00280469 | 0.016246 | 0.866382 | 12.85978 | 0.49 |
| vege | total | 37 | 45.83908 | 0.126079 | 0.02056439 | 0.013244 | 0.129475 | 48.36938 | 0.111 |
| nowheat | total | 56 | 67.58623 | 0.11869 | -0.002038522 | 0.002293 | 0.377996 | 76.70495 | 0.094 |

^1^The Cochran’s Q test is a statistical test for heterogeneity.

^2^The intercept term from the MR-Egger regression method is a statistical test of horizontal pleiotropy.

^3^The MR-PRESSO method detected the existence of outlier IVs that may have horizontal pleiotropy through the global test.

MR-PRESSO = the Mendelian Randomization Pleiotropy RESidual Sum and Outlier

SNPs = single nucleotide polymorphisms

SE = standard error.

total = Total body bone mineral density || id:ebi-a-GCST005348

all = Eggs, dairy, wheat, sugar: I eat all of the above

calo = Type of special diet followed: Low calorie

nodairy = Never eat dairy: Dairy or products containing dairy

noegg = Never eat eggs: Eggs or foods containing eggs

gluten = Type of special diet followed: Gluten-free

nosugar = Never eat sugar: Sugar or foods/drinks containing sugar

vege = Type of special diet followed: Vegetarian

nowheat = Never eat wheat: Wheat or products containing wheat

noegg(MR-PRESSO) = “Never eat eggs: Eggs or foods containing eggs” after removing outliers using MR-PRESSO

gluten(MR-PRESSO) = “Type of special diet followed: Gluten-free” after removing outliers using MR-PRESSO

Supplementary Table 14. IVW method and sensitivity analyses for Mendelian randomization analyses of special diets on Femoral neck bone mineral density.

| **exposure** | **outcome** | **nsnp** | **method** | **or** | **or_lci95** | **or_uci95** | **pval** |
| --- | --- | --- | --- | --- | --- | --- | --- |
| all | femoral | 12 | MR Egger | 0.1597 | 0.00201777 | 12.63966295 | 0.42995 |
| all | femoral | 12 | Inverse variance weighted (fixed effects) | 1.6164 | 0.735156876 | 3.554078894 | 0.232238 |
| all | femoral | 12 | Simple median | 2.0635 | 0.649071634 | 6.560421985 | 0.219603 |
| all | femoral | 12 | Weighted median | 1.7691 | 0.571712597 | 5.474193391 | 0.322251 |
| calo | femoral | 47 | MR Egger | 0.3989 | 0.114215556 | 1.393456761 | 0.156772 |
| calo | femoral | 47 | Inverse variance weighted (fixed effects) | 0.8894 | 0.666831797 | 1.186324529 | 0.425242 |
| calo | femoral | 47 | Simple median | 0.9155 | 0.609522741 | 1.375036823 | 0.670505 |
| calo | femoral | 47 | Weighted median | 0.8926 | 0.597464574 | 1.333385835 | 0.57886 |
| nodairy | femoral | 63 | MR Egger | 0.096 | 0.000203771 | 45.22199273 | 0.458384 |
| nodairy | femoral | 63 | Inverse variance weighted (fixed effects) | 1.5404 | 0.369481381 | 6.422225693 | 0.553089 |
| nodairy | femoral | 63 | Simple median | 2.8752 | 0.357660133 | 23.11381763 | 0.320639 |
| nodairy | femoral | 63 | Weighted median | 2.8764 | 0.386889463 | 21.3846078 | 0.301966 |
| noegg | femoral | 73 | MR Egger | 0.0873 | 0.00035782 | 21.31419406 | 0.387645 |
| noegg | femoral | 73 | Inverse variance weighted (fixed effects) | 0.334 | 0.097518367 | 1.144246854 | 0.080899 |
| noegg | femoral | 73 | Simple median | 0.3135 | 0.053595041 | 1.833678102 | 0.198029 |
| noegg | femoral | 73 | Weighted median | 0.2614 | 0.049118306 | 1.391229457 | 0.115741 |
| gluten | femoral | 13 | MR Egger | 4.417 | 0.013885545 | 1405.049927 | 0.623345 |
| gluten | femoral | 13 | Inverse variance weighted (fixed effects) | 1.1008 | 0.34415613 | 3.520735746 | 0.871429 |
| gluten | femoral | 13 | Simple median | 0.8479 | 0.175955725 | 4.08588175 | 0.837067 |
| gluten | femoral | 13 | Weighted median | 0.8774 | 0.18686074 | 4.119559514 | 0.868319 |
| nosugar | femoral | 12 | MR Egger | 0.2044 | 7.02823E-05 | 594.4885123 | 0.704589 |
| nosugar | femoral | 12 | Inverse variance weighted (fixed effects) | 1.0441 | 0.424870809 | 2.565900328 | 0.925027 |
| nosugar | femoral | 12 | Simple median | 0.9757 | 0.286506517 | 3.322845552 | 0.968631 |
| nosugar | femoral | 12 | Weighted median | 0.9982 | 0.299944957 | 3.322009551 | 0.997668 |
| vege | femoral | 30 | MR Egger | 0.0085 | 1.32498E-06 | 55.01966893 | 0.296233 |
| vege | femoral | 30 | Inverse variance weighted (fixed effects) | 1.1537 | 0.60731509 | 2.19155649 | 0.662359 |
| vege | femoral | 30 | Simple median | 1.0956 | 0.42938056 | 2.79527654 | 0.848555 |
| vege | femoral | 30 | Weighted median | 0.9997 | 0.382240611 | 2.614722615 | 0.999555 |
| nowheat | femoral | 51 | MR Egger | 2.5758 | 0.182583529 | 36.33802587 | 0.486822 |
| nowheat | femoral | 51 | Inverse variance weighted (fixed effects) | 2.5129 | 0.72890187 | 8.662934182 | 0.144505 |
| nowheat | femoral | 51 | Simple median | 3.0306 | 0.431600096 | 21.280191 | 0.264847 |
| nowheat | femoral | 51 | Weighted median | 4.8689 | 0.600074532 | 39.50606377 | 0.138371 |

femoral = Femoral neck bone mineral density || id:ieu-a-980

all = Eggs, dairy, wheat, sugar: I eat all of the above

calo = Type of special diet followed: Low calorie

nodairy = Never eat dairy: Dairy or products containing dairy

noegg = Never eat eggs: Eggs or foods containing eggs

gluten = Type of special diet followed: Gluten-free

nosugar = Never eat sugar: Sugar or foods/drinks containing sugar

vege = Type of special diet followed: Vegetarian

nowheat = Never eat wheat: Wheat or products containing wheat

nsnp = number of single nucleotide polymorphisms

Supplementary Table 15. Using different methods to evaluation the heterogeneity and pleiotropy of special diets on Femoral neck bone mineral density.

|  |  |  | **Heterogeneity** | | **Pleiotropy** | | | **MR-PRESSO** | |
| --- | --- | --- | --- | --- | --- | --- | --- | --- | --- |
| **exposure** | **outcome** | **No of SNPs** | **Cochran’s Q statistic^1^** | **P-value** | **MR-Egger intercept^2^** | **SE** | **P-value** | **Global Test^3^** | **P-value** |
| all | femoral | 12 | 12.35451 | 0.337594 | 0.01445199 | 0.013671 | 0.31533 | 15.34192 | 0.332 |
| calo | femoral | 47 | 48.45023 | 0.37436 | 0.008009427 | 0.006197 | 0.202776 | 51.56169 | 0.421 |
| nodairy | femoral | 63 | 56.26063 | 0.681481 | 0.005093739 | 0.005606 | 0.367131 | 58.06671 | 0.698 |
| noegg | femoral | 73 | 58.44163 | 0.875596 | 0.002496466 | 0.005087 | 0.625097 | 60.83462 | 0.912 |
| gluten | femoral | 13 | 6.826472 | 0.868865 | -0.005390727 | 0.011172 | 0.638888 | 8.183911 | 0.909 |
| nosugar | femoral | 12 | 9.038183 | 0.618368 | 0.008367195 | 0.020744 | 0.695176 | 10.84458 | 0.621 |
| vege | femoral | 30 | 42.44615 | 0.051176 | 0.02288 | 0.020788 | 0.280422 | 45.39124 | 0.06 |
| nowheat | femoral | 51 | 53.38769 | 0.3454 | -6.05E-05 | 0.002883 | 0.983338 | 57.01365 | 0.367 |

^1^The Cochran’s Q test is a statistical test for heterogeneity.

^2^The intercept term from the MR-Egger regression method is a statistical test of horizontal pleiotropy.

^3^The MR-PRESSO method detected the existence of outlier IVs that may have horizontal pleiotropy through the global test.

MR-PRESSO = the Mendelian Randomization Pleiotropy RESidual Sum and Outlier

SNPs = single nucleotide polymorphisms

SE = standard error.

femoral = Femoral neck bone mineral density || id:ieu-a-980

all = Eggs, dairy, wheat, sugar: I eat all of the above

calo = Type of special diet followed: Low calorie

nodairy = Never eat dairy: Dairy or products containing dairy

noegg = Never eat eggs: Eggs or foods containing eggs

gluten = Type of special diet followed: Gluten-free

nosugar = Never eat sugar: Sugar or foods/drinks containing sugar

vege = Type of special diet followed: Vegetarian

nowheat = Never eat wheat: Wheat or products containing wheat

Supplementary Table 16. IVW method and sensitivity analyses for Mendelian randomization analyses of special diets on Lumbar spine bone mineral density.

| **exposure** | **outcome** | **nsnp** | **method** | **or** | **or_lci95** | **or_uci95** | **pval** |
| --- | --- | --- | --- | --- | --- | --- | --- |
| all | lumbar | 12 | MR Egger | 4.175681 | 0.039 | 446.5749479 | 0.562131417 |
| all | lumbar | 12 | Inverse variance weighted (fixed effects) | 0.889081 | 0.3577 | 2.209802082 | 0.800198272 |
| all | lumbar | 12 | Simple median | 1.210254 | 0.3826 | 3.827961857 | 0.745319782 |
| all | lumbar | 12 | Weighted median | 1.123791 | 0.3233 | 3.905973229 | 0.854315836 |
| calo | lumbar | 47 | MR Egger | 0.523501 | 0.1214 | 2.258285521 | 0.390117947 |
| calo | lumbar | 47 | Inverse variance weighted (fixed effects) | 0.872899 | 0.6256 | 1.218002689 | 0.423857189 |
| calo | lumbar | 47 | Simple median | 0.730696 | 0.4423 | 1.207244447 | 0.22065415 |
| calo | lumbar | 47 | Weighted median | 0.769157 | 0.4699 | 1.259111209 | 0.296608116 |
| nodairy | lumbar | 63 | MR Egger | 3.583065 | 0.0028 | 4641.817721 | 0.728267135 |
| nodairy | lumbar | 63 | Inverse variance weighted (fixed effects) | 0.760535 | 0.1442 | 4.010895536 | 0.746945569 |
| nodairy | lumbar | 63 | Simple median | 0.305602 | 0.0257 | 3.632387991 | 0.347905318 |
| nodairy | lumbar | 63 | Weighted median | 0.288901 | 0.0267 | 3.125144082 | 0.306753102 |
| no | lumbar | 73 | MR Egger | 9.860902 | 0.0103 | 9455.338115 | 0.515653129 |
| noegg | lumbar | 73 | Inverse variance weighted (fixed effects) | 0.349992 | 0.0838 | 1.462409806 | 0.150145437 |
| noegg | lumbar | 73 | Simple median | 0.339585 | 0.0447 | 2.580501044 | 0.296573411 |
| noegg | lumbar | 73 | Weighted median | 0.343696 | 0.0433 | 2.728891619 | 0.312341715 |
| gluten | lumbar | 13 | MR Egger | 148.3963 | 0.0573 | 384160.9518 | 0.238309604 |
| gluten | lumbar | 13 | Inverse variance weighted (fixed effects) | 1.647273 | 0.4301 | 6.309678379 | 0.466340441 |
| gluten | lumbar | 13 | Simple median | 1.713096 | 0.2404 | 12.20971207 | 0.591111881 |
| gluten | lumbar | 13 | Weighted median | 2.010305 | 0.2988 | 13.52469307 | 0.472767577 |
| nosugar | lumbar | 12 | MR Egger | 0.036227 | 2E-07 | 7539.022752 | 0.606973485 |
| nosugar | lumbar | 12 | Inverse variance weighted (fixed effects) | 0.807485 | 0.2838 | 2.297472192 | 0.688556641 |
| nosugar | lumbar | 12 | Simple median | 0.622594 | 0.1362 | 2.845507712 | 0.541073454 |
| nosugar | lumbar | 12 | Weighted median | 0.616977 | 0.1351 | 2.816791536 | 0.533072084 |
| vege | lumbar | 30 | MR Egger | 0.154783 | 3E-05 | 828.6989144 | 0.673417235 |
| vege | lumbar | 30 | Inverse variance weighted (fixed effects) | 0.862318 | 0.408 | 1.822543593 | 0.698043548 |
| vege | lumbar | 30 | Simple median | 0.940111 | 0.3222 | 2.74273785 | 0.909990437 |
| vege | lumbar | 30 | Weighted median | 0.958818 | 0.3316 | 2.772217374 | 0.938118471 |
| nowheat | lumbar | 51 | MR Egger | 8.043055 | 0.4387 | 147.4722167 | 0.16639884 |
| nowheat | lumbar | 51 | Inverse variance weighted (fixed effects) | 2.303013 | 0.5528 | 9.594894812 | 0.251878786 |
| nowheat | lumbar | 51 | Simple median | 0.783393 | 0.0728 | 8.427450813 | 0.840376635 |
| nowheat | lumbar | 51 | Weighted median | 6.029532 | 0.6114 | 59.46163595 | 0.123887383 |

lumbar = Lumbar spine bone mineral density || id:ieu-a-982

all = Eggs, dairy, wheat, sugar: I eat all of the above

calo = Type of special diet followed: Low calorie

nodairy = Never eat dairy: Dairy or products containing dairy

noegg = Never eat eggs: Eggs or foods containing eggs

gluten = Type of special diet followed: Gluten-free

nosugar = Never eat sugar: Sugar or foods/drinks containing sugar

vege = Type of special diet followed: Vegetarian

nowheat = Never eat wheat: Wheat or products containing wheat

nsnp = number of single nucleotide polymorphisms

Supplementary Table 17. Using different methods to evaluation the heterogeneity and pleiotropy of special diets on Lumbar spine bone mineral density.

|  |  |  | Heterogeneity | | Pleiotropy | | | MR-PRESSO | |
| --- | --- | --- | --- | --- | --- | --- | --- | --- | --- |
| exposure | outcome | No of SNPs | Cochran’s Q statistic^1^ | P-value | MR-Egger intercept^2^ | SE | P-value | Global Test^3^ | P-value |
| all | lumbar | 12 | 6.064109 | 0.869053 | -0.009718404 | 0.01469 | 0.523198 | 7.190248 | 0.89 |
| calo | lumbar | 47 | 47.79848 | 0.399567 | 0.00509554 | 0.007227 | 0.484428 | 51.07943 | 0.453 |
| nodairy | lumbar | 63 | 57.97628 | 0.621424 | -0.002845906 | 0.006531 | 0.66453 | 59.86779 | 0.609 |
| noegg | lumbar | 73 | 82.6756 | 0.182984 | -0.006208794 | 0.00635 | 0.331537 | 85.03829 | 0.229 |
| gluten | lumbar | 13 | 17.72027 | 0.124452 | -0.01754127 | 0.015295 | 0.275784 | 20.63027 | 0.184 |
| nosugar | lumbar | 12 | 17.72954 | 0.088072 | 0.01592796 | 0.031856 | 0.627898 | 21.10158 | 0.096 |
| vege | lumbar | 30 | 28.78532 | 0.476288 | 0.008012972 | 0.020356 | 0.696827 | 30.7408 | 0.484 |
| nowheat | lumbar | 51 | 51.81978 | 0.402707 | -0.003117078 | 0.003204 | 0.335419 | 59.63757 | 0.3 |

^1^The Cochran’s Q test is a statistical test for heterogeneity.

^2^The intercept term from the MR-Egger regression method is a statistical test of horizontal pleiotropy.

^3^The MR-PRESSO method detected the existence of outlier IVs that may have horizontal pleiotropy through the global test.

MR-PRESSO = the Mendelian Randomization Pleiotropy RESidual Sum and Outlier

SNPs = single nucleotide polymorphisms

SE = standard error.

lumbar = Lumbar spine bone mineral density || id:ieu-a-982

all = Eggs, dairy, wheat, sugar: I eat all of the above

calo = Type of special diet followed: Low calorie

nodairy = Never eat dairy: Dairy or products containing dairy

noegg = Never eat eggs: Eggs or foods containing eggs

gluten = Type of special diet followed: Gluten-free

nosugar = Never eat sugar: Sugar or foods/drinks containing sugar

vege = Type of special diet followed: Vegetarian

nowheat = Never eat wheat: Wheat or products containing wheat

nsnp = number of single nucleotide polymorphisms
